# Azolo[1,5-*a*]pyrimidines and Their Condensed Analogs with Anticoagulant Activity

**DOI:** 10.3390/molecules27010274

**Published:** 2022-01-02

**Authors:** Konstantin V. Savateev, Victor V. Fedotov, Vladimir L. Rusinov, Svetlana K. Kotovskaya, Alexandr A. Spasov, Aida F. Kucheryavenko, Pavel M. Vasiliev, Vadim A. Kosolapov, Victor S. Sirotenko, Kseniya A. Gaidukova, Georgiy M. Uskov

**Affiliations:** 1 Department of Organic and Biomolecular Chemistry, Ural Federal University Named after the First President of Russia B.N. Eltsin, Mira St. 19, 620002 Yekaterinburg, Russia; victor0493@mail.ru (V.V.F.); v.l.rusinov@urfu.ru (V.L.R.); sk-kotovskaya-665@yandex.ru (S.K.K.); 2 Department of Pharmacology and Bioinformatics, Volgograd State Medical University, Pavshikh Bortsov Sq. 1, 400131 Volgograd, Russia; aspasov@mail.ru (A.A.S.); aidakucheryavenko@yandex.ru (A.F.K.); pvassiliev@mail.ru (P.M.V.); vad-ak@mail.ru (V.A.K.); sirotenko.viktor@yandex.ru (V.S.S.); ksenijagajjdukva@rambler.ru (K.A.G.); kastaban1@gmail.com (G.M.U.)

**Keywords:** azolo[1,5-*a*]pyrimidines, benzo[4,5]imidazo[1,2-*a*][1,2,3]triazolo[4,5-*e*]pyrimidines, nitrocompounds, anticoagulant, cytokine storm

## Abstract

Hypercytokinemia, or cytokine storm, is one of the severe complications of viral and bacterial infections, involving the release of abnormal amounts of cytokines, resulting in a massive inflammatory response. Cytokine storm is associated with COVID-19 and sepsis high mortality rate by developing epithelial dysfunction and coagulopathy, leading to thromboembolism and multiple organ dysfunction syndrome. Anticoagulant therapy is an important tactic to prevent thrombosis in sepsis and COVID-19, but recent data show the incompatibility of modern direct oral anticoagulants and antiviral agents. It seems relevant to develop dual-action drugs with antiviral and anticoagulant properties. At the same time, it was shown that azolo[1,5-*a*]pyrimidines are heterocycles with a broad spectrum of antiviral activity. We have synthesized a new family of azolo[1,5-*a*]pyrimidines and their condensed polycyclic analogs by cyclocondensation reactions and direct CH-functionalization and studied their anticoagulant properties. Five compounds among 1,2,4-triazolo[1,5-*a*]pyrimidin-7-ones and 5-alkyl-1,3,4-thiadiazolo[3,2-*a*]purin-8-ones demonstrated higher anticoagulant activity than the reference drug, dabigatran etexilate. Antithrombin activity of most active compounds was confirmed using lipopolysaccharide (LPS)-treated blood to mimic the conditions of cytokine release syndrome. The studied compounds affected only the thrombin time value, reliably increasing it 6.5–15.2 times as compared to LPS-treated blood.

## 1. Introduction

**Figure 1:**
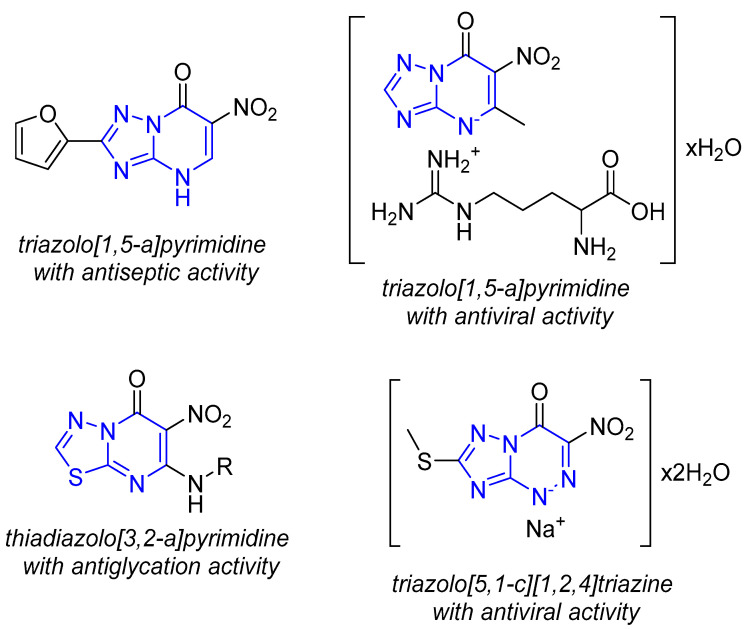
Examples of biologically active azoloazines.

Hypercytokinemia, or cytokine storm, is one of the severe complications of viral and bacterial infections, including COVID-19 caused by SARS-CoV-2. Activation of immune cells leads to damage of the endothelium of pulmonary vessels and, consequently, disrupts its protective functions, namely decreases the release of nitric oxide and PGI_2_, which suppress the activation and adhesion of leukocytes. Thrombin generation leads to the formation of fibrin, activation of platelets and endothelial cells through PAR-1 receptors, and increased production of von Willebrand factor (VWF); aggravates inflammation, causing activation of P-selectin; and activates leukocytes and smooth endothelial muscles, releasing multiple cytokines [1]. As a result, systemic endothelial dysfunction and sepsis-induced coagulopathy are associated with an increased risk of death, due to venous (about 70% of patients in critical condition) or arterial thromboembolic events, and, much less often, to hemorrhagic complications [2,3,4]. Disseminated intravascular coagulation (DIC) and systemic disorders lead to multiple organ failure, a characteristic of severe COVID-19. Anticoagulants hold one of the central positions among the means to prevent thrombosis in these conditions [5]. However, the use of currently available direct anticoagulants in COVID-19 is limited due to safety issues [6]. Therefore, the search for and development of novel pharmacological agents that reduce the thrombogenic potential of blood via coagulation and platelet activation management remain important and urgent tasks. Drugs that combine anticoagulant, antiviral, and/or antibacterial activity to reduce the risk of sepsis-induced coagulopathy and arterial thromboembolism are of special interest. At the same time, heterocyclic derivatives of the azoloazine nature and their condensed derivatives are known for their broad range of biological activity [7] (Figure 1). In particular, it was shown that these compounds have a pronounced protective effect against septic shock and influenza virus in vivo [7,8].

Consequently, we consider the study of azoloazine heterocycles as potential anticoagulant agents for the treatment of cytokine storm and, in particular, DIC promising and very relevant in the modern epidemic situation. Therefore, in the present paper, we propose a synthesis of the new derivatives of the azoloazine series: 6-ethoxycarbonyl- and 6-nitro-azolo[1,5-*a*]pyrimidines and 3-nitrobenzimidazo[1,2-*a*]pyrimidines and their structural analogs, polycyclic thiadiazolo[3,2-*a*]purines and benzimidazo[1,2-*a*]-1,2,3-triazolo[4,5-*e*]pyrimidines. The obtained compounds have been evaluated for their anticoagulant effect by in vitro/in vivo experiments. Additionally, the most important structural fragments responsible for this type of activity were identified based on iterative neural network pharmacophore analysis.

## 2. Results

### 2.1. Chemistry

**Scheme 1:**
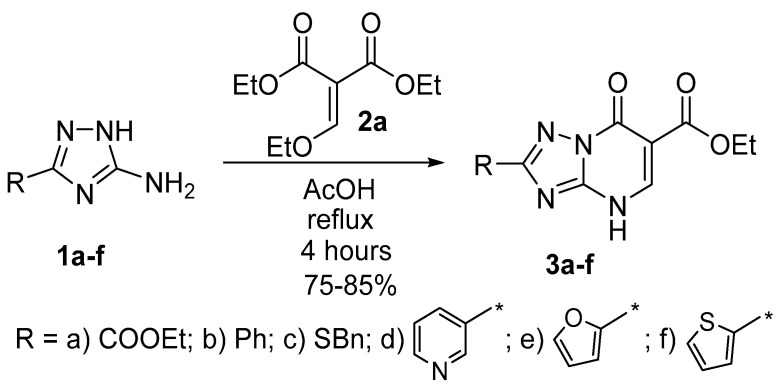
Synthesis of 6-ethoxycarbonyltriazolo[1,5-*a*]pyrimidin-7-ones **3a–f**.

It was shown previously that the nitro group plays an essential role in the azoloazine series for pronounced antiviral/antiseptic effect [7]. Considering this and structural similarity between nitro and ethoxycarbonyl fragments, we synthesized a series of a new azolo[1,5-*a*]pyrimidines **3a–l** by the reaction of commercially available aminoazoles **1a–h** and carbonyl-containing synthetic equivalents (e.g., diethyl ethoxymethylenemalonate **2a** and ethyl ethoxymethylenenitroacetate **2b**). A cyclocondensation of 3-*R*-5-amino-1,2,4-triazoles **1a–f** with diethyl ethoxymethylenemalonate **2a** was carried out at the reflux for 4 h in glacial acetic acid to form the target 6-ethoxycarbonyltriazolo[1,5-*a*]pyrimidines **3a–f** with excellent yields (75–85%). (Scheme 1).

**Scheme 2:**
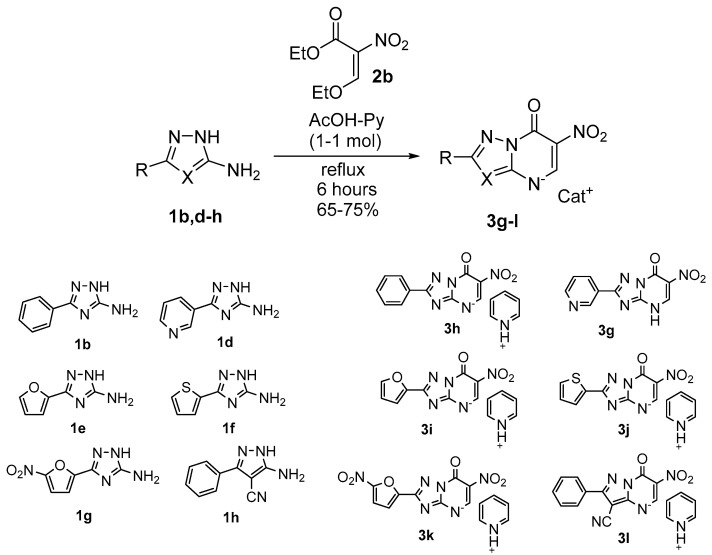
Synthesis of 6-nitroazolo[1,5-*a*]pyrimidin-7-ones **3g–l**.

On the contrary, the condensation of aminoazoles **1a–h** with ethyl ethoxymethylenenitroacetate **2b** in similar conditions leads to acylation products of the starting aminoazoles **1a–h**. At the same time, the desired 6-nitroazolo[1,5-*a*]pyrimidines **3g–l** were obtained with good yields (60–75%) when using an equimolar pyridine/acetic acid mixture as solvent (Scheme 2). It should be noted that the corresponding products **3h–l** were isolated as pyridinium salts, except for compound **3g**. This can be explained by the fact that 6-nitroazolo[1,5-*a*]pyrimidines are strong NH acids due to the electron-withdrawing effect of the nitrogroup on the heterocyclic system, while 2-(pyridin-3-yl)-6-nitrotriazolo[1,5-*a*]pyrimidine **3g** can exist as a zwitterion with minus charge at pyrimidine-nitrogen and plus charge at pyridine-nitrogen atoms.

**Scheme 3:**
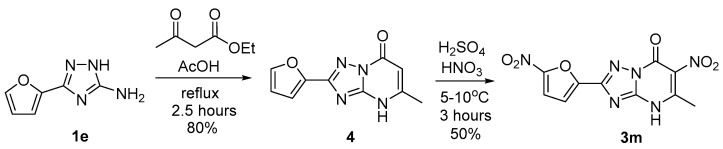
Two-step synthesis of dinitrotriazolopyrimidin-7-one **3m**.

An analog of azolopyrimidine **3k** with C5-methyl substituent was obtained by a two-step procedure. Initially, condensation of aminoazole **1e** with ethyl acetoacetate was carried out to form triazolopyrimidine **4** with good yield (80%). The following nitration of derivative **4** by nitric and sulfuric acids mixture led to the target dinitrotriazolo[1,5-*a*]pyrimidine **3m** (Scheme 3).

**Scheme 4:**
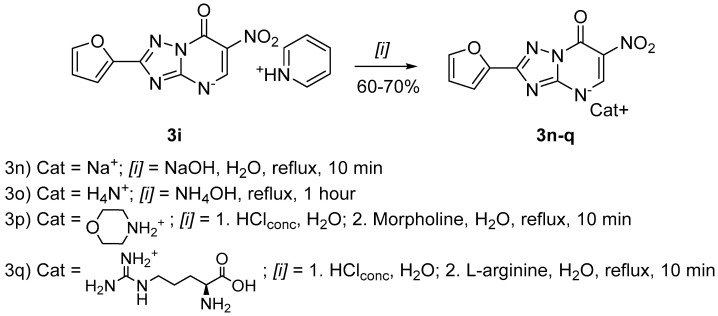
A series of water-soluble heterocycles **3n–q**.

Different salts of 2-(fur-2-yl)-6-nitrotriazolo[1,5-*a*]pyrimidin-7-one **3n–q** were obtained on the basis of heterocycle **3i** to establish the effect of the cation on anticoagulant activity and improve solubility in aqueous media (Scheme 4).

**Scheme 5:**
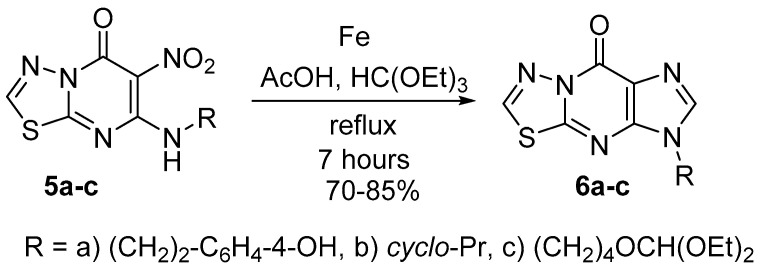
One-pot method for 5-alkylthiadiazolo[3,2-*a*]purin-8-ones **6a–c**.

In addition to azolo[1,5-*a*]pyrimidines **3a–q,** some condensed polycyclic systems were obtained. First of all, 5-alkylthiadiazolo[3,2-*a*]purines **6a–c** were synthesized starting from 5-alkylamino-6-nitrothiadiazolo[3,2-*a*]pyrimidin-7-ones **5a–c** by one-pot reduction of the nitro group and subsequent annulation of the imidazole fragment in the Fe-AcOH-HC(OEt)_3_ system (Scheme 5).

**Scheme 6:**
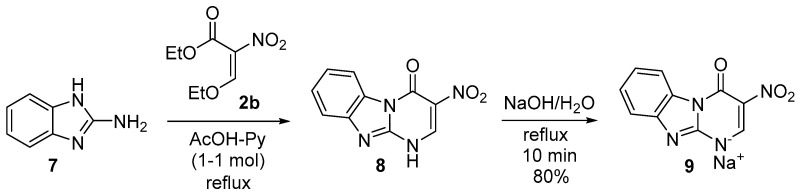
Synthesis of 3-nitrobenzimidazopyrimidinesodium salt **9**.

In addition, 3-Nitrobenzimidazo[1,2-*a*]pyrimidin-4(10H)-one **8** as structural analog of corresponding 6-nitroazolopyrimidine **3n** was obtained by a similar procedure, starting from aminobenzimidazole **7** (Scheme 6).

Finally, a pathway to C4-modified benzo[4,5]imidazo[1,2-*a*][1,2,3]triazolo[4,5-*e*]pyrimidines **13a–e, 14a** was revealed by means of CH functionalization. It was found that the reaction of benzimidazoazapurines **10a,b** with C-nucleophiles in CF_3_COOH proceeded smoothly with the formation of stable σH-adducts **11a–e, 12b** as trifluoroacetates.

**Scheme 7:**
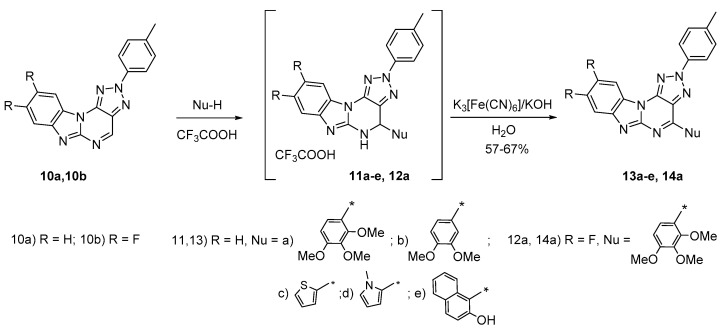
CH functionalization of the benzimidazoazapurines **10a**,**b**.

The following oxidation by K_3_[Fe(CN)_6_] (2 equiv.) in the basic solution of KOH (2 equiv.) led to the formation of S_N_^H^ (nucleophilic substitution of hydrogen) products **13a–e, 14b** with good yields (59–67%) (Scheme 7).

The purity and structure of all heterocycles were established by IR, ^1^H and ^13^C NMR, and elemental analysis (see Supplementary Materials).

### 2.2. Anticoagulant Activity of the Target Compounds In Vitro

Firstly, we studied direct oral anticoagulant drugs used in clinical practice—thrombin (IIa factor) inhibitor dabigatran etexilate (Boehringer Ingelheim Pharma GmbH and Co., Ingelheim am Rhein, Germany) and Xa factor inhibitor apixaban (Bristol Myers Squibb Manufacturing Company, Manati, Puerto Rico). The effect on coagulogram parameters was determined with in vitro experiments. We observed that dabigatran etexilate and apixaban at a concentration of 100 μM significantly increased the activated partial thromboplastin time (APTT) by 1.7 and 2.9 times relative to control, respectively (Table 1). Dabigatran etexilate in the studied concentration increased thrombin time (TT) by 6.3 times, which corresponds to the mechanism of its anticoagulant action—a disruption of the final stage of coagulation, while slightly increasing prothrombin time. Another anticoagulant drug apixaban increased prothrombin time (PT) by 4.6 times, which reflects the external activation pathway of the blood coagulation system and also indicates anticoagulant activity.

The study of the target azolo[1,5-*a*]pyrimidine derivatives and their condensed analogs was performed analogously, as an influence on the parameters of coagulogram of rabbit blood. The results are summarized in Table 1. It was shown that compound **3n** has the greatest ability to prolong thrombin time, exceeding the comparison drug dabigatran etexilate by 2.1 times. Compounds **3a** and **3m** were 1.9 times superior to dabigatran etexilate; **3k** was superior by 1.6 times. Compound **6c** was comparable in activity to dabigatran etexilate. Other substances also significantly prolonged thrombin time relative to control, but to a lesser extent than the comparison drug. None of the studied compounds affected the prothrombin time, unlike apixaban.

In order to expand the understanding of the possibility of using the most active substance 3n as a drug substance, an in silico assessment of its main pharmacokinetic parameters was carried out. The values of intestinal absorption (IA), volume of distribution (VD), and total clearance (Cl) were found using the pkCSM online resource [9]. The half-life t_1/2_ was determined using the ADMET utility of IT Microcosm system [8]. The following values of the indicated pharmacokinetic parameters were obtained: IA = 90.0%; VD = 0.106 L/kg; Cl = 4.539 mL/min/kg; t_1/2_ = 8–9 h.

### 2.3. Iterative Neural Network Pharmacophore Analysis

To identify the pharmacophore, an integral structural fragment that provides a high level of FIIa (thrombin serine proteases) inhibitory activity of the tested compounds, an iterative pharmacophore analysis was carried out using artificial neural networks, implemented according to the following scheme:

I. Preprocessing of the source data.

Structures of all tested compounds (**3a–14a**) (Table 1) were characterized with a matrix of QL descriptors of the 2nd rank of the 5th type using the IT Microcosm system [8]. This type of QL descriptor contains designations of two simple fragments of the structure of the compound (structural descriptors), which, due to electron donor or electron acceptor properties, can ensure the interaction of the molecule with the biological target; an example is the descriptor {NH_2_… > NH}. Paths between two structural descriptors can only pass through carbon chains. Based on the obtained QL descriptor matrix, with the addition of experimental values of FIIa inhibitory activity (TT values from Table 1), an initial training sample was formed, which was then used in the neural network modeling procedure.

II. Iterative neural network modeling.

Per Kolmogorov’s theorem [10] using a two-layer artificial neural network, a dependence of any complexity can be approximated, and it was necessary to ensure that signals from many input neurons were convoluted into a small number of intermediate images. Therefore, in the present work, the architecture of a two-layer perceptron with a narrow throat MLP k-m-1 was used in neural network modeling of regression dependence, where the number of input neurons k >> m of the number of hidden neurons. Calculations were performed using the Statistica package [11]. Iterative training of networks with selection of sensitive neurons was performed according to the following algorithm:

1. In the standard mode of the Statistica program, the initial dataset was divided into training, test, and validation sets in a 70/15/15% ratio. A total of 100 networks were trained with the automatic selection of 25 neural networks with high values of correlation coefficients.
2. Out of 25 optimal neural networks one best performing network was picked manually according to the set of three values of the correlation coefficients.
3. For the selected best neural network, sensitivity analysis of the input neurons was performed. The dimensionless sensitivity index Sens was calculated, which reflects the relative contribution of each neuron to the formation of the final signal of the output neuron.
4. If during sensitivity analysis neurons with Sens < 1.0 were found, they were removed from the initial training sample, and iterative neural network modeling was carried out, starting from step 1 of this scheme.
5. Otherwise, the process of iterative training of networks was completed, and for the best neural network, the overall accuracy of the prediction was assessed on the complete data set.
6. In the best neural network, the most sensitive input neurons with Sens ≥ 1.1 were identified, which corresponds to the QL descriptors most significantly affecting the level of FIIa-inhibitory activity of the studied compounds.

III. Post-processing of the data obtained.

1. Superposition of the significant QL descriptors found in stage II derive the pharmacophore, which provides a high level of FIIa-inhibitory activity of the tested compounds.
2. Analysis of entry of the constructed pharmacophore into the structure of the most active compounds was performed.

The best performing neural network was obtained after seven iterations, during which a total of about 1000 neural networks were trained and analyzed. The main characteristics of the results of each iteration are shown in Table 2.

For the best neural network model obtained as a result of the seventh iteration, the correlation coefficient on the combined dataset was R = 0.853 (*p* < 5 × 10^−7^).

**Figure 2:**
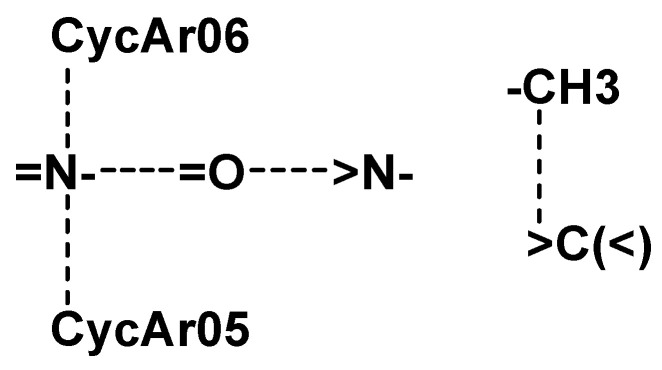
Pharmacophore that defines a high level of FIIa-inhibitory activity of novel compounds.

Five types of QL descriptors that correspond to neurons with Sens ≥ 1.1 and significantly affect the level of FIIa-inhibitory activity of new compounds were elucidated: {-N= … =O}, Sens = 1.22; {-N= … CycAr06}, Sens = 1.27; {-N= … CycAr05}, Sens = 1.23; {-N< … =O}, Sens = 1.10; {-CH_3_ … >C(<)}, Sens = 1.10. The combination of these binding points forms a pharmacophore that provides a high level of FIIa-inhibitory activity of the tested compounds (Figure 2).

Incorporation of the constructed pharmacophore into the structures of the three most active compounds and dabigatran etexilate is shown in Table 3.

The structure of compound **3a** includes a complete pharmacophore of nine entries of five types of QL descriptors of a high level of FIIa-inhibitory activity, compound **3m** contains seven entries of four types of QL descriptors of this pharmacophore, and in the structure of the compound **3n,** only six entries of three types of QL descriptors are present.

The structure of dabigatran also includes a complete pharmacophore with 18 entries of five types of QL descriptors of a high level of FIIa-inhibitory activity, and almost all of the found pharmacophore fragments occur in its molecule several times.

It is advisable to use the obtained data for the directed modification of the structure of the leading compounds in order to increase their activity. For example, the structure of dabigatran includes the pharmacophore fragment {CH3 … > C (<)}, which is absent in the structure of compound **3n**. Thus, it should be expected that the introduction of a sufficiently long aliphatic substituent (*n*-propyl, *n*-butyl, etc.) into the **3n** structure will lead to an increase in the anticoagulant activity of the modified compound.

### 2.4. Anticoagulant Activity after LPS Treatment

Sepsis is known as one of the severe complications of various microbial and viral infections, including COVID-19 caused by SARS-CoV-2, characterized by thromboinflammation [1]. Normally, this so-called immunocoagulation is a part of innate immunity and can serve as the first line of defense against infection. It is known that coagulation can be activated by external and internal pathways, resulting in fibrin formation. Preclinical and clinical studies have confirmed the pathological role of tissue factor, the initiator of the external pathway, in the development of endotoxemia [12,13]. It has been experimentally shown that exogenous lipopolysaccharide (LPS) can cause the expression and release of tissue factor on the surface of cells and lead to septic death of mice [14,15]. In addition, hypercytokinemia, which is observed during sepsis, causes not only activation of clotting factors, but also suppresses anticoagulant pathways, for example, the antithrombin system, activated protein C, and tissue factor inhibitor, thereby leading to DIC syndrome and fibrin deposition in blood vessels and tissues [16].

In this regard, compounds **3a, 3k, 3m, 3n**, which demonstrated the greatest antithrombin activity in the in vitro experiment, were investigated further for their effect on the parameters of coagulogram of rabbit blood treated with LPS to mimic conditions of hypercytokinemia (Table 4). Coagulation parameters of LPS-treated blood did not change, except for APTT, which was significantly lengthened by 1.2 times compared to the intact blood sample. At the same time, the reference drugs dabigatran etexilate and apixaban reliably prolonged APTT by 2.6 and 2.8 times, respectively. In addition, treatment with dabigatran etexilate significantly increased thrombin time by 1.8 times, and treatment with apixaban by 1.4 times. The studied compounds had a significant effect only on the thrombin time, reliably exceeding the control values of LPS-treated blood by 6.5–15.2 times.

As compounds **3a** and **3n** showed the greatest anticoagulant effect on LPS-treated blood, we determined their half-maximum effective concentrations (IC_50_) on intact and LPS-treated blood. As shown in Table 5, the potency of compounds **3a** and **3n** as the effect on thrombin time of intact blood exceeds the comparison drug by 1.8 and 1.6 times, respectively.

### 2.5. An Animal Study of Anticoagulant Activity

Compounds **3a** and **3n**, which demonstrated in vitro activity comparable to the reference drug dabigatran etexilate in the blood of intact animals both under conditions of hypercytokinemia and without, were studied in in vivo experiments on rats in doses equimolar to the dabigatran etexilate after a single intragastric administration. Parameters of the obtained coagulograms in experiments using animals blood at various time points are presented in Table 6.

We observed that compound **3a** did not affect the thrombin time 1, 2, and 4 h after intragastral administration. However, compound **3n** reliably prolonged this parameter by 1.4 times after 4 h only.

In addition, the efficiency of these compounds was investigated using thromboelastography analysis, and the results are shown in Table 7.

The reference drug dabigatran etexilate prolonged the time to the first evidence of clot formation by 3.6 times, increased the *K* value by 6.7 times, reduced the rate of clot formation by 4.4 times, and also reduced the maximum strength of the clot by 1.8 times relative to control values. Compounds **3a** and **3n** in doses equimolar to dabigatran during 4 h of observation did not have a significant effect on the time of formation of the first filaments of fibrin, the rate of clot formation, or the maximum amplitude. Compound **3n** 4 h after administration significantly increased the time from the beginning of clot formation until it reached an amplitude of 20 mm.

As the next step of the study, the ability of compounds **3a** and **3n** to affect coagulogram parameters in rats was assessed at 2× and 4× increased dose (of the dose equimolar to dabigatran etexilate), and results are shown in Table 8.

With a dose of **3n** increased by two times (11.6 mg/kg), a significant prolongation of thrombin time was observed 1, 2, and 4 h after intragastric administration. A further **3n** dose increase to 23.2 mg/kg resulted in the highest anticoagulant activity, comparable to 12 mg/kg dabigatran in terms of thrombin time, while other parameters of the TEG remained unaffected. Compound **3a** in a dose increased by two times did not affect any of the investigated parameters of the coagulogram.

Thromboelastography dose-elevating study of compound **3a** showed that the compound did not affect the parameters of the thromboelastogram, which corresponds to the data obtained in the rat blood coagulogram study (Table 9).

Compound **3n** in a dose 11.6 mg/kg (compared to the equimolar dose to dabigatran etexilate) showed the greatest activity 4 h after administration, as it prolonged the time to the first fibrin filaments formation by 1.9 times, increased the *K* value by 3.6 times, reduced the rate of clot formation by 2.5 times, and also reduced the maximum strength of the clot by 1.4 times relative to the control values.

With a further increase in the dose of the compound **3n** to 23.2 mg/kg, an anticoagulant effect was reliably observed 1 h after intragastric administration. After 4 h, TEG showed a statistically significant 4.1 times reduced rate of clot formation and 1.9 times reduced clot strength, which is comparable to the effect of dabigatran etexilate.

### 2.6. Acute Toxicity

After intraperitoneal injection of compound 3N in doses of 200–500 mg/kg, no mortality was observed in rats within 72 h.

### 2.7. Tail Bleeding Time (TBT)

Due to the pronounced anticoagulant activity and low toxicity (<500 mg/kg), compound **3n** was chosen to study its effect on bleeding time prolongation and was studied in a bleeding time model. As a result of this study test, the compound was shown to prolong bleeding time insignificantly compared to the control group and to be comparable to the reference drug dabigatran etexilate (Table 10).

## 3. Materials and Methods

### 3.1. Chemistry

Commercial reagents were obtained from Sigma-Aldrich, Acros Organics, or Alfa Aesar (Sigma-Aldrich, St Louis, MO, USA) and used without any further purification. All workup and purification procedures were carried out using analytical grade solvents. One-dimensional ^1^H and ^13^C NMR spectra were acquired on a Bruker DRX-400 instrument (Karlsruhe, Germany) (400 and 101 MHz, respectively), utilizing CDCl_3_ and DMSO-*d*_6_ as solvent and as an external reference. The following abbreviations are used for multiplicity of NMR signals: s—singlet, d—doublet, t—triplet, q—quartet, dd—double of doublets, m—multiplet, br—broaded. IR spectra were recorded on a Bruker Alpha spectrometer equipped with a ZnSe ATR accessory. Elemental analysis was performed on a PerkinElmer PE 2400 (Waltham, MA, USA) elemental analyzer. Melting points were determined on a Stuart SMP3 (Staffordshire, UK) and are uncorrected. The monitoring of the reaction progress was performed by using TLC on Sorbfil plates (Imid LTD, Russia, Krasnodar) eluent is CHCl_3_. Heterocycles **3b, 3d, 3e, 7, 10a, 10b** were synthesized in accordance with literature data: **3b [17]**, **3d, 3e [18]**, **7 [19]**, **10a, 10b [20]**. All synthesized compounds are >95% pure by elemental analysis.

General procedure for the synthesis of 2-R-6-ethoxycarbonyl-1,2,4-triazolo[1,5-a]pyrimidin-7-ones (**3a, 3f**).

A suspension of 0.0030 mol (1 equiv.) of the corresponding 3-*R*-5-amino-1,2,4-triazole **1a, 1f** and 0.0032 mol (1.07 equiv.) of diethyl ethoxymethylenemalonate **2a** in 6.0 mL of glacial AcOH was stirred at reflux (130 °C oil bath temperature) for 5 h. The reaction mixture was cooled to 25 °C, and the obtained precipitate was filtered off and washed with 10 mL of *i*-PrOH to give the analytical pure product.

2,6-Diethoxycarbonyl-1,2,4-triazolo[1,5-a]pyrimidin-7-one (**3a**): White powder (1.04 g, yield 75%), m.p. 266–267 °C. FT-IR (neat) ν_max_ (cm^−1^): 1764 (C=O), 1732 (C=O), 1642 (C=O). ^1^H NMR (400 MHz, DMSO-*d*_6_) *δ* (ppm) 1.29 (3H, t, *J* = 7.1 Hz, OCH_2_CH_3_), 1.34 (3H, t, *J* = 7.1 Hz, OCH_2_CH_3_), 4.26 (2H, q, *J* = 7.1 Hz, OCH_2_CH_3_), 4.39 (2H, q, *J* = 7.1 Hz, OCH_2_CH_3_), 8.72 (1H, s, H-2). ^13^C{^1^H} NMR (100 MHz, DMSO-*d*_6_) *δ* (ppm) 14.0, 14.2, 60.5, 61.7, 102.9, 147.7, 150.9, 152.8, 153.6, 159.3, 162.9. Anal. Calcd. for C_11_H_12_N_4_O_5_: C 47.15, H 4.32, N 19.99; found: C 47.22, H 4.29, N 20.09.

2-(Thien-2-yl)-6-ethoxycarbonyl-1,2,4-triazolo[1,5-a]pyrimidin-7-one (**3f**)**:** White powder (0.74 g, yield 85%), m.p. > 300 °C. FT-IR (neat) ν_max_ (cm^−1^): 1739 (C=O), 1699 (C=O), 1630 (C=O). ^1^H NMR (400 MHz, DMSO-*d*_6_) *δ* (ppm) 1.28 (3H, t, *J* = 7.2 Hz, OCH_2_CH_3_), 4.25 (2H, q, *J* = 7.2 Hz, OCH_2_CH_3_), 7.22 (1H, t, *J* = 4.0 Hz, H-4′), 7.74–7.80 (2H, m, H-3′, H-5′), 8.63 (1H, s, H-5). ^13^C{^1^H} NMR (100 MHz, DMSO-*d*_6_) *δ* (ppm) 14.2, 60.2, 102.4, 128.1, 128.3, 129.1, 132.7, 147.9, 151.9, 153.0, 157.5, 163.3. Anal. Calcd. for C_12_H_10_N_4_O_3_S: C 49.65 H 3.47 N 19.30; found: C 49.37 H 3.48 N 19.34.

General procedure for the synthesis of 6-nitroazolo[1,5-a]pyrimidin-7-ones (**3g–l**)**:** Ethyl ethoxymethylenenitroacetate **2b** (0.01 mol, 1 equiv.) was added to a stirred solution of the corresponding 5-amino-1,2,4-triazole **1b, 1d–h** or 3-phenyl-4-cyano-5-aminopyrazole **1g** (0.01 mol, 1 equiv.) in a mixture of pyridine (8.5 mL) and glacial acetic acid (6.0 mL) and the resulting solution was stirred at reflux (145 °C oil bath temperature) for 6 h. The obtained precipitate was filtered off and washed with of EtOH.

2-(Pyridin-3-yl)-6-nitro-1,2,4-triazolo[1,5-a]pyrimidin-7-one (**3g**): Pale yellow powder (1.81 g, yield 70%), m.p. > 300 °C. FT-IR (neat) ν_max_ (cm^−1^): 1675 (C=O), 1583 (NO_2_), 1264 (NO_2_). ^1^H NMR (400 MHz, DMSO-*d*_6_) *δ* (ppm) 7.48 (1H, dd, *J* = 8.0, 4.4 Hz, H-5′), 8.47 (1H, d, *J* = 8.0 Hz, H-4′), 8.61 (1H, d, *J* = 4.4 Hz, H-6′), 8.99 (1H, s, H-2′), 9.30 (1H, c, H-5). ^13^C{^1^H} NMR (100 MHz, DMSO-*d*_6_) *δ* (ppm) 123.2, 123.9, 127.0, 134.0, 147.6, 150.7, 151.0, 153.5, 159.0, 160.1. Anal. Calcd. for C_10_H_6_N_6_O_3_: C 46.52, H 2.34, N 32.55; found: C 46.59, H 2.20, N 32.58.

Pyridinium 2-phenyl-6-nitro-1,2,4-triazolo[1,5-a]pyrimidin-7-one (**3h**): Yellow powder (0.92 g, yield 70%), m.p. > 300 °C. FT-IR (neat) ν_max_ (cm^−1^): 1680 (C=O), 1629 (NO_2_), 1280 (NO_2_). ^1^H NMR (400 MHz, DMSO-*d*_6_) *δ* (ppm) 7.51 (3H, m, H-3′, H-4′, H-5′), 7.93 (2H, m, H-2′, H-6′), 8.13 (2H, d, *J* = 8.0 Hz, H-3′′, H-5′′), 8.43 (1H, m, H-4′′), 8.85 (2H, d, *J* = 5.6 Hz, H-2′′, H-6′′), 9.07 (1H, s, H-5). ^13^C{^1^H} NMR (100 MHz, DMSO-*d*_6_) *δ* (ppm) 123.7, 126.5 (2C), 126.6 (2C), 128.8 (2C), 130.0, 130.6, 143.6 (2C), 144.5, 150.4, 152.0, 157.4, 161.5. Anal. Calcd. for C_16_H_12_N_6_O_3_: C 57.14, H 3.60, N 24.99; found: 57.01, H 3.85, N 24.80.

Pyridinium 2-(fur-2-yl)-6-nitro-1,2,4-triazolo[1,5-a]pyrimidin-7-one (**3i**): Yellow powder (0.65 g, yield 60%), m.p. 225–226 °C. FT-IR (neat) ν_max_ (cm^−1^): 1683 (C=O), 1616 (NO_2_), 1289 (NO_2_). ^1^H NMR (400 MHz, DMSO-*d*_6_) *δ* (ppm) 6.63 (1H, dd, *J* = 3.6, 1.6 Hz, H-4′), 7.08 (1H, d, *J* = 3.6 Hz, H-3′), 7.78 (1H, d, *J* = 1.6 Hz, H-5′), 7.85 (2H, t, *J* = 6.8 Hz, H-3′′, H-5′′), 8.33 (1H, t, *J* = 6.8 Hz, H-4′′), 8.84 (2H, d, *J* = 4.8 Hz, H-2′′, H-6′′), 9.04 (1H, s, H-5). ^13^C{^1^H} NMR (100 MHz, DMSO-*d*_6_) *δ* (ppm) 111.0, 111.9, 123.6, 126.7, 143.3, 143.4, 144.6, 144.8, 144.9, 146.1, 150.3, 152.3, 155.1, 157.4. Anal. Calcd. for C_14_H_10_N_6_O_4_: C 51.54, H 3.09, N 25.76; found: 51.48, H 3.10, N 25.80.

Pyridinium 2-(thien-2-yl)-6-nitro-1,2,4-triazolo[1,5-a]pyrimidin-7-one (**3j**): Yellow powder (2.22 g, yield 65%), m.p. > 300 °C. FT-IR (neat) ν_max_ (cm^−1^): 1670 (C=O), 1527 (NO_2_), 1280 (NO_2_). ^1^H NMR (400 MHz, DMSO-*d*_6_) *δ* (ppm) 7.16 (1H, dd, *J* = 4.8, 4.8 Hz, H-4′), 7.58 (1H, d, *J* = 4.8 Hz, H-3′), 7.75 (1H, d, *J* = 4.8 Hz, H-5′), 7.83 (2H, m, H-3′′, H-5′′), 8.31 (1H, t, *J* = 8.0 Hz, H-4′′), 8.81 (2H, d, *J* = 5.6 Hz, H2′′, H-6′′), 9.05 (1H, s, H-5). ^13^C{^1^H} NMR (100 MHz, DMSO-*d*_6_) *δ* (ppm) 123.6, 126.7 (2C), 127.4, 128.1, 128.4, 133.7, 143.5 (2C), 144.6, 150.3, 152.3, 157.5, 158.2. Anal. Calcd. for C_14_H_10_N_6_O_3_S: C 49.12, H 2.94, N 24.55; found: C 49.01, H 3.01, N 24.60.

Pyridinium 2-(5-nitrofur-2-yl)-6-nitro-1,2,4-triazolo[1,5-a]pyrimidin-7-one (**3k**): Brown powder (1.39 g, yield 75%), m.p. 282–284 °C. FT-IR (neat) ν_max_ (cm^−1^): 3046, 3138, 1693 (C=O), 1605 (NO_2_), 1540 (NO_2_), 1305 (NO_2_), 1335 (NO_2_). ^1^H NMR (400 MHz, DMSO-*d*_6_) *δ* (ppm) 7.11 (1H, d, *J* = 3.2 Hz, H-4′), 7.85 (1H, d, *J* = 4.8 Hz, H-3′), 7.99 (2H, t, *J* = 6.4 Hz, H-3′′, H-5′′), 8.50 (1H, t, *J* = 8.0 Hz, H-4′′), 8.91 (2H, d, *J* = 4.8 Hz, H2′′, H-6′′), 9.07 (1H, s, H-5). ^13^C{^1^H} NMR (100 MHz, DMSO-*d*_6_) *δ* (ppm) 114.0, 115.1, 124.1, 127.2, 143.9, 145.2, 148.9, 150.8, 152.3, 153.9, 154.0, 159.0. Anal. Calcd. for C_14_H_9_N_7_O_6_: C 45.29 H 2.44 N 26.41; found: C 45.45 H 2.45 N 26.59.

Pyridinium 2-phenyl-3-cyano-6-nitropyrazolo[1,5-a]pyrimidin-7-one (**3l**): Yellow crystals (1.30 g, yield 60%), m.p. 249–251 °C. FT-IR (neat) ν_max_ (cm^−1^): 2217 (CN), 1675 (C=O), 1548 (NO_2_), 1282 (NO_2_). ^1^H NMR (400 MHz, DMSO-*d*_6_) *δ* (ppm) 7.57 (3H, m, H-3′, H-4′, H-5′), 8.00 (4H, m, H-2′′, H3′′, H-5′′, H-6′′), 8.50 (1H, t, *J* = 8.0 Hz, H-4′′), 8.88 (2H, д, *J* = 6.0 Hz, H-2′, H-6′), 8.93 (1H, s, H-5). ^13^C{^1^H} NMR (100 MHz, DMSO-*d*_6_) *δ* (ppm) 78.6, 114.9, 123.1, 126.7 (2C), 127.1 (2C), 129.0 (2C), 129.9, 131.0, 142.5 (2C), 146.0, 150.1, 151.5, 153.6, 155.4.Anal. Calcd. for C_18_H_12_N_6_O_3_: C 60.00, H 3.36, N 23.32; found: C 59.82, H 3.39, N 23.20.

2-(5-Nitrofur-2-yl)-5-methyl-6-nitro-1,2,4-triazolo[1,5-a]pyrimidin-7-one (**3m**): First, 2.02 mL (0.016 mol, 1.23 equiv.) of ethyl acetoacetate was added to a suspension of 2.00 g (0.013 mol, 1 equiv.) of 3-(fur-2-yl)-5-amino-1,2,4-triazole **1e** in 10 mL of glacial AcOH. The resulting mixture was stirred for 2.5 h at reflux (130 °C oil bath temperature). The reaction mixture was cooled to 25 °C, and the obtained precipitate was filtered off, washed with 10 mL of *i*-PrOH, and air dried to give 2.16 g of **4**. This solid was dissolved in 20 mL of 94% H_2_SO_4_ at 25 °C. Then, 1.98 mL (0.031 mol) of 70% HNO_3_ was added to the resulting solution, maintaining the temperature of the reaction mixture at 5–10 °C. The reaction mixture was stirred for 3 h at 25 °C. The resulting mixture was poured into ice water. The solid product formed was collected by filtration and washed with H_2_O. Yellow powder (1.53 g, yield 50%), m.p. > 300 °C. FT-IR (neat) ν_max_ (cm^−1^): 1729 (C=O), 1640 (NO_2_), 1346 (NO_2_), 1314 (NO_2_). ^1^H NMR (400 MHz, DMSO-*d*_6_) *δ* (ppm) 2.58 (3H, s, CH_3_) 7.47 (1H, d, *J* = 4.0 Hz, H-3), 7.75 (1H, d, *J* = 4.0 Hz, H-4′). ^13^C{^1^H} NMR (100 MHz, DMSO-*d*_6_) *δ* (ppm) 18.3, 114.4, 114.8, 127.8, 146.6, 149.4, 150.8, 152.2, 152.9, 153.3. Anal. Calcd. for C_10_H_6_N_6_O_6_*3H_2_O: C 33.34, H 3.36, N 23.33; found: C 33.44, H 3.42, N 23.14.

2-(Fur-2-yl)-6-nitro-1,2,4-triazolo[1,5-a]pyrimidin-7-one sodium salt (**3n**): First, 6.55 g (0.020 mol, 1 equiv.) of pyridinium 2-(fur-2-yl)-6-nitro-1,2,4-triazolo[1,5-*a*]pyrimidin-7-one **3i** was added to the solution of 1.28 g (0.032 mol, 1.6 equiv.) of NaOH in 65 mL H_2_O. The resulting suspension was refluxed for 10 min and cooled to 25 °C. The solid product formed was collected by filtration and recrystallized from H_2_O. Yellow crystals (3.88 g, yield 60%), m.p. 259–261 ^o^C. FT-IR (neat) ν_max_ (cm^−1^): 3358 (H_2_O), 1661 (C=O), 1539 (NO_2_), 1256 (NO_2_). ^1^H NMR (400 MHz, DMSO-*d*_6_) *δ* (ppm) 6.66 (1H, dd, H-4′), 7.10 (1H, d, H-3′), 7.83–7.89 (1H, br.m, H-5′), 9.01 (1H, s, H-5). ^13^C{^1^H} NMR (100 MHz, DMSO-*d*_6_) *δ* (ppm) 110.9, 111.9, 123.2, 144.5, 146.4, 150.9, 153.5, 155.6, 158.7. Anal. Calcd. for C_9_H_4_N_5_O_4_Na_*_H_2_O: C 37.64 H 2.11 N 24.39; found: C 37.73 H 2.09 N 24.42.

2-(Fur-2-yl)-6-nitro-1,2,4-triazolo[1,5-a]pyrimidin-7-one ammonium salt (**3o**): A mixture of 1.00 g (0.0031 mol) of 2-(fur-2-yl)-6-nitro-1,2,4-triazolo[1,5-*a*]pyrimidin-7-one pyridinium **3i** and 10 mL of 25% ammonia aqueous solution was refluxed for 1 h. The reaction mixture was cooled to 25 °C. The formed solid product was collected by filtration and washed with of H_2_O. Gray powder (0.49 g, yield 60%), m.p. > 300 °C. FT-IR (neat) ν_max_ (cm^−1^): 3181 (NH_4_), 1667 (C=O), 1536 (NO_2_), 1250 (NO_2_). ^1^H NMR (400 MHz, DMSO-*d*_6_) *δ* (ppm) 6.63–6.69 (1H, m, H-4′), 7.05–7.11 (1H, m, H-3′), 7.15 (4H, t, NH_4_), 7.86 (1H, s, H-5′), 9.01 (1H, s, H-5). ^13^C{^1^H} NMR (100 MHz, DMSO-*d*_6_) *δ* (ppm) 110.8, 111.9, 123.2, 144.5, 146.5, 150.8, 153.5, 155.5, 158.7. Anal. Calcd. for C_9_H_8_N_6_O: C 40.91, H 3.05, N 31.81; found: C 41.00, H 3.10, N 31.99.

2-(Fur-2-yl)-6-nitro-1,2,4-triazolo[1,5-a]pyrimidin-7-one morpholinic salt (**3p**): A suspension of 0.67 g (0.0021 mol, 1 equiv.) of 2-(fur-2-yl)-6-nitro-1,2,4-triazolo[1,5-*a*]pyrimidin-7-one pyridinium **3i** in 5 mL H_2_O was acidified with 37% HCl to pH ~1. The formed precipitate was collected by filtration, washed with H_2_O, and air dried. The resulting solid was suspended in 8 mL of H_2_O, and 0.18 mL (0.0021 mol, 1 equiv.) of morpholine was added. The resulting mixture was heated until a clear solution was formed. The reaction mixture was cooled to 25 °C. The solid product formed was collected by filtration and washed with EtOH. Yellow powder (0.35 g, yield 65%), m.p. 204–205 ^o^C. FT-IR (neat) ν_max_ (cm^−1^): 3408 (NH_2_), 1670 (C=O), 1534 (NO_2_), 1244 (NO_2_). ^1^H NMR (400 MHz, DMSO-*d*_6_) *δ* (ppm) 3.13 (4H, m, 2хCH_2_), 3.75 (4H, m, 2хCH_2_), 6.63-6.69 (1H, m, H-4′), 7.05-7.11 (1H, m, H-3′), 7.86 (1H, s, H-5′), 8.71 (2H, br. s, NH_2_), 9.01 (1H, s, H-5). ^13^C{^1^H} NMR (100 MHz, DMSO-*d*_6_) *δ* (ppm) 43.0, 63.3, 110.8, 111.9, 123.2, 144.4, 146.5, 150.7, 153.4, 155.4, 158.7. Anal. Calcd. for C_13_H_14_N_6_O_5_: C 46.71, H 4.22, N 25.14; found: C 46.95, H 4.11, N 25.30.

2-(Fur-2-yl)-6-nitro-1,2,4-triazolo[1,5-a]pyrimidin-7-one l-argininium salt (**3q**): A suspension of 0.67 g (0.0021 mol, 1 equiv.) of 2-(fur-2-yl)-6-nitro-1,2,4-triazolo[1,5-*a*]pyrimidin-7-one pyridinium **3i** in 5 mL H_2_O was acidified with 37% HCl to pH ~1.The formed precipitate was collected by filtration, washed with H_2_O, and air dried. The resulting solid was added to a solution of 0.36 g (0.0021 mol, 1 equiv.) l-arginine in 5 mL of H_2_O. The resulting mixture was heated until a clear solution was formed. The reaction mixture was cooled to 25 °C. The solid product was collected by filtration and washed with EtOH. Yellow powder (0.57 g, yield 65%), m.p. 229–230 °C. FT-IR (neat) ν_max_ (cm^−1^): 3107 (NH_2_), 1664 (C=O), 1619 (C=O), 1534 (NO_2_), 1247 (NO_2_). ^1^H NMR (400 MHz, DMSO-*d*_6_) *δ* (ppm) 1.46–1.71 (4H, br. m, 2×CH_2_), 1.70–1.83 (1H, br.m, CH), 3.10 (2H, br.m, CH_2_), 3.34 (2H, br.m, NH_2_), 6.62-6.70 (1H, m, H-4′), 7.07 (1H, d, *J* = 3. Hz, H-3′), 7.53 (5H, br. s, NH, 2×NH_2_), 7.86 (1H, s, H-5′), 9.00 (1H, s, H-5). ^13^C{^1^H} NMR (100 MHz, DMSO-*d*_6_) *δ* (ppm) 24.8, 28.2, 53.6, 110.7, 111.9, 123.2, 144.4, 146.5, 150.7, 153.4, 155.4, 157.2, 158.7, 172.42. Anal. Calcd. for C_15_H_19_N_9_O_6_: C 42.76, H 4.54, N 29.92; found: C 42.90, H 4.33, N 29.99.

General procedure for the synthesis of 3-R-[1,3,4]thiadiazolo[3,2-a]purin-9(3H)-one (**6a-c**): Fe dust (5.6 g; 0.1 mol, 10 equiv.) was added to a stirred solution of the corresponding 5-alkylamino-6-nitro-[1,3,4]thiadiazolo[3,2-*a*]pyrimidin-7-one (0.01 mol, 1 equiv.) **5a-c** in a mixture of 45 mL of glacial AcOH and 45 mL of triethyl orthoformate. The mixture was stirred at reflux (145 °C oil bath temperature) for 7 h. The reaction mixture was cooled to 25 °C and filtered. The filtrate was evaporated to dryness at 45 °C in a vacuum, and 100 mL of H_2_O was added to the residue. The precipitate formed was filtered off and air dried.

3-(4-Hydroxyphenylethyl)-[1,3,4]thiadiazolo[3,2-a]purin-8-one (**6a**): Yellow powder (2.50 g, yield 80%), m.p. 234–235 °C. FT-IR (neat) ν_max_ (cm^−1^): 3100, 2743, 1689, 1504, 1376, 1281. ^1^H NMR (400 MHz, DMSO-*d*_6_) *δ* (ppm) 3.00 (2H, t, *J* = 7.2 Hz, NCH_2_CH_2_), 4.33 (2H, t, *J* = 7.2 Hz, NCH_2_CH_2_), 6.62 (2H, d, *J* = 8.0 Hz, H-3′, H-5′), 6.91 (2H, d, *J* = 8.0 Hz, H-2′, H-6′), 7.80 (1H, s, H-6), 9.23 (1H, s, H-2), 11.77 (1H, s, OH). ^13^C{^1^H} NMR (100 MHz, DMSO-*d*_6_) *δ* (ppm) 34.4, 45.0, 115.2 (2C), 119.8, 127.7, 129.6 (2C), 140.9, 147.1, 147.9, 152.2, 156.0, 157.7. Anal. Calcd. for C_14_H_11_N_5_O_2_S: C 53.67, H 3.54, N 22.35; found: C 53.65, H 3.61, N 22.22.

3-(Cyclopropyl)-[1,3,4]thiadiazolo[3,2-a]purin-8-one (**6b**): White powder (2.50 g, yield 80%), m.p. 272–274 °C. FT-IR (neat) ν_max_ (cm^−1^): 3077, 1709, 1531, 1488, 1315, 1233. ^1^H NMR (400 MHz, DMSO-*d*_6_) *δ* (ppm) 1.13 (4H, d, *J* = 4.0 Hz, (CH_2_)_2_), 3.45-3.51 (1H, m, NCH), 8.00 (1H, s, H-6), 9.24 (1H, s, H-2). ^13^C{^1^H} NMR (100 MHz, DMSO-*d*_6_) *δ* (ppm) 5.4, 25.3, 120.0, 140.5, 146.7, 149.0, 151.9, 157.4. Anal. Calcd. for C_9_H_7_N_5_OS: C 46.34, H 3.03, N 30.03; found: C 46.29, H 2.98, N 30.19.

5-[4-(Diethoxymethyloxy)butyl]thiadiazolo[3,2-a]purin-8-one (**6c**): White powder (2.57 g, yield 70%), m.p. 145–147 °C. FT-IR (neat) ν_max_ (cm^−1^): 3056, 1705, 1534, 1491, 1367, 1180. ^1^H NMR (400 MHz, DMSO-*d*_6_) *δ* (ppm) 1.08 (6H, t, *J* = 8.0 Hz, 2xCH_3_), 1.46–1.52 (2H, m, CH_2_), 1.83–1.89 (2H, m, CH_2_), 3.43–3.49 (6H, m, 3×OCH_2_), 4.18 (2H, t, *J* = 8.0 Hz, NCH_2_), 5.10 (1H, s, OCH), 8.16 (1H, s, H-6), 9.26 (1H, s, H-2). ^13^C{^1^H} NMR (100 MHz, DMSO-*d*_6_) *δ* (ppm) 14.9 (2C), 26.1, 26.4, 43.2, 58.9, 62.5, 111.9, 119.9, 140.9, 147.1, 148.0, 152.1, 157.6. Anal. Calcd. for C_15_H_21_N_5_O_4_S: C 49.03 H 5.76 N 19.06; found: C 49.04 H 5.70 N 19.00.

3-Nitrobenz[4,5]imidazo[1,2-a]pyrimidin-4(1H)-one sodium salt (**9**): First, 1.15 g (0.005 mol, 1 equiv.) of 3-nitrobenz[4,5]imidazo[1,2-*a*]pyrimidin-4(1*H*)-one **8** was added to a stirred solution of the 0.20 g (0.005 mol, 1 equiv.) of NaOH in 20 mL of H_2_O. The resulting mixture was heated until a clear solution was formed. The reaction mixture was cooled to 25 °C. The solid product formed was collected by filtration and washed with EtOH. Yellow powder (1.01 g, yield 80%), m.p. > 300 °C. FT-IR (neat) ν_max_ (cm^−1^): 3109, 1692, 1611, 1547, 1450, 1297. ^1^H NMR (400 MHz, DMSO-*d*_6_) *δ* (ppm) 7.23 (1H, d, *J* = 7.6 Hz, H-7), 7.35 (1H, t, *J* = 7.7 Hz, H-8), 7.61 (1H, t, *J* = 8.0 Hz, H-6), 8.42 (1H, t, *J* = 8.1 Hz, H-9), 9.05 (1H, s, H-2). ^13^C{^1^H} NMR (100 MHz, DMSO-*d*_6_) *δ* (ppm) 115.2, 118.0, 120.8 120.9, 124.2, 129.8, 143.5, 154.2, 154.35, 154.43. Anal. Calcd. for C_10_H_5_N_4_NaO_3_: C 47.63, H 2.00, N 22.22; found: C 47.65, H 1.98, N 22.19.

General procedure for the synthesis of 2-(p-tolyl)-4-(R)-2H-benz[4,5]imidazo[1,2-a][1,2,3] triazolo[4,5-e]pyrimidines (**13a–e, 14a**): To a stirred solution of 0.30 g (0.001 mol, 1 equiv.) of 2-(*p*-tolyl)-2*H*-benz[4,5]imidazo[1,2-*a*][1,2,3]triazolo[4,5-*e*]pyrimidine **10a, 10b** in 5 mL of CF_3_COOH, the corresponding nucleophilic agent 0.001 mol (1 equiv.) **NuH** was added. The resulting solution was stirred at 25 °C for 5 h. The reaction mixture was concentrated under reduced pressure, 10 mL of 1,4-dioxane was added to the residue, and then a solution of 0.66 g (0.002 mol) of K_3_[Fe (CN)_6_] and 0.11 g (0.002 mol, 2 0.001 mol (1 equiv.) of KOH in 20 mL H_2_O was added. The resulting mixture was stirred for 5 h at 25 °C and extracted with CHCl_3_ (2 × 20 mL). The combined organic layer was dried over anhydrous Na_2_SO_4_ and concentrated under reduced pressure. The residue was purified by column chromatography on silica gel 60 using CHCl_3_ as eluent to give the corresponding pure product.

2-(p-Tolyl)-4-(2,3,4-trimethoxyphenyl)-2H-benz[4,5]imidazo[1,2-a][1,2,3]triazolo[4,5-e]pyrimidine (**13a**): Yellow powder (0.27 g, yield 57%), m.p. 226–228 °C. FT-IR (neat) ν_max_ (cm^−1^): 2935, 1631, 1593, 1479, 1292, 1094. ^1^H NMR (400 MHz, CDCl_3_) *δ* (ppm) 2.44 (3H, s, CH_3_), 3.95 (3H, s, OCH_3_), 3.99 (3H, s, OCH_3_), 4.13 (3H, s, OCH_3_), 6.89 (1H, d, *J* = 8.8 Hz, H-5′), 7.34 (2H, d, *J* = 8.3 Hz, H-3′′, H-5′′), 7.54–7.59 (2H, m, H-8, H-9), 7.74 (1H, d, *J* = 8.8 Hz, H-6′), 8.02–8.10 (1H, m, H-10), 8.18 (2H, d, *J* = 8.2 Hz, H-2′′, H-6′′), 8.32–8.39 (1H, m, H-7). ^13^C{^1^H} NMR (100 MHz, CDCl_3_) *δ* (ppm) 21.3, 56.3, 61.0, 62.4, 107.4, 113.2, 119.9 (2C), 120.9, 122.8, 124.0, 125.3, 126.9, 127.9, 130.3 (2C), 130.9, 137.5, 139.8, 142.7, 144.0, 145.8, 150.3 153.5, 156.2, 156.7. Anal. Calcd. for C_26_H_22_N_6_O_3_: C 66.94, H 4.75, N 18.02; found: C 67.15, H 4.83, N 17.86.

2-(p-Tolyl)-4-(3,4-trimethoxyphenyl)-2H-benz[4,5]imidazo[1,2-a][1,2,3]triazolo[4,5-e]pyrimidine (**13b**): Yellow powder (0.27 g, yield 62%), m.p. 257–259 °C. FT-IR (neat) ν_max_ (cm^−1^): 2933, 1678, 1631, 1598, 1482, 1271. ^1^H NMR (400 MHz, CDCl_3_) *δ* (ppm) 2.44 (3H, s, CH_3_), 3.97 (3H, s, OCH_3_), 4.03 (3H, s, OCH_3_), 6.97 (1H, d, *J* = 8.5 Hz, H-5′), 7.33 (2H, d, *J* = 8.3 Hz, H-3′′, H-5′′), 7.47-7.55 (2H, m, H-8, H-9), 7.98 (1H, d, *J* = 7.7 Hz, H-10), 8.16 (2H, d, *J* = 8.0 Hz, H-2′′, H-6′′), 8.25 (1H, d, *J* = 7.6 Hz, H-7), 8.39 (1H, s, H-2′), 8.66 (1H, d, *J* = 8.4 Hz, H-2′). ^13^C{^1^H} NMR (100 MHz, CDCl_3_) *δ* (ppm) 21.3, 56.0, 56.1, 110.6, 111.0, 113.0, 119.5 (2C), 120.4, 123.7, 124.9, 125.3, 127.4, 127.8, 129.5, 130.2 (2C), 137.0, 139.8, 143.7, 146.9, 149.3, 149.9, 153.1, 153.7. Anal. Calcd. for C_25_H_20_N_6_O_2_: C 68.80, H 4.62, N 19.25; found: C 68.96, H 4.63, N 19.12.

2-(p-Tolyl)-4-(thien-2-yl)-2H-benz[4,5]imidazo[1,2-a][1,2,3]triazolo[4,5-e]pyrimidine (**13c**): Orange powder (0.25 g, yield 65%), m.p. > 300 °C. FT-IR (neat) ν_max_ (cm^−1^): 3045, 1694, 1628, 1539, 1407, 1344. ^1^H NMR (400 MHz, CDCl_3_) *δ* (ppm) 2.48 (3H, s, CH_3_), 7.31 (1H, dd, *J* = 5.0, 3.8 Hz, H-3′), 7.39 (2H, d, *J* = 8.2 Hz, H-3′′, H-5′′), 7.50–7.58 (2H, m, H-8, H-9), 7.74 (1H, dd, *J* = 5.0, 1.2 Hz, H-4′), 7.99–8.04 (1H, m, H-10), 8.25 (2H, d, *J* = 8.4 Hz, H-2′′, H-6′′), 8.29–8.33 (1H, m, H-7), 8.77 (1H, dd, *J* = 3.8, 1.2 Hz, H-2′). ^13^C{^1^H} NMR (100 MHz, CDCl_3_) *δ* (ppm) 21.4, 113.0, 119.8 (2C), 120.8, 123.9, 125.4, 128.1, 128.6, 128.9, 130.3 (2C), 133.5, 134.0, 137.2, 140.0, 140.1, 144.1, 145.9, 149.7, 150.0. Anal. Calcd. for C_21_H_14_N_6_S: C 65.95, H 3.69, N 21.97; found: C 65.86, H 3.63, N 22.05.

2-(p-Tolyl)-4-(1-Methyl-1H-pyrrol-2-yl)-2H-benz[4,5]imidazo[1,2-a][1,2,3]triazolo[4,5-e]pyrimidine (**13d**): Yellow powder (0.22 g, yield 59%), m.p. > 300 °C. FT-IR (neat) ν_max_ (cm^−1^): 3080, 1626, 1548, 1437, 1405, 1219. ^1^H NMR (400 MHz, CDCl_3_) *δ* (ppm) 2.46 (3H, s, CH_3_), 4.36 (3H, s, NCH_3_), 6.36 (1H, dd, *J* = 4.0, 2.4 Hz, H-4′), 7,01 (1H, d, *J* = 2.4 Hz, H-3′), 7.36 (2H, d, *J* = 8.2 Hz, H-3′′, H-5′′), 7.45–7.54 (2H, m, H-8, H-9), 7.94 (1H, d, *J* = 7.5 Hz, H-10), 8.08 (1H, d, *J* = 1.7 Hz, H-5′), 8.21 (2H, d, *J* = 8.5 Hz, H-2′′, H-6′′), 8.25 (1H, d, *J* = 7.7 Hz, H-7). ^13^C{^1^H} NMR (100 MHz, CDCl_3_) *δ* (ppm) 21.3, 39.6, 109.9, 112.8, 119.7 (2C), 120.0, 122.2, 123.4, 125.2, 126.9, 128.0, 129.1, 130.2 (2C), 132.7, 137.3, 139.7, 143.5, 145.7, 148.0, 150.2. Anal. Calcd. for C_22_H_17_N_7_: C 69.64, H 4.52, N 25.84; found: C 69.53, H 4.59, N 25.76.

2-(p-Tolyl)-4-(1-naphthalene-2-ol)-2H-benz[4,5]imidazo[1,2-a][1,2,3]triazolo[4,5-e]pyrimidine (**13e**): Orange powder (0.29 g, yield 67%), m.p. > 300 °C. FT-IR (neat) ν_max_ (cm^−1^): 3041, 2951, 1623, 1433, 1339, 1274. ^1^H NMR (400 MHz, CDCl_3_) *δ* (ppm) 2.35 (3H, s, CH_3_), 7.20–7.39 (6H, m), 7.47–7.53 (2H, m, H-8, H-9), 7.77 (1H, d, *J* = 6.9 Hz, H-10), 7.88 (1H, d, *J* = 9.0 Hz, H-3′), 7.92–8.00 (3H, m), 8.24–8.32 (1H, m, H-7), 11.79 (1H, s, OH). ^13^C{^1^H} NMR (100 MHz, CDCl_3_) *δ* (ppm) 21.4, 112.3, 113.3, 119.8, 120.0 (2C), 120.8, 124.3, 124.4, 125.8, 126.2, 126.5, 128.0, 128.4, 129.1, 130.3, 130.4 (2C), 132.0, 135.1, 137.2, 140.3, 143.8, 146.2, 148.7, 158.7. Anal. Calcd. for C_27_H_18_N_6_O: C 73.29, H 4.10, N 18.99; found: C 73.35, H 4.16, N 18.82.

8,9-Difluor-2-(p-Tolyl)-4-(2,3,4-trimethoxyphenyl)-2H-benz[4,5]imidazo[1,2-a][1,2,3]triazolo[4,5-e]pyrimidine (**14a**): Yellow powder (0.32 g, yield 63%), m.p. 270–272 °C. FT-IR (neat) ν_max_ (cm^−1^): 2947, 1644, 1596, 1509, 1289, 1100. ^1^H NMR (400 MHz, CDCl_3_) *δ* (ppm) 2.47 (3H, s, CH_3_), 3.95 (3H, s, OCH_3_), 4.00 (3H, s, OCH_3_), 4.13 (3H, s, OCH_3_), 6.89 (1H, d, *J* = 8.7 Hz, H-5′), 7.38 (2H, d, *J* = 8.2 Hz, H-3′′, H-5′′), 7.76 (1H, d, *J* = 8.7 Hz, H-6′), 7.87 (1H, t, *J* = 8.7 Hz, H-10), 8.15–8.24 (3H, m, H-7, H-2′′, H-6′′). ^13^C{^1^H} NMR (100 MHz, CDCl_3_) *δ* (ppm) 21.4, 56.3, 61.0, 62.3, 101.3 (d, *J* = 23.4 Hz), 107.4, 108.3 (d, *J* = 20.4 Hz), 119.9 (2C), 122.5, 123.0 (d, *J* = 11.2 Hz), 127.0, 130.4 (2C), 130.7, 137.3, 139.7 (d, *J* = 11.2 Hz), 140.1, 142.7, 145.3, 148.7 (dd, *J* = 245.7, 14.7 Hz), 149.8 (dd, *J* = 243.0, 12.6 Hz), 151.4, 153.5, 156.3, 157.0. Anal. Calcd. for C_26_H_20_F_2_N_6_O_3_: C 62.15, H 4.01, N 16.73; found: C 62.27, H 4.16, N 16.59.

### 3.2. Biology

#### 3.2.1. Animals

All animal procedures were carried out under the generally accepted ethical standards for the manipulations on animals adopted by the European Convention for the Protection of Vertebrate Animals used for Experimental and Other Scientific Purposes (1986) and taking into account the International Recommendations of the European Convention for the Protection of Vertebrate Animals Used for Experimental Research (1997). The study was approved by the Local Ethics Committee of the Volgograd State Medical University (registration No. IRB 00005839 IORG 0004900, OHRP), Certificate No. 2021/056, 15 June 2021. All sections of this study adhere to the ARRIVE Guidelines for reporting animal research [21]. The experiments were carried out on 10 male Chinchilla rabbits weighing 3.0–3.5 kg and 95 outbred albino male rats weighing 250–270 g. Animals were kept under standard vivarium conditions (22–24 °C, 40–50% humidity, ambient light) during the study.

#### 3.2.2. In Vitro Anticoagulant Assay

The study was performed on platelet-poor plasma (PPP) stabilized with a 3.8% sodium citrate solution in a ratio of 9:1, according to the method in [22]. Dabigatran etexilate (Boehringer Ingelheim Pharma GmbH and Co., Ingelheim am Rhein, Germany) was used as a reference drug. Test compounds and the reference drug were evaluated at a concentration of 100 μM. Effect on rabbit blood coagulogram in vitro was determined chronometrically with a SOLAR hemocoagulometer (Belarus, Minsk) using commercial kits (Technology-Standard, Russia, Barnaul) as per the manufacturer’s instructions. The following parameters were determined: activated partial thromboplastin time, and thrombin and prothrombin time. Hypercytokinemia conditions were modeled by incubation of whole blood with *S. typhimurium* LPS (Sigma Aldrich, St. Louis, MO, USA) at a final concentration of 20 ng/mL and subsequent preparation of PPP. Compounds that showed high dose-dependent prolongation of thrombin time under conditions of LPS-treatment and without were assessed for IC_50_ values using the regression analysis method in the Microsoft Excel 2007 program (Microsoft Corporation, Albuquerque, NM, USA).

#### 3.2.3. Anticoagulant Assay in Animals

The most active compounds were studied in vivo on male rats after a single intragastric administration in a volume of no more than 2 mL. Distilled water was used as a vehicle. In all experiments, control animals were injected with a vehicle in an equivalent volume.

The reference drug dabigatran etexilate was administered to rats 2 h before the study at a 12 mg/kg dose (equivalent to the human dose, taking into account the interspecies conversion factor). The compounds were administered in doses equimolar to the dose of dabigatran etexilate, and the efficacy was assessed 1, 2, and 4 h after administration. Depending on the activity, the doses of the compounds under study were increased by two and four times.

Blood was taken from the inferior vena cava of rats anesthetized with 400 mg/kg chloral hydrate intraperitoneally. To stabilize the blood, a 3.8% aqueous solution of sodium citrate (pH 6.0) was used in a ratio of 9:1. Coagulogram parameters of platelet-poor plasma were measured with a SOLAR coagulometer according to the methods described above.

In addition, the assessment of the parameters of hemostasis in rats was carried out by the method of thromboelastography [23]. The following parameters were measured: *R*—time to formation of the first fibrin filaments; *K*—time from *R* until the clot reaches 20 mm; *α-Angle*—the tangent of the curve made as the *K* is reached; *MA*—maximum amplitude characterizing the functional activity of platelets and clot strength.

#### 3.2.4. Acute Toxicity

Acute toxicity of the most active compounds was determined on 10 white nonlinear male mice, weighing 20–22 g, with intraperitoneal administration. The deaths of animals were recorded within two weeks.

#### 3.2.5. Tail Bleeding Time (TBT) Model

These models are the most widely used approach for assessing hemostasis in mice. Bleeding time was determined on 10 white nonlinear male mice, weighing 20–22 g, with intragastrical administration. A scalpel was used to transect the tail at a predetermined distance from the tip (1–5 mm) [24]. The bleeding tail stump is immersed in normal saline warmed to 37 °C, and time to cessation of bleeding is determined.

#### 3.2.6. Statistical Analysis

Biological data were analyzed with 1-way ANOVA using Bonferroni’s multiple comparison correction using the Microsoft Excel 2007 spreadsheet editor, STATISTICA 5.0 (StatSoft, Inc., Palo Alto, CA, USA) for Windows, and Prism 5.0 (GraphPad Inc. San Diego, CA, USA). Data were presented as M + m, where m is SEM. Changes were statistically significant if *p* < 0.05. The calculation of ED50 (effective dose that prolongs thrombus formation time by 50%) was performed using linear regression analysis.

## 4. Conclusions

A series of 23 novel azolo[1,5-*a*]pyrimidine derivatives and their condensed analogs were evaluated in vitro for anticoagulant properties. We have identified five active compounds that significantly prolong thrombin time, outperforming the reference drug dabigatran etexilate. Antithrombin activity of most active compounds was confirmed using LPS-treated blood to mimic conditions of cytokine release syndrome. The studied compounds affected only the thrombin time value, reliably increasing it 6.5–15.2 times as compared to LPS-treated blood. IC_50_ values were determined for the two most active compounds **3a** and **3n** in the presence and in the absence of LPS. It was shown that the potency of compounds **3a** and **3n** exceeded dabigatran etexilate by 1.8 and 1.6 times, respectively, in normal blood, and by 1.2 times after LPS treatment. Most active compounds were also evaluated in animal experiments after a single intragastric administration to rats in doses equimolar to dabigatran etexilate. Compound **3a** did not change the parameters of the coagulogram 4 h after administration, while **3n** 4 h after administration prolonged the thrombin time by 1.4 times. Doubling the dose of compound 3a also failed to show detectable anticoagulant effects in vivo. Increased dose of compound 3n by two times had an antithrombin effect, increasing thrombin time by 3.3 times after 1, 2, and 4 h, which is comparable to the activity of dabigatran etexilate. After administration of 4 times increased dose of compound **3n**, we also observed an antithrombin effect comparable to the effect of dabigatran etexilate 1 h after intragastric administration. All the data obtained were confirmed by thromboelastography, which renders compound **3n** a promising anticoagulant agent. Compounds **3n** and **3m** in animal experiments were inactive, while compound 3n had a pronounced anticoagulant activity, but was inferior to the reference drug dabigatran etexilate. Due to the pronounced anticoagulant activity and low toxicity (<500 mg/kg), compound **3n** was chosen to study its effect on bleeding time prolongation and was studied in a bleeding time model. As a result of this study test, the compound was shown to prolong bleeding time insignificantly compared to the control group and to be comparable to the reference drug dabigatran etexilate.

Most active compounds were subjected to pharmacophore analysis by iterative neural network modeling. As a result, five types of QL descriptors significantly affecting the level of FIIa-inhibitory activity were identified, which correspond to neurons with Sens ≥ 1.1: {-N= … =O}, Sens = 1.22; {-N= … CycAr06}, Sens = 1.27; {-N= … CycAr05}, Sens = 1.23; {-N< … =O}, Sens = 1.10; and {-CH3 … >C(<)}, Sens = 1.10. The combination of these binding points forms a pharmacophore that provides a high level of FIIa-inhibitory activity of the tested compounds. The identified pharmacophore is also present in the structure of dabigatran, and almost all of the found pharmacophore fragments occur in its molecule several times.

## Figures and Tables

**Table 1:** Effect of derivatives of azolo[1,5-*a*]pyrimidines and their condensed analogs on the parameters of the coagulogram of rabbit blood in vitro at a concentration of 100 μM (M ± m, *n* = 5). APTT—activated partial thromboplastin time; TT—thrombin time; PT—prothrombin time.

No.	Compound	Coagulogram Parameter		
		APTT, Sec.	TT, Sec.	PT, Sec.
	Control	47.2 ± 0.3	11.7 ± 0.1	14.6 ± 0.1
1.	Dabigatran etexilate	79.6 ± 4.6 *	69.5 ± 4.5 *	16.7 ± 0.2
2.	Apixaban	137.5 ± 2.8 *^#^	14.6±0.1 *	67.8 ± 2.5 *^#^
3.	**3a**	65.3 ± 3.7	108.7 ± 9.9 *^#^	13.9 ± 0.4
4.	**3b**	63.7 ± 4.8	37.9 ± 1.6 *	10.8 ± 0.5
5.	**3c**	51.8 ± 1.3	29.1 ± 3.4 *	11.0 ± 0.2
6.	**3d**	58.8 ± 1.9	33.7 ± 2.1 *	11.6 ± 0.2
7.	**3e**	58.0 ± 1.9	30.3 ± 3.5 *	9.9 ± 0.1
8.	**3f**	50.8 ± 1.7	28.4 ± 1.3 *	10.1 ± 0.8
9.	**3g**	63.2 ± 1.8	30.1 ± 2.7 *	10.4 ± 0.4
10.	**3h**	50.4 ± 3.0	29.1 ± 0.6 *	10.1 ± 0.5
11.	**3j**	51.8 ± 1.4	31.0 ± 4.3 *	10.4 ± 0.7
12.	**3k**	50.6 ± 2.1 *	97.9 ± 19.1 *	13.9 ± 0.4
13.	**3l**	61.1 ± 4.1	28.5 ± 2.8 *	10.0 ± 0.6
14.	**3m**	63.9 ± 0.6	134.8 ± 10.4 *^#^	14.3 ± 0.6
15.	**3n**	55.5 ± 0.8	146.7 ± 5.2 *^#^	13.8 ± 0.1
16.	**3o**	52.9 ± 1.0	31.7 ± 3.9 *	10.9 ± 0.7
17.	**3p**	58.0 ± 4.0	34.8 ± 3.2 *	10.4 ± 0.3
18.	**3q**	59.5 ± 1.9	34.8 ± 3.3 *	10.6 ± 0.2
19.	**6a**	60.3 ± 2.8	30.7 ± 4.9 *	10.1 ± 0.4
20.	**6b**	52.4 ± 1.8	33.4 ± 5.5 *	10.5 ± 0.2
21.	**6c**	64.9 ± 2.7 *	64.5 ± 9.1 *	13.9 ± 0.2
22.	**9**	63.4 ± 4.0	53.2 ± 9.1 *	11.9 ± 0.6
23.	**13a**	62.7 ± 8.8	37.0 ± 4.0 *	10.8 ± 0.7
24.	**13b**	63.7 ± 2.0	35.6 ± 1.2 *	10.8 ± 0.5
25.	**13c**	53.1 ± 1.6	41.6 ± 1.9 *	10.3 ± 0.4
26.	**13d**	58.1 ± 1.0	40.5 ± 3.6 *	10.2 ± 0.1
27.	**13e**	51.8 ± 2.9	39.3 ± 1.7 *	11.5 ± 0.6
28.	**14a**	64.7 ± 5.8	40.0 ± 1.8 *	11.3 ± 0.9

Notes: *—(*p* ≤ 0.05) changes are statistically significant vs. control, 1-way ANOVA; ^#^—(*p* ≤ 0.05) changes are statistically significant vs. dabigatran etexilate, 1-way ANOVA.

**Table 2:** Neural networks were obtained after iterative modeling.

No. Iteration	Network Architecture	Correlation Coefficient		
		Training	Test	Validation
1	MLP126-9-1BFGS65ExpTanh	0.782	0.999	0.997
2	MLP102-10-1BFGS29ExpIdent	0.781	0.999	0.999
3	MLP 85-8-1BFGS23ExpTanh	0.781	0.999	0.999
4	MLP 71-6-1BFGS23ExpTanh	0.782	0.999	0.985
5	MLP 67-11-1BFGS23LogistTanh	0.782	0.999	0.829
6	MLP 66-11-1BFGS25TanhIdent	0.781	0.999	0.980
7	MLP 65-4-1BFGS56ExpTanh	0.782	0.999	0.983

Notes: MLP—multilayer perceptron; k-m-1—the number of input, hidden, and output neurons; BFGSN—algorithm for finding the minimum error function; Exp, Tanh, Ident, Logist—activation functions of the hidden and output layers of neurons, exponential, hyperbolic tangent, identical, logistic, respectively.

**Table 3:** Entry of the identified pharmacophore into structures of the most active FIIa inhibitors and dabigatran etexilate.

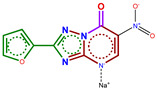	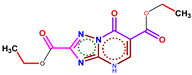	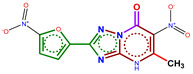
**3n**	**3a**	**3m**
{-N= … CycAr06}—1 {-N= … CycAr05}—4 {-N< … =O}—1 Total—6	{-N= … =O}—2 {-N= … CycAr06}—1 {-N= … CycAr05}—2 {-N< … =O}—2 {-CH3 … >C(<)}—2 Total—9	{-N= … CycAr06}—1 {-N= … CycAr05}—4 {-N< … =O}—1 {CH3 … >C(<)}—1 Total—7
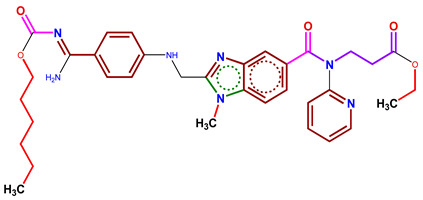		
Dabigatranetexilate		
{-N= … =O}—2 {-N= … CycAr06}—3 {-N= … CycAr05}—1 {-N< … =O}—3 {-CH3 … >C(<)}—9 Total—18		

**Table 4:** Effect of compounds on the parameters of coagulogram of rabbit blood treated with LPS in vitro in a concentration of 100 μM (M ± m, *n* = 5).

Compound	Parameters of Coagulogram		
	APTT, Sec.	TT, Sec.	PT, Sec.
Control	47.18 ± 0.34	11.65 ± 0.06	14.60 ± 0.10
LPS control	56.28 ± 1.60 *	13.131 ± 1.63	15.10 ± 0.07
Dabigatran etexilate	145.33 ± 25.17 *^#^	125.45 ± 1.54 *^#^	16.77 ± 0.20
Apixaban	156.3 ± 7.5 *^#^	15.5 ± 0.1 *	97.0 ± 5.3 *^#^
**3a**	65.73 ± 2.50 *	141.62 ± 12.55 *^#^	16.38 ± 0.35 *
**3k**	51.52 ± 2.26	116.9316 ± 16.79 *^#^	15.75 ± 0.74
**3m**	62.27 ± 1.07 *	85.05 ± 4.99 *^#^	15.75 ± 0.19
**3n**	61.43 ± 0.49 *	199.82 ± 4.39 *^#$^	15.20 ± 0.15

Notes: *—(*p* < 0.05) changes are statistically significant vs. control, 1-way ANOVA; ^#^—(*p* < 0.05) changes are statistically significant vs. LPS-control, 1-way ANOVA; ^$^—(*p* < 0.05) changes are statistically significant vs. dabigatran etexilate, 1-way ANOVA.

**Table 5:** IC_50_ of compounds **3a** and **3n** of thrombin time prolongation with and without LPS in vitro.

**Compound**	**∆% of Thrombin Time Prolongation Relative to Control**			**IC_50_, µM**
	**100 µM**	**10 µM**	**1 µM**	
Dabigatran etexilate	493.6 ± 4.5 *	302.0 ± 0.3 *	42.3 ± 0.6	24
**3a**	829.1 ± 10.0 *^$^	326.9 ± 1.5 *	40.6 ± 0.8	13
**3n**	1154.1 ± 5.2 *^$^	477.1 ± 3.4 *^$^	48.0 ± 1.3	15
**Compound**	**∆% of thrombin time prolongation relative to control + LPS**			**IC_50_, µM**
	**100 µM**	**10 µM**	**1 µM**	
Dabigatran etexilate + LPS	855.4 ± 1.5 ^#^	292.0 ± 0.6 ^#^	51.7 ± 0.5	11
**3a** + LPS	978.6 ± 12.6 ^#^	298.3 ± 4.2 ^#^	66.6 ± 1.2	91
**3n** + LPS	1421.8 ± 4.4 ^#$^	446.6 ± 6.2 ^#$^	70.6 ± 1.0	13

Notes: *—(*p* < 0.05) changes are statistically significant vs. control, 1-way ANOVA; ^#^—(*p* < 0.05) changes are statistically significant vs. LPS-control, 1-way ANOVA; ^$^—(*p* < 0.05) changes are statistically significant vs. dabigatranetexilate, 1-way ANOVA.

**Table 6:** Effect of compounds **3a** and **3n** in equimolar dabigatran etexilate doses on the coagulogram of rats with a single intragastric administration (M ± m, *n* = 5).

Sample	Dose, mg/kg	Time, h	Coagulogram Parameters		
			APTT, Sec.	TT, Sec.	PT, Sec.
Control			38.3 ± 1.7	57.7 ± 3.8	28.1 ± 1.4
Dabigatran etexilate	12.0 ^1^	2 ^#^	137.53 ± 2.79 *	637.4 ± 5.1 *	31.20 ± 1.17
**3a**	5.4 ^$^	1	32.2 ± 0.8	40.9 ± 6.0	24.9 ± 0.8
		2	37.2 ± 1.2	60.7 ± 6.0	26.4 ± 0.4
		4	33.3 ± 0.7	64.8 ± 3.4	27.7 ± 0.7
**3n**	5.8 ^$^	1	27.9 ± 2.3	68.6 ± 3.4	29.3 ± 1.3
		2	28.5 ± 1.3	65.5 ± 6.1	28.4 ± 1.6
		4	27.0 ± 2.7	78.5 ± 1.6 *	31.2 ± 1.2

Notes: ^1^—dose obtained by recalculation using the interspecies coefficient; ^$^—dose, equimolar to 12.0 mg/kg of dabigatran etexilate; *—*p* < 0.05) changes are statistically significant vs. control, 1-way ANOVA; ^#^—time to reach the maximum plasma concentration of dabigatran etexilate. *n*—number of experimental animals.

**Table 7:** Effect of compounds **3a** and **3n** on thromboelastogram (TEG) of rats with a single intragastric administration (M ± m, *n* = 5).

Sample	Dose, mg/kg	Time, h	TEG Parameters			
			*R*, min	*K*, min	*α*–*Angle*, Deg.	*MA*, RU
Control			6.4 ± 0.9	2.5 ± 0.1	61.5 ± 2.3	65.5 ± 1.2
Dabigatran etexilate	12.0 ^1^	2 ^#^	22.8 ± 3.3 *	16.8 ± 2.8 *	14.1 ± 3.1 *	37.1 ± 7.9 *
**3a**	5.4 ^$^	1	7.3 ± 0.6	2.9 ± 0.4	55.2 ± 3.5	68.2 ± 1.9
		2	6.9 ± 0.2	3.5 ± 0.3	51.7 ± 2.1	66.2 ± 0.3
		4	9.2 ± 1.0	2.7 ± 0.5	54.6 ± 6.1	66.2 ± 0.1
**3n**	5.8 ^$^	1	6.1 ± 1.6	2.4 ± 0.8	62.8 ± 7.6	66.1 ± 5.1
		2	5.4 ± 0.4	3.2 ± 0.5	54.3 ± 2.7	63.7 ± 2.5
		4	7.9 ± 0.5	11.6 ± 3.2 *	37.3 ± 7.8	46.9 ± 9.1

Notes: ^1^—dose obtained by recalculation using the interspecies coefficient; ^$^—dose, equimolar to 12.0 mg/kg of dabigatran etexilate; *—(*p* < 0.05) changes are statistically significant vs. control, 1-way ANOVA; ^#^—time to reach the maximum plasma concentration of dabigatran etexilate; *n*—number of experimental animals; *R*—time to formation of the first fibrin filaments; *K*—time from *R* until the clot reaches 20mm; *α*–*Angle*—the tangent of the curve made as the *K* is reached; *MA*—maximum amplitude characterizing the functional activity of platelets and clot strength.

**Table 8:** Effect of compounds **3a** and **3n** on the coagulogram of rats after a single intragastric administration at various intervals in increased doses (M ± m, *n* = 5 ).

Sample	Dose, mg/kg	Time, h	Coagulogram Parameters		
			APTT, Sec.	TT, Sec.	PT, Sec.
Control			38.3 ± 1.7	57.7 ± 3.8	28.1 ± 1.4
Dabigatran etexilate	12.0 ^1^	2 ^#^	140.1 ± 8.1 *	637.4 ± 5.1 *	30.5 ± 0.8
**3a**	10.8	1	35.9 ± 0.3	66.5 ± 11.0	29.1 ± 1.9
		2	35.8 ± 1.5	67.2 ± 5.6	25.8 ± 0.6
		4	35.4 ± 1.4	57.9 ± 6.4	26.2 ± 1.6
**3n**	11.6	1	37.0 ± 0.8	194.4 ± 19.8 *	25.4 ± 1.3
		2	32.3 ± 0.2	186.9 ± 2.5	22.9 ± 4.4
		4	36.1 ± 1.2	198.3 ± 26.7 *	28.7 ± 1.4
	23.2	1	31.6 ± 1.5	655.6 ± 2.1 *	29.3 ± 0.6
		2	31.7 ± 0.7	116.5 ± 13.7 *	20.9 ± 0.3
		4	34.4 ± 0.7	95.6 ± 25.8	21.8 ± 0.9

Notes: ^1^—dose obtained by recalculation using the interspecies coefficient; *—(*p* < 0.05) changes are statistically significant vs. control, 1-way ANOVA; ^#^—time to reach the maximum plasma concentration of dabigatran etexilate; *n*—number of experimental animals.

**Table 9:** Effect of compounds **3a** and **3n** on the TEG of rats after a single intragastric administration at various intervals in an increased doses (M ± m, *n* = 5).

Sample	Dose, mg/kg	Time, h	TEG Parameters			
			*R*, min	*K*, min	*α*–*Angle*, Deg.	*MA*, RU
Control			6.4 ± 0.9	2.5 ± 0.1	61.5 ± 2.3	65.5 ± 1.2
Dabigatran etexilate	12.0 ^1^	2 ^#^	22.8 ± 3.3 *	16.8 ± 2.8 *	14.1 ± 3.1 *	37.1±7.9 *
**3a**	10.8	1	8.1 ± 0.6	2.3 ± 0.1	60.3 ± 0.1	69.6 ± 2.3
		2	5.9 ± 0.4	1.8 ± 0.3	67.3 ± 0.4	69.5 ± 3.3
		4	5.9 ± 0.6	2.3 ± 0.5	61.1 ± 3.1	73.3 ± 2.3
**3n**	11.6	1	6.2 ± 1.1	4.6 ± 1.1	41.5 ± 7.6	52.8 ± 2.5
		2	8.7 ± 0.3	6.2 ± 0.4 *	32.3 ± 1.5 *	50.3 ± 1.3
		4	11.9 ± 1.0 *	8.9 ± 0.9 *	24.5 ± 1.2 *	46.7 ± 1.6 *
	23.2	1	13.6 ± 1.5 *	9.2 ± 0.9 *	29.0 ± 2.8 *	45.1 ± 3.6 *
		2	9.0 ± 0.01 *	5.0 ± 1.8	37.4 ± 6.7 *	53.5 ± 3.6
		4	20.1 ± 8.4	18.1 ± 7.3	14.9 ± 6.4 *	34.5 ± 2.9 *

Notes: ^1^—dose obtained by recalculation using the interspecies coefficient; *—(*p* < 0.05) changes are statistically significant vs. control, 1-way ANOVA; ^#^—time to reach the maximum plasma concentration of dabigatran etexilate; *n*—number of experimental animals.

**Table 10:** Bleeding times in mice.

Sample	Dose, mg/kg	TBT, s
Control		288.3 ± 29.7
Dabigatran etexilate	12.0	268.5 ± 17.0
**3n**	23.2	313.0 ± 11.4

## Data Availability

Data are contained within article.

## Supplementary Materials

The following are available online, Figures NMR and IR Spectra of compounds **3**, **6**, **9**, **13**, and **14**.

## Sample Availability

Samples of the compounds **3–14** are available from the authors.
