# Supplementary material for: Azolo[1,5-a]pyrimidines and Their Condensed Analogs with Anticoagulant Activity
Source: Molecules. 2022 Jan 2;27(1):274. doi: 10.3390/molecules27010274 (PMC8746358; doi:10.3390/molecules27010274)
Supplement: Supplementary file 1 [file molecules-27-00274-s001.zip › molecules-1515006-supplementary.pdf]

Supporting Information

AZOLO[1,5-a]PYRIMIDINES AND THEIR CONDENSED ANALOGS WITH ANTICOAGULANT ACTIVITY

Konstantin V. Savateev[a], Victor V. Fedotov[a], Vladimir L. Rusinov[a], Svetlana K. Kotovskaya[a], Alexandr A. Spasov[b], Aida F. Kucheryavenko[b], Pavel M. Vasiliev[b], Vadim A. Kosolapov[b], Victor S. Sirotenko[b], Kseniya A. Gaidukova[b], Georgiy M. Uskov[b]*

[a] Department of organic and biomolecular chemistry, Ural Federal University named after the First President of Russia B.N. Eltsin, Mira st. 19, Yekaterinburg, 620002, Russian Federation; E-mail: i-krafttt@yandex.ru

[b] Department of Pharmacology and Bioinformatics, Volgograd State Medical University, Pavshikh Bortsov Sq. 1, Volgograd, 400131, Russian Federation

Table of contents

Spectra of compounds...2

Spectra of compounds

2,6-Diethoxycarbonyl-1,2,4-triazolo[1,5-*a*]pyrimidin-7-one (3a)

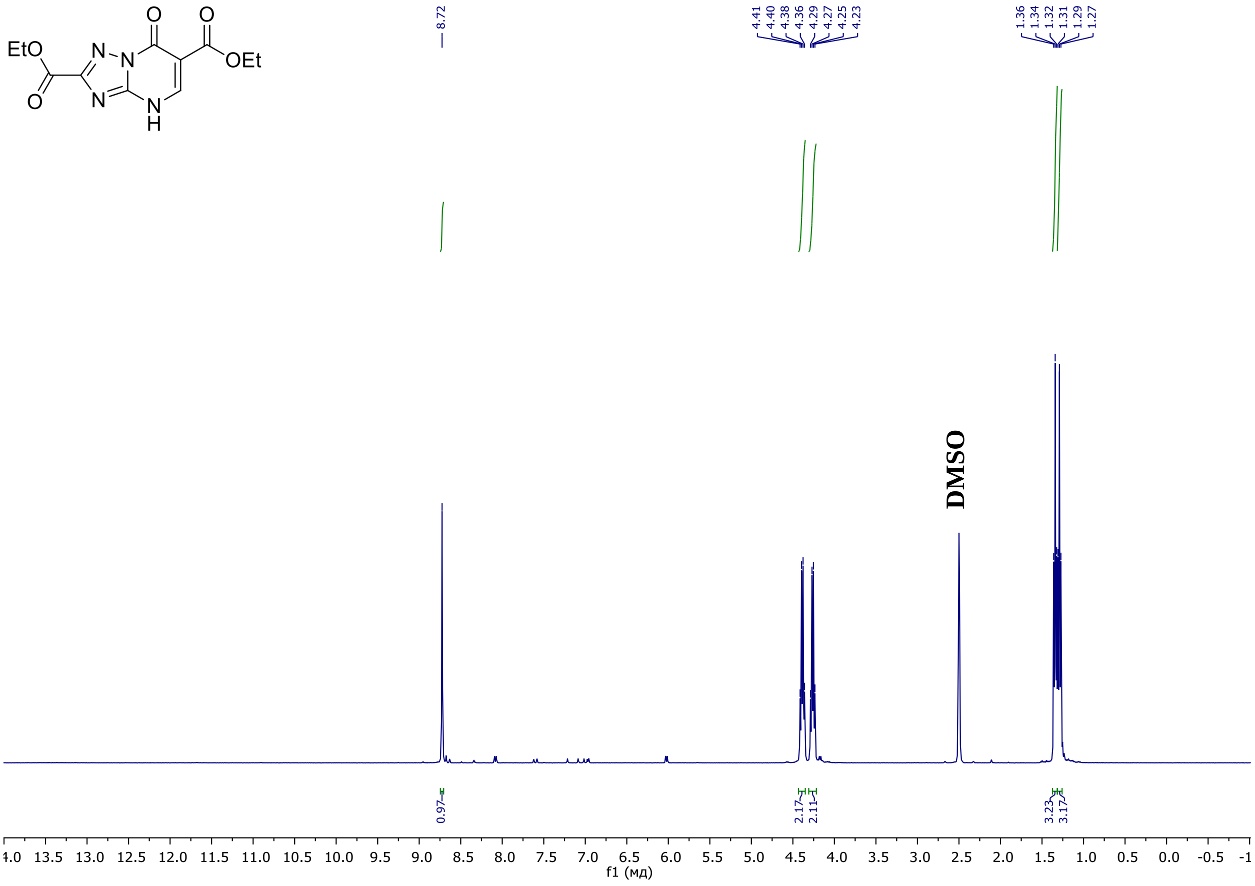

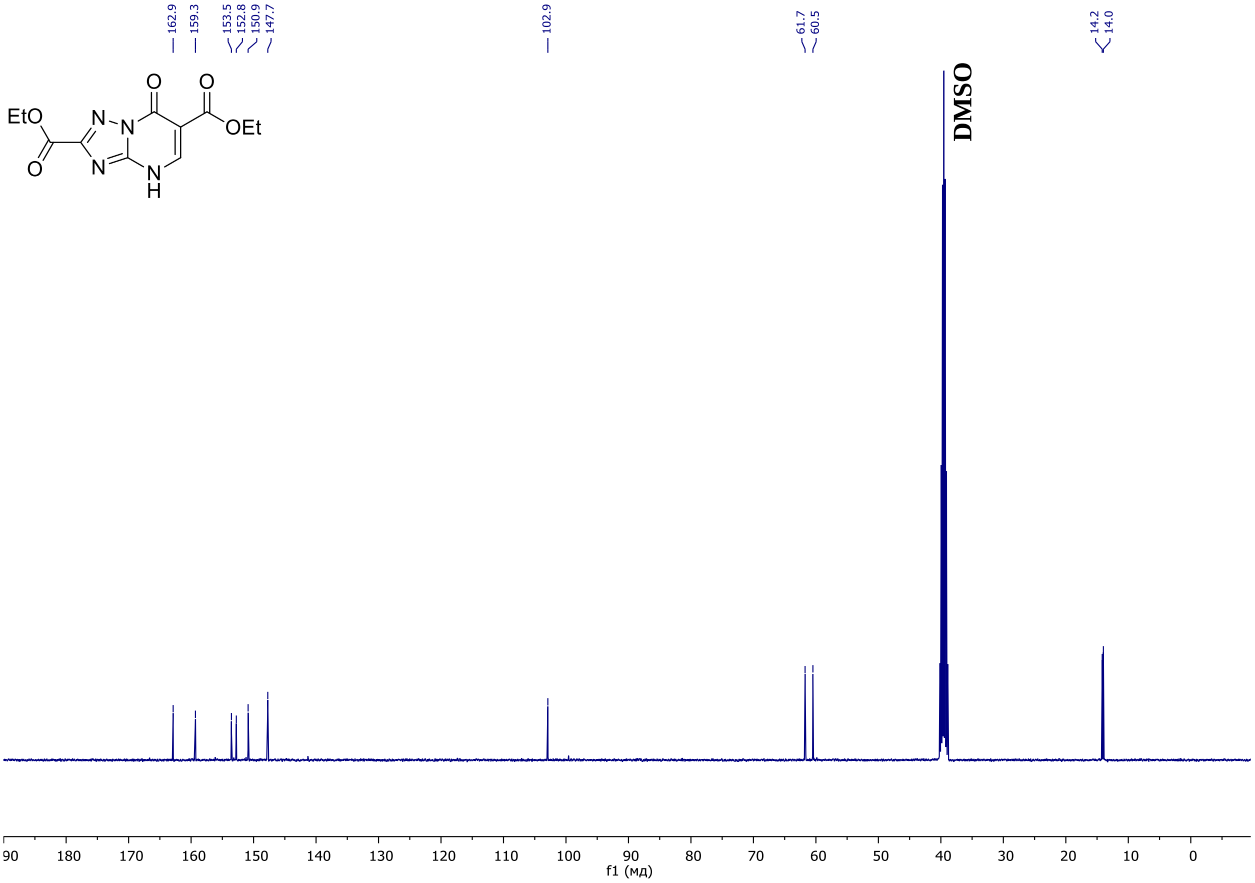

Figure S1. ^{1}H NMR (400 MHz, DMSO-d_6) and ^{13}C NMR (100 MHz, DMSO-d_6) spectra of **3a**

2,6-Diethoxycarbonyl-1,2,4-triazolo[1,5-*a*]pyrimidin-7-one (3a)

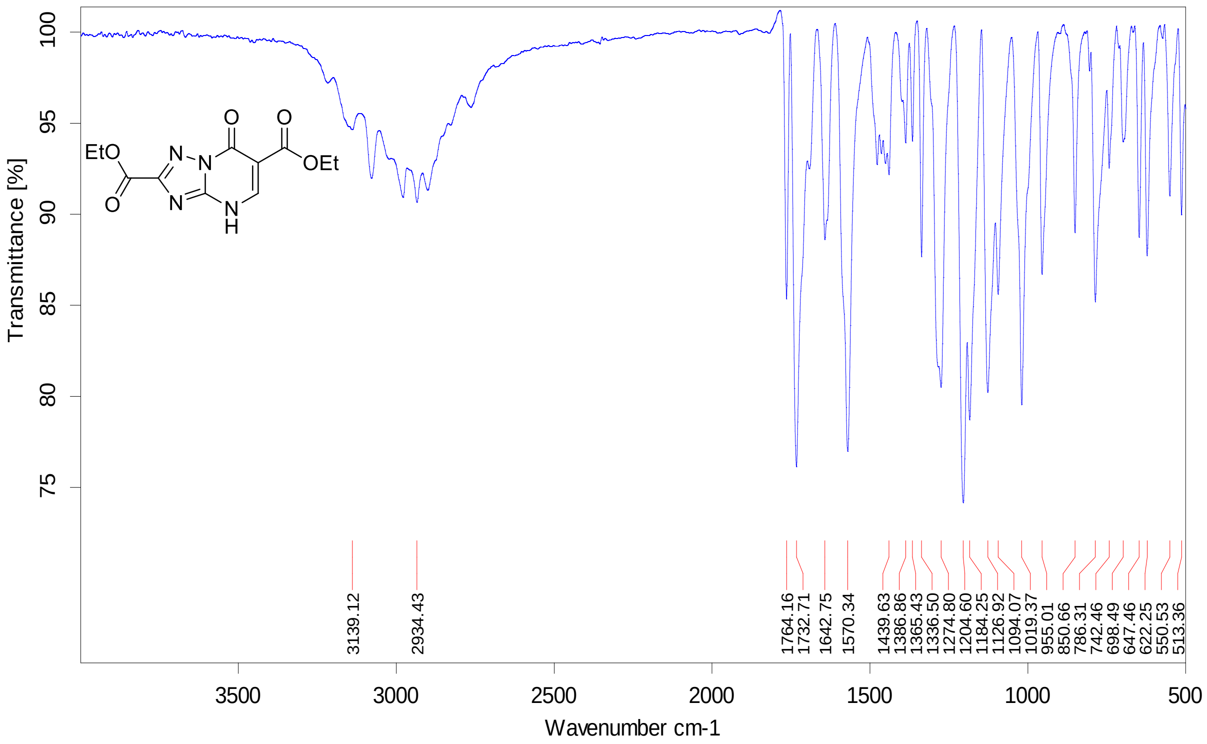

Figure S2. IR spectra of **3a**

2-(Thien-2-yl)-6-ethoxycarbonyl-1,2,4-triazolo[1,5-*a*]pyrimidin-7-one (3f)

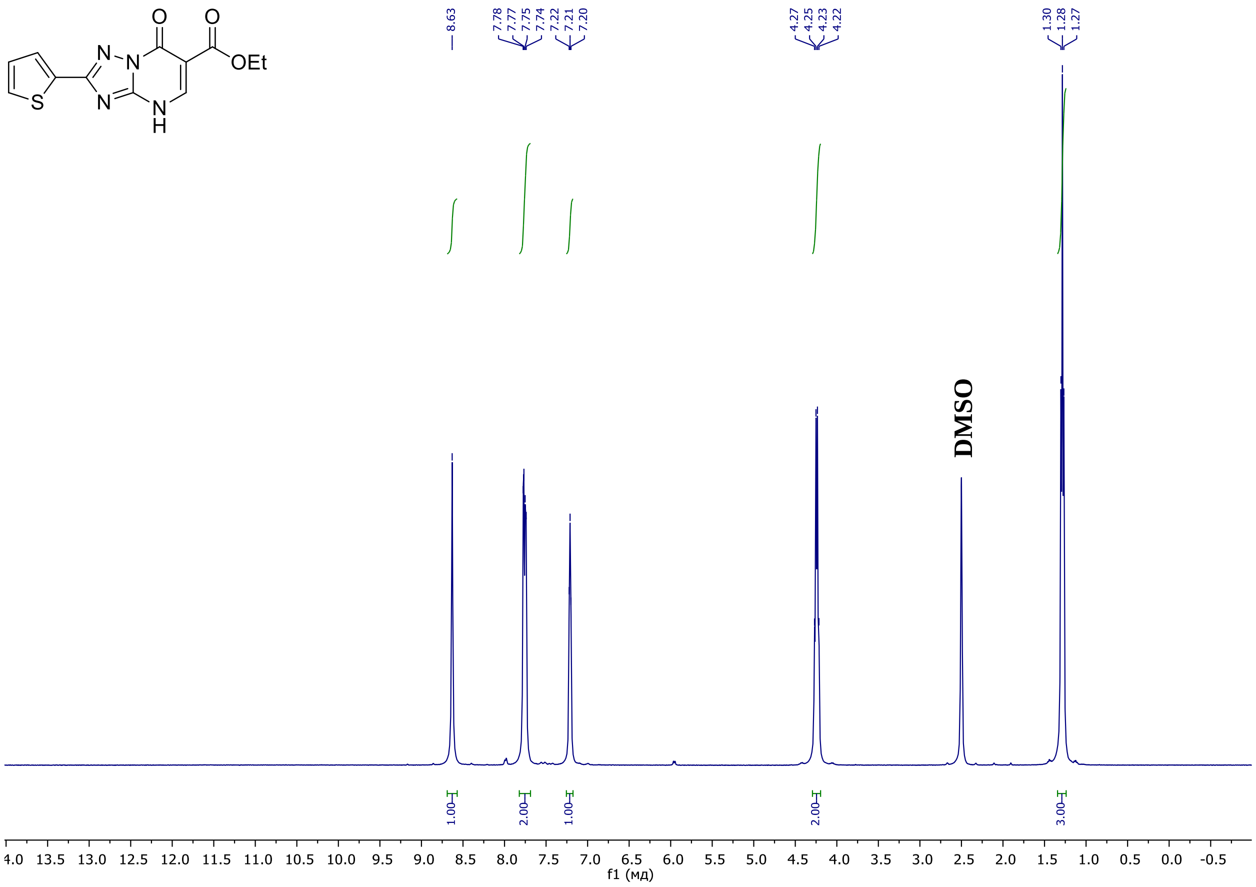

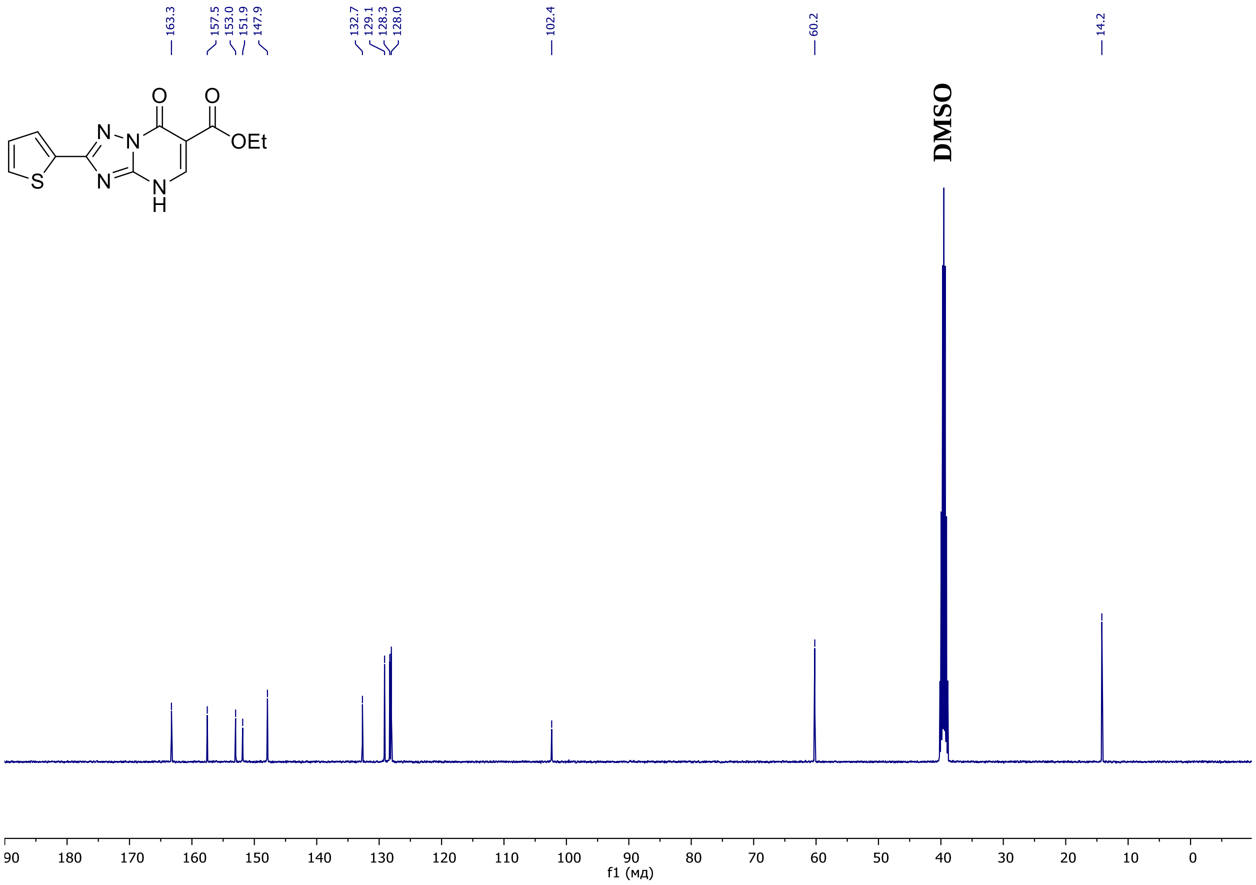

Figure S3. ^{1}H NMR (400 MHz, DMSO-d_6) and ^{13}C NMR (100 MHz, DMSO-d_6) spectra of **3f**

2-(Thien-2-yl)-6-ethoxycarbonyl-1,2,4-triazolo[1,5-*a*]pyrimidin-7-one (3f)

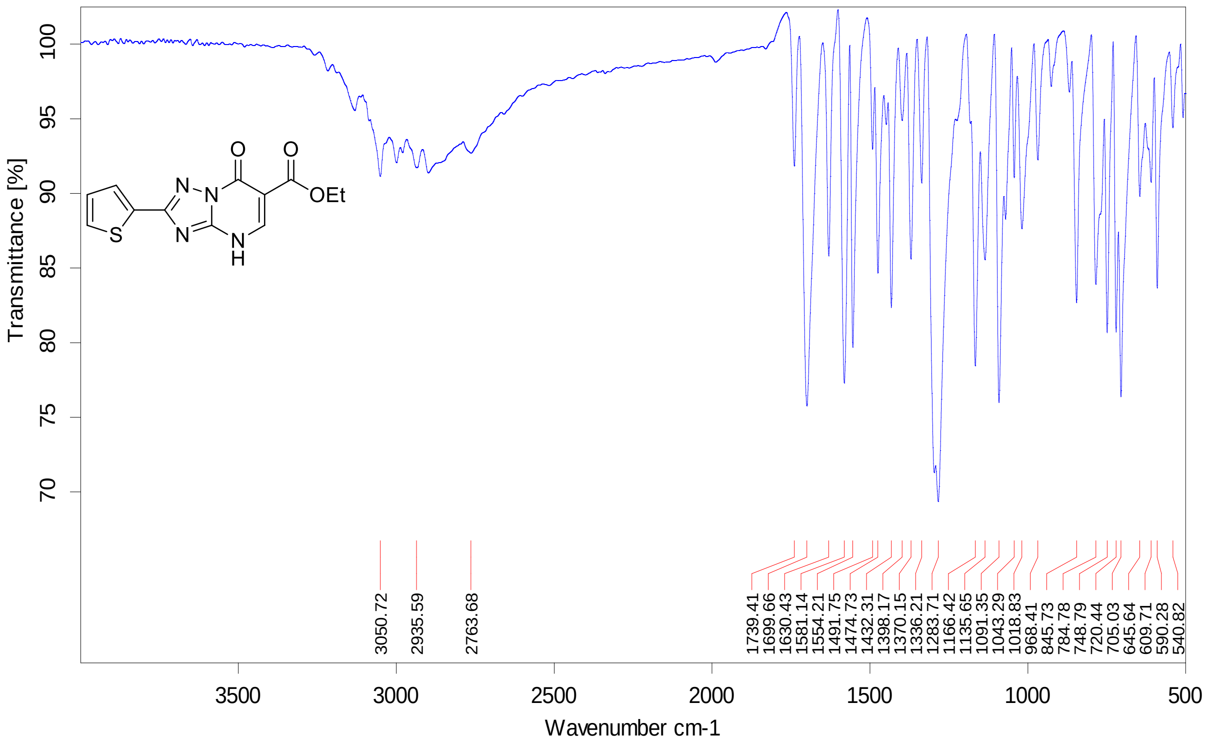

Figure S4. IR spectra of **3f**

2-(Pyridin-3-yl)-6-nitro-1,2,4-triazolo[1,5-*a*]pyrimidin-7-one (3g)

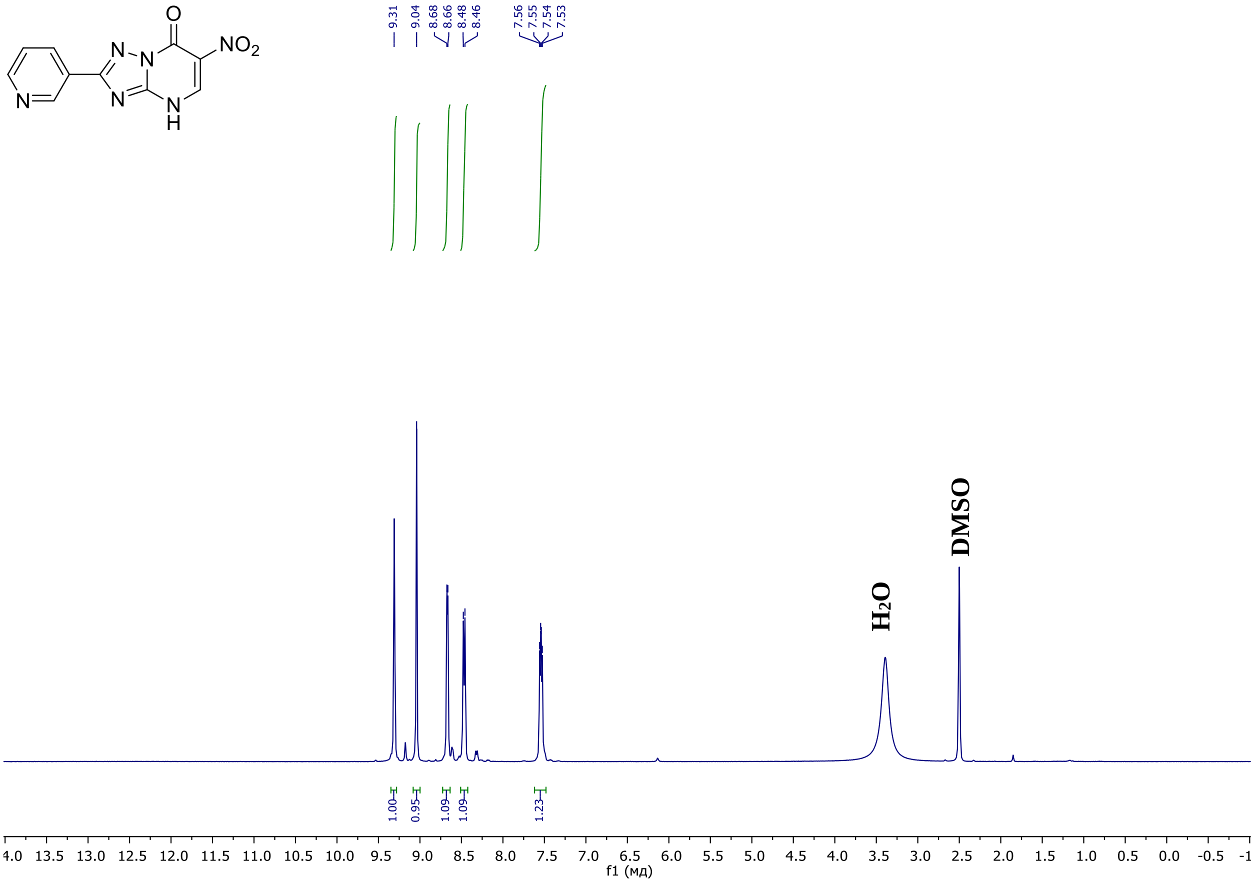

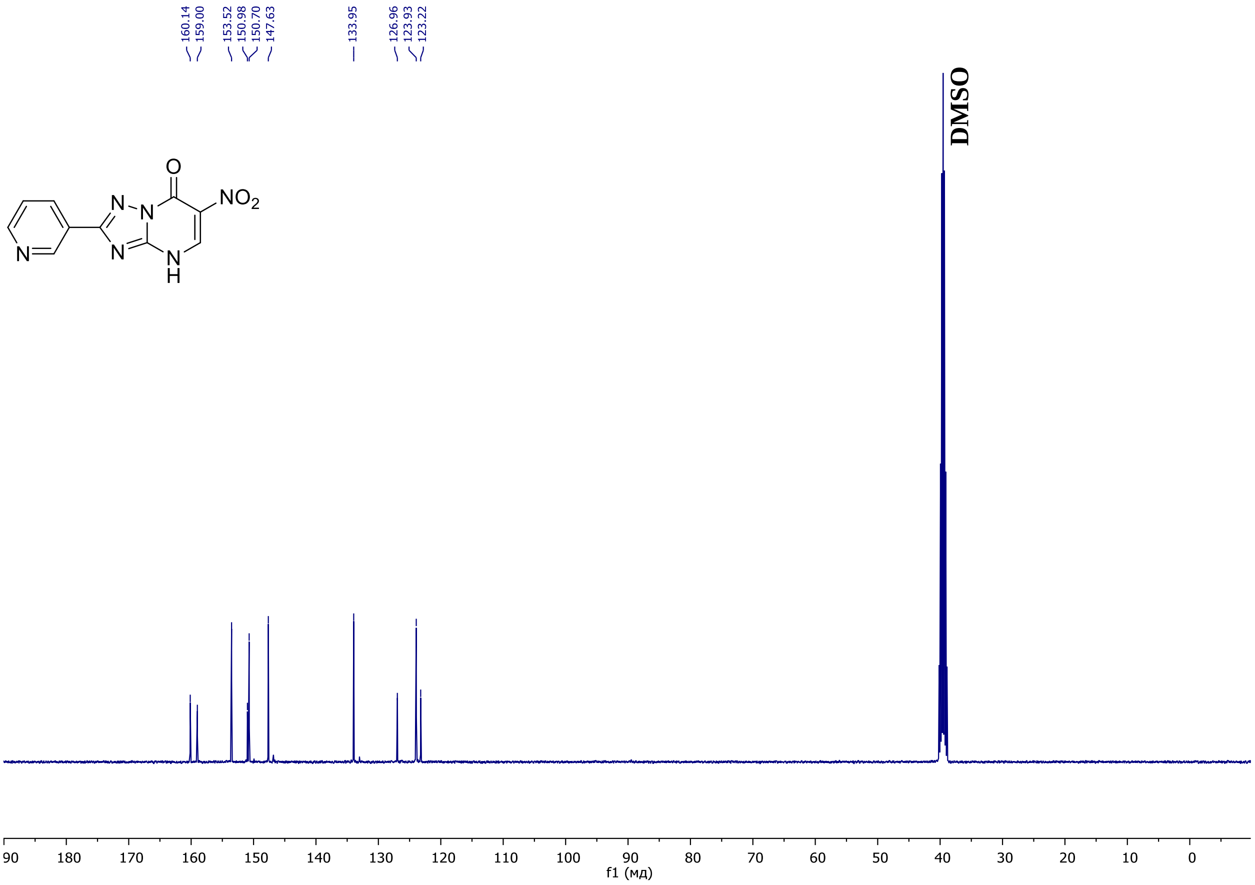

Figure S5. ^{1}H NMR (400 MHz, DMSO-d_6) and ^{13}C NMR (100 MHz, DMSO-d_6) spectra of **3g**

2-(Pyridin-3-yl)-6-nitro-1,2,4-triazolo[1,5-*a*]pyrimidin-7-one (3g)

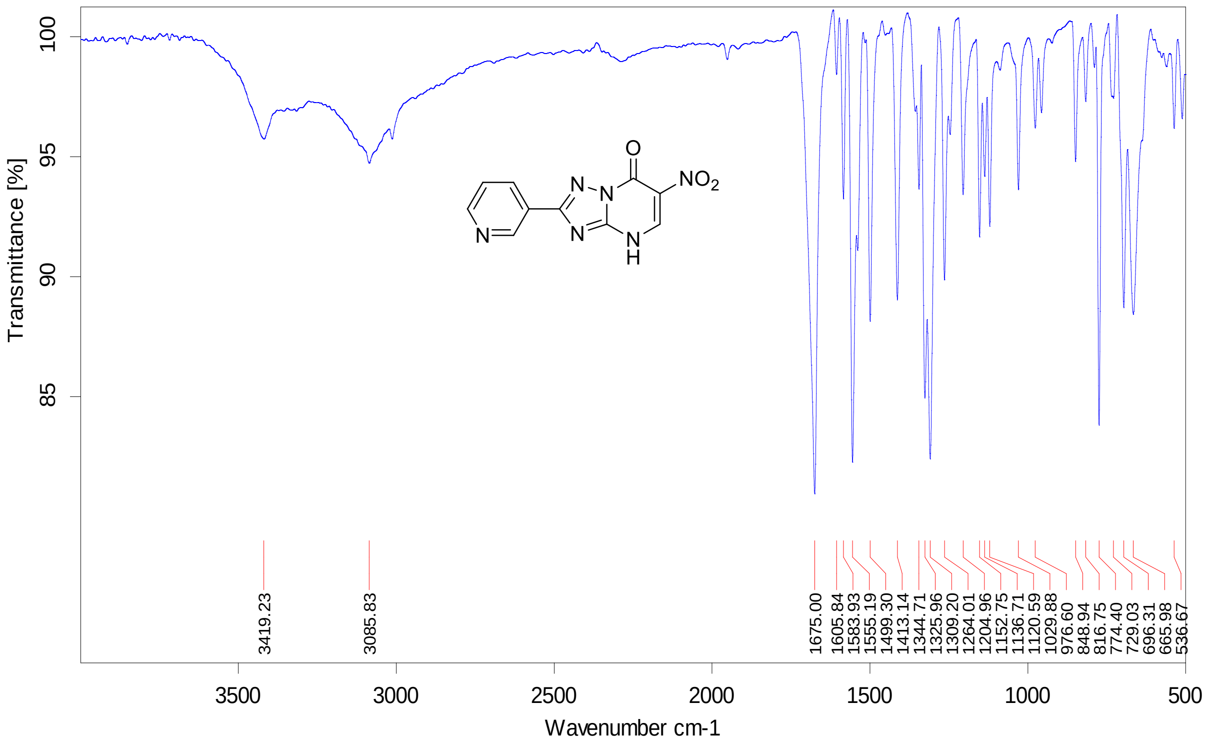

Figure S6. IR spectra of **3g**

2-phenyl-6-nitro-1,2,4-triazolo[1,5-*a*]pyrimidin-7-one pyridinium (3h)

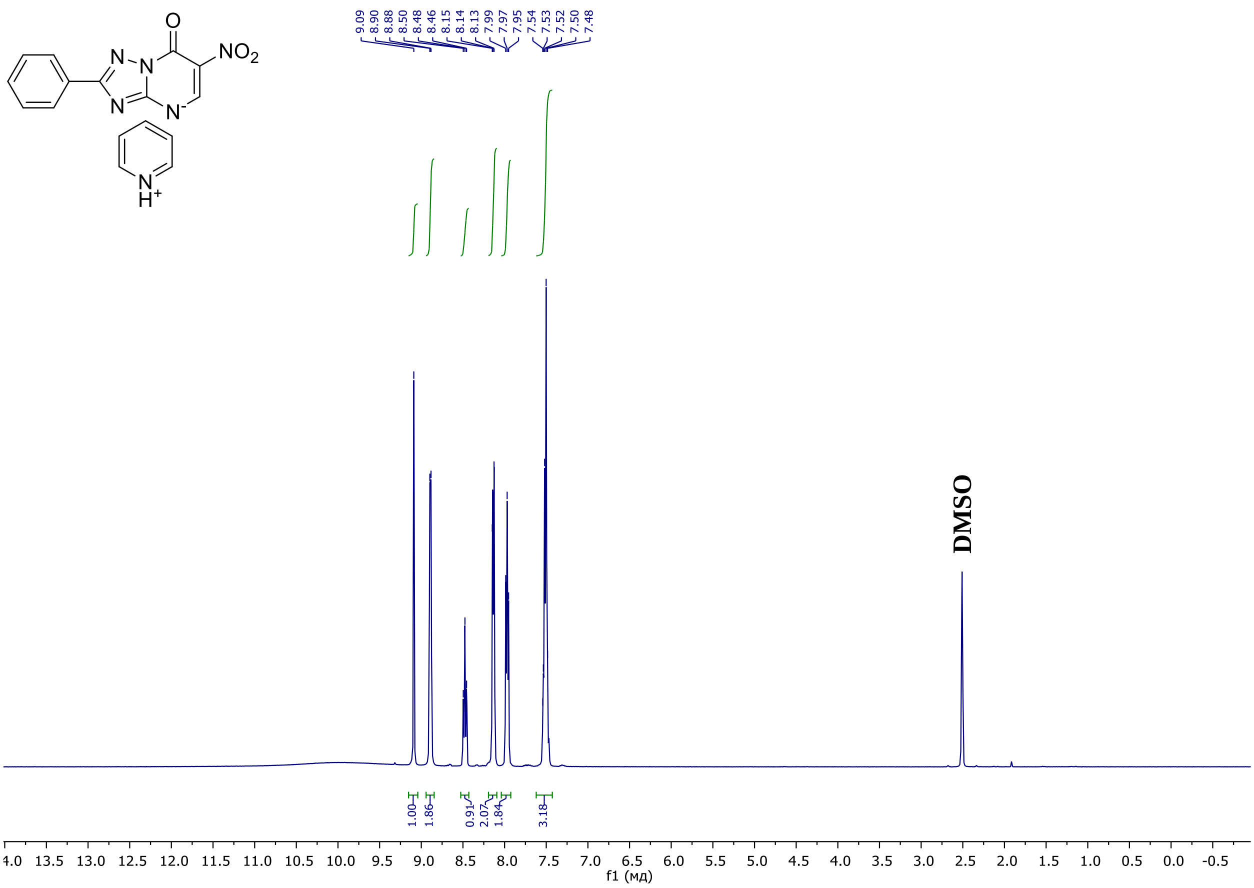

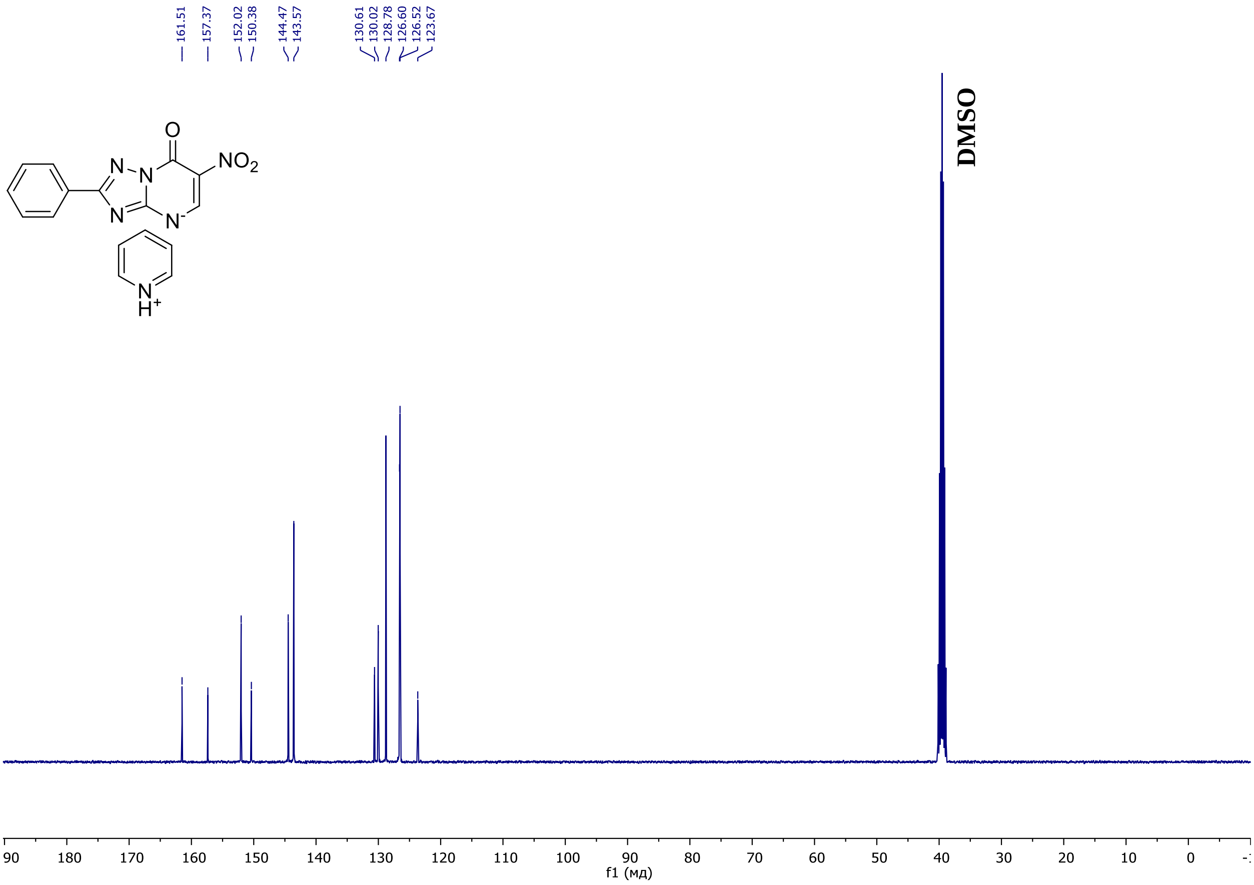

Figure S7. ^{1}H NMR (400 MHz, DMSO-d_6) and ^{13}C NMR (100 MHz, DMSO-d_6) spectra of **3h**

2-phenyl-6-nitro-1,2,4-triazolo[1,5-*a*]pyrimidin-7-one pyridinium (3h)

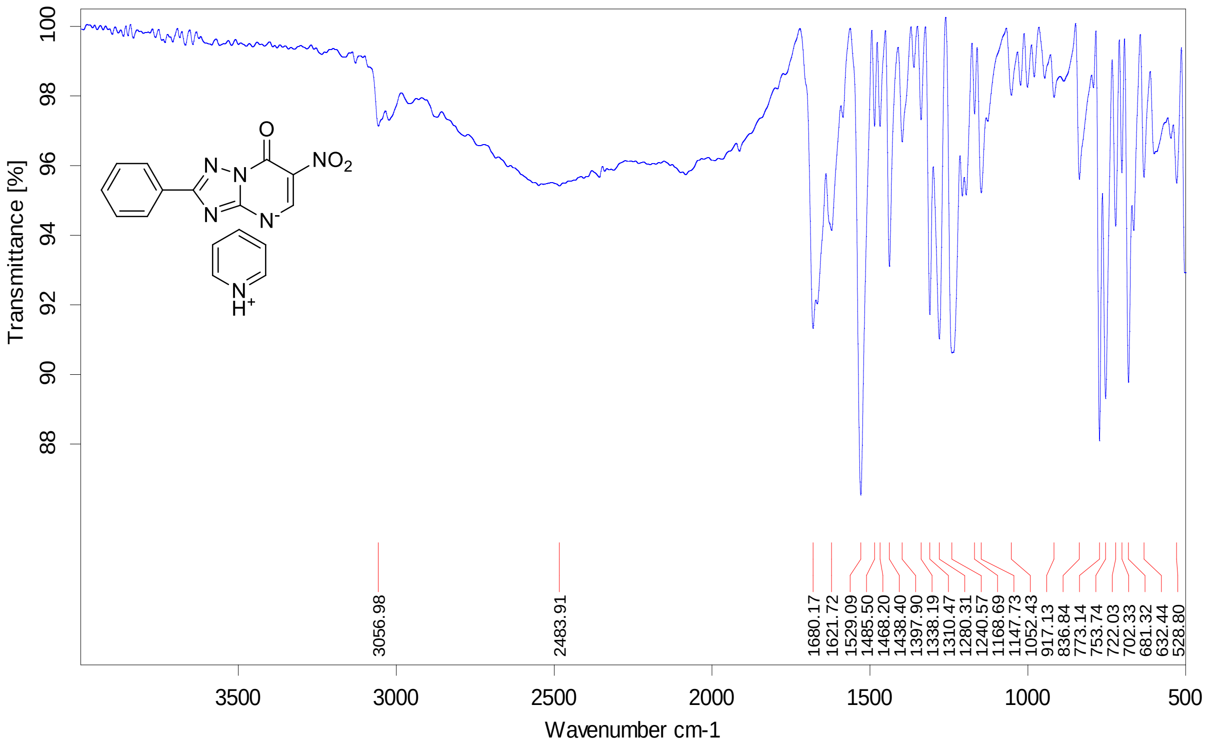

Figure S8. IR spectra of **3h**

2-(fur-2-yl)-6-nitro-1,2,4-triazolo[1,5-*a*]pyrimidin-7-one pyridinium (3i)

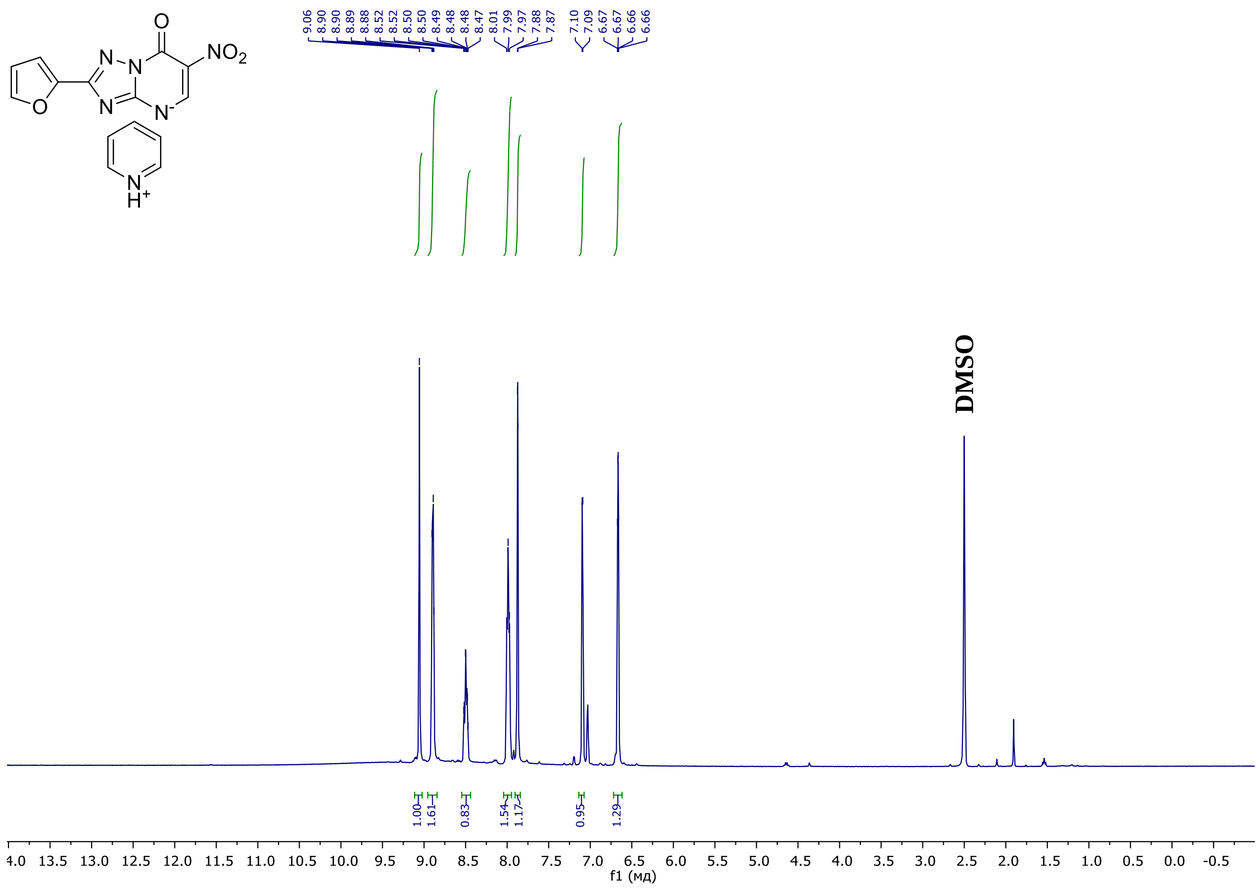

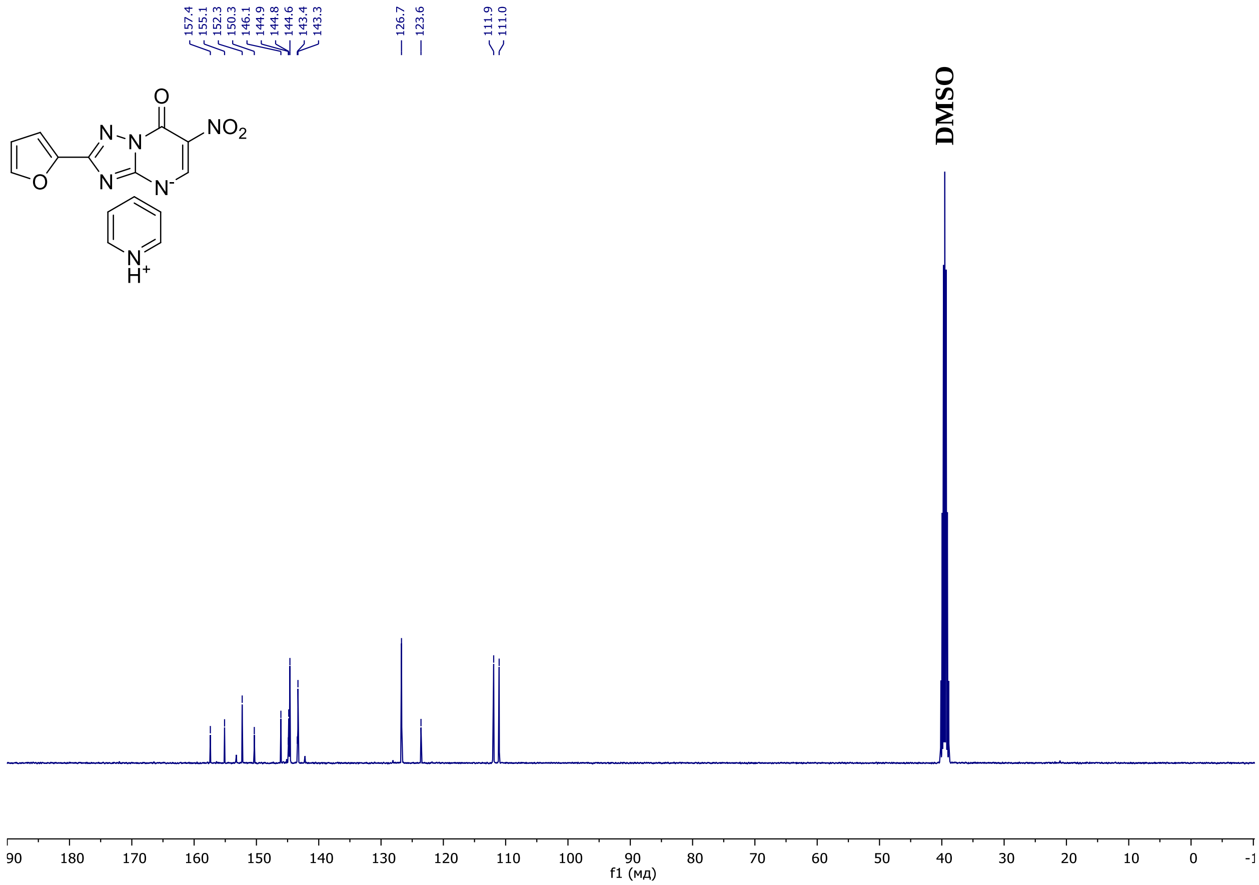

Figure S9. ^{1}H NMR (400 MHz, DMSO-d_6) and ^{13}C NMR (100 MHz, DMSO-d_6) spectra of **3i**

2-(fur-2-yl)-6-nitro-1,2,4-triazolo[1,5-*a*]pyrimidin-7-one pyridinium (3i)

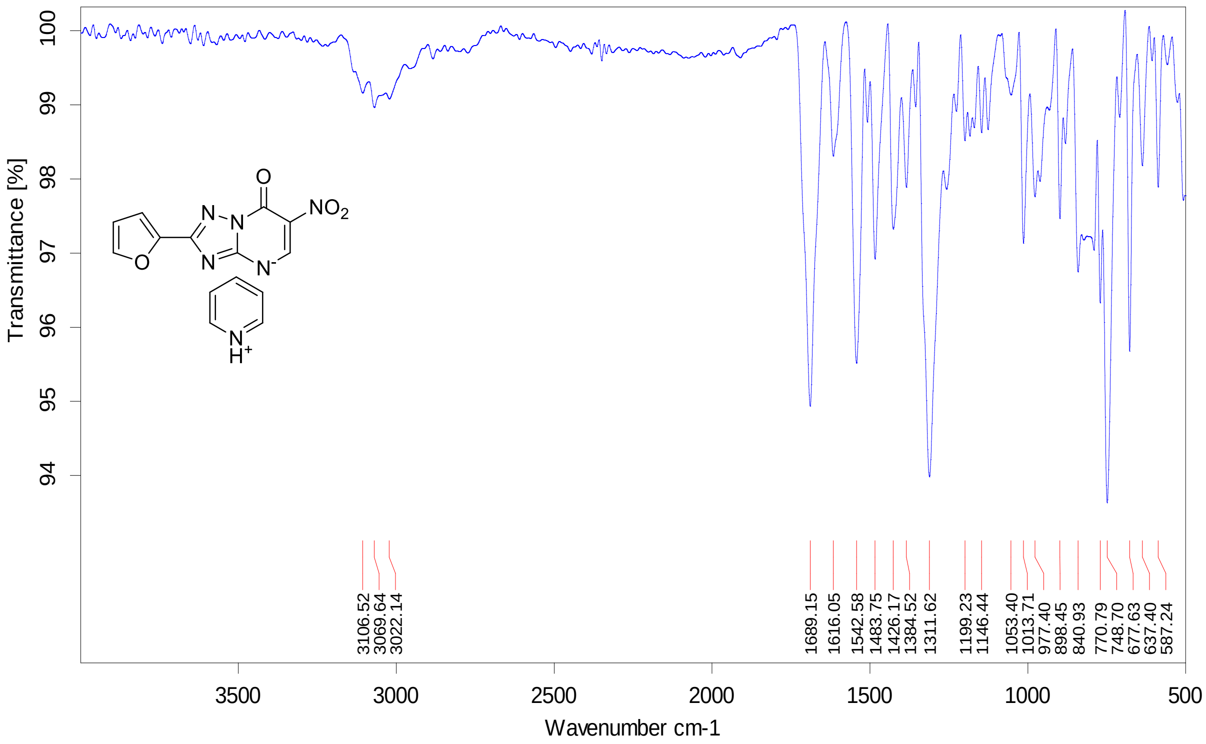

Figure S10. IR spectra of **3i**

2-(thien-2-yl)-6-nitro-1,2,4-triazolo[1,5-*a*]pyrimidin-7-one pyridinium (3j)

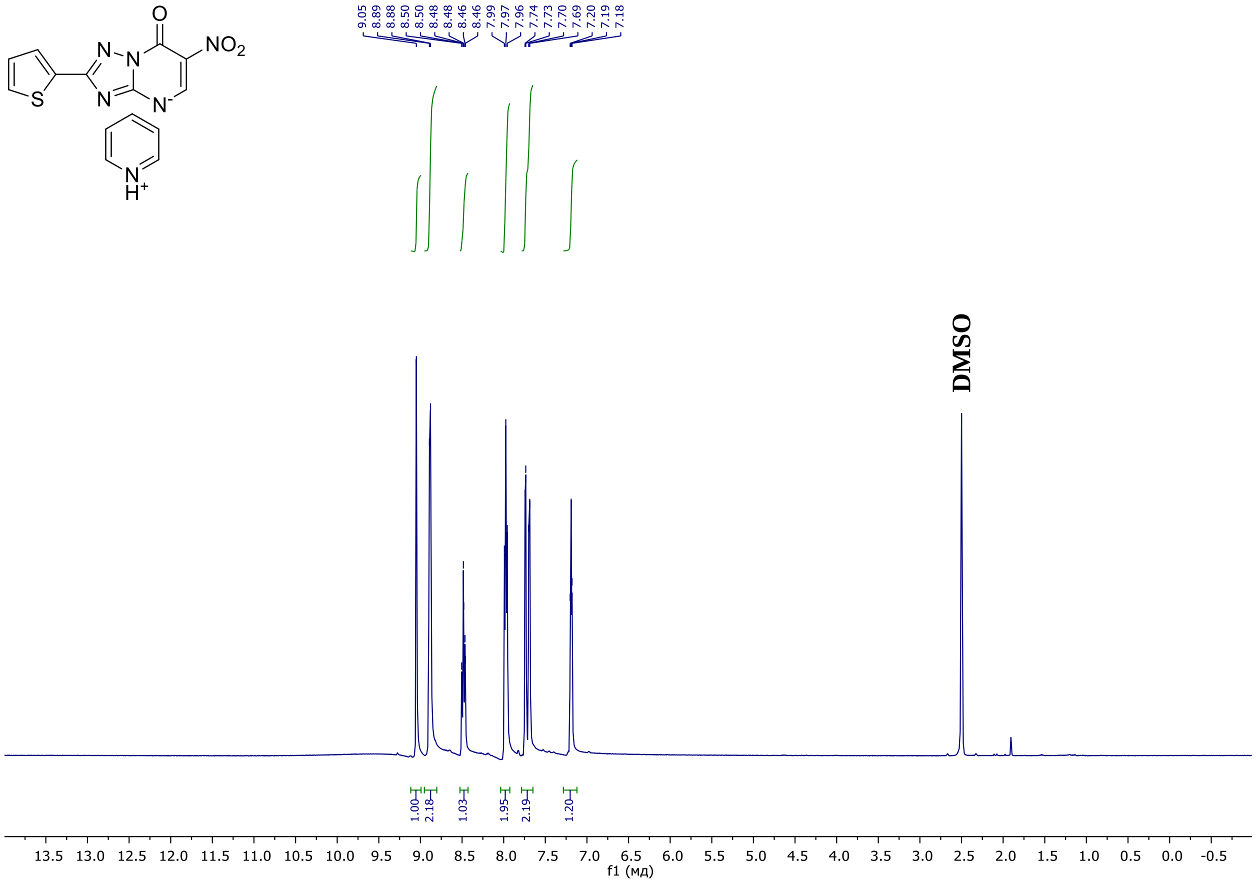

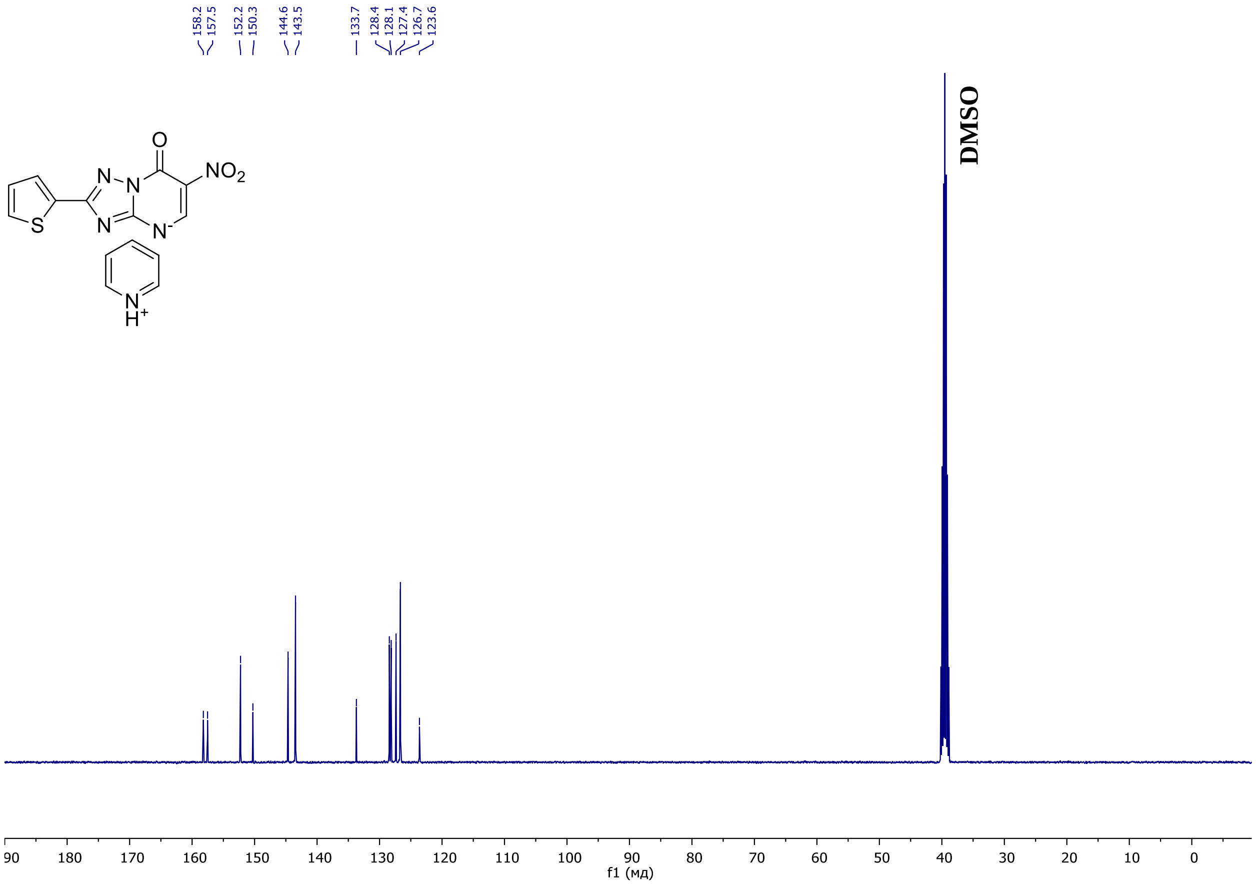

Figure S11. ^{1}H NMR (400 MHz, DMSO-d_6) and ^{13}C NMR (100 MHz, DMSO-d_6) spectra of **3j**

2-(thien-2-yl)-6-nitro-1,2,4-triazolo[1,5-*a*]pyrimidin-7-one pyridinium (3j)

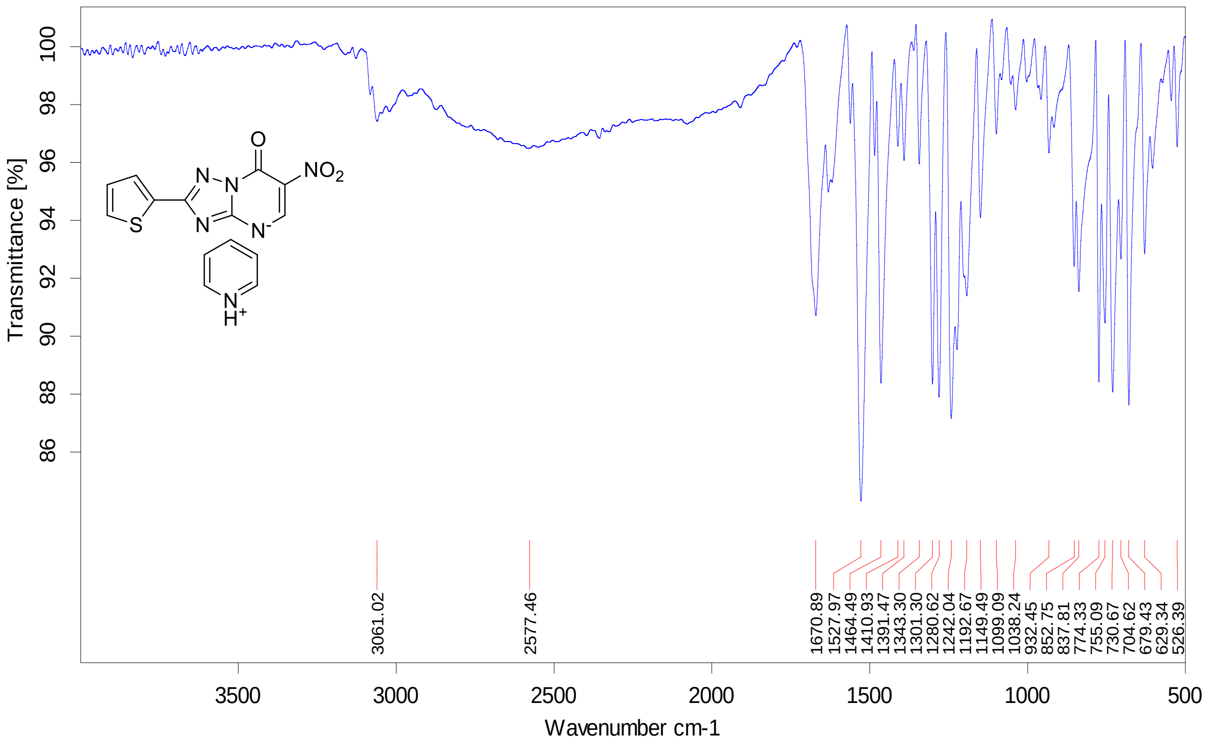

Figure S12. IR spectra of **3j**

2-(5-nitrofur-2-yl)-6-nitro-1,2,4-triazolo[1,5-*a*]pyrimidin-7-one pyridinium (3k)

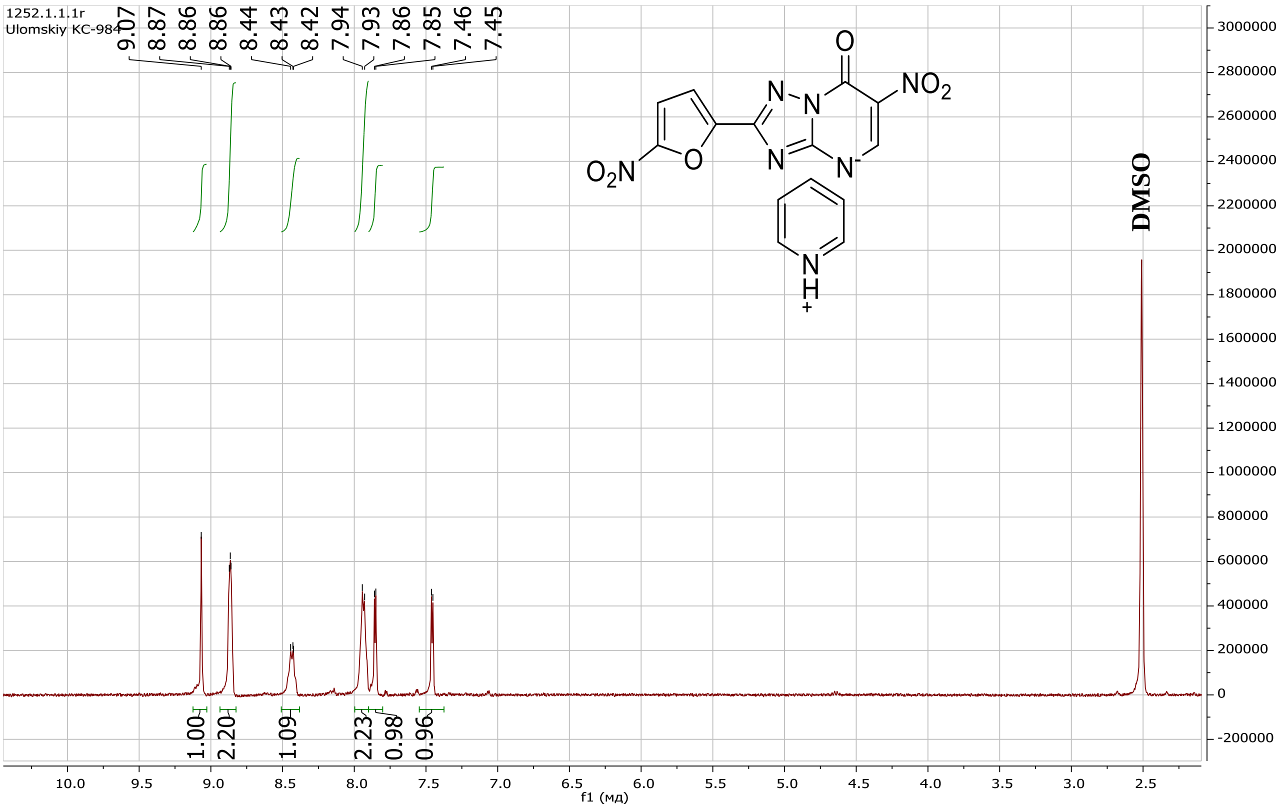

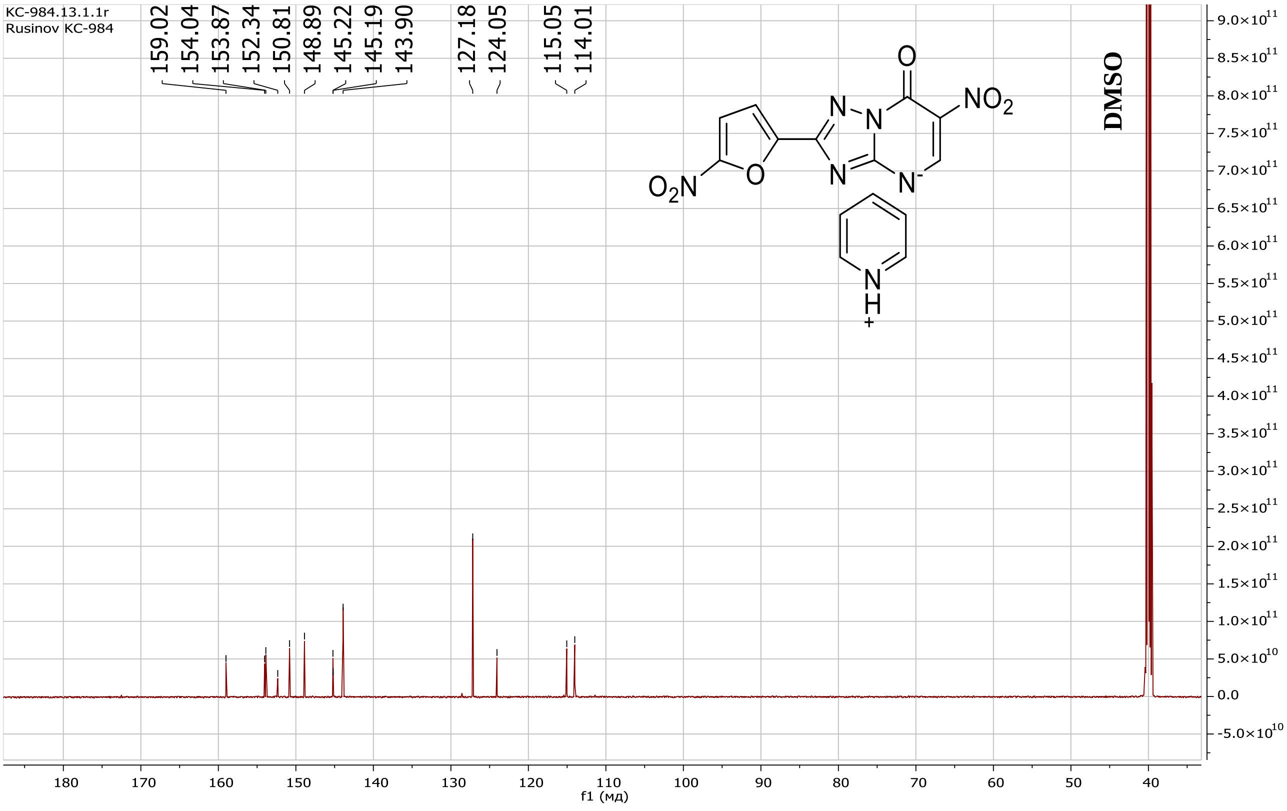

Figure S13. ^{1}H NMR (400 MHz, DMSO-d_6) and ^{13}C NMR (100 MHz, DMSO-d_6) spectra of **3k**

2-(5-nitrofur-2-yl)-6-nitro-1,2,4-triazolo[1,5-*a*]pyrimidin-7-one pyridinium (3k)

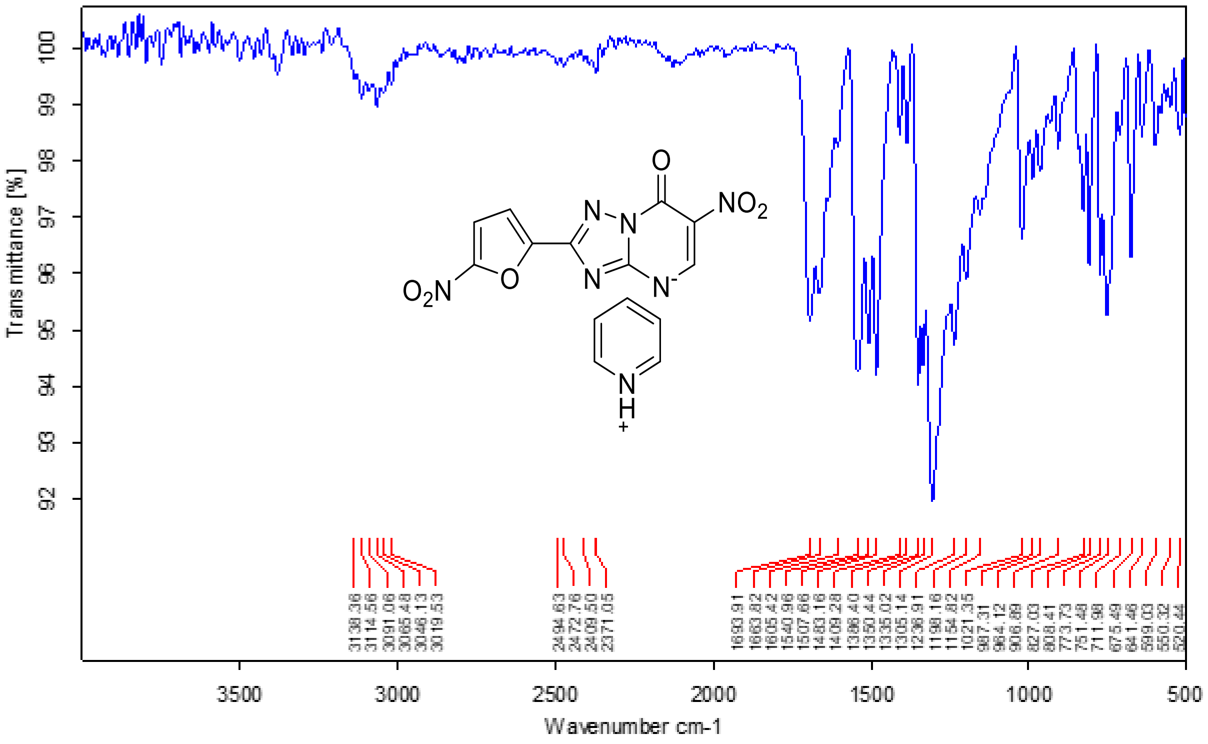

Figure S14. IR spectra of **3k**

2-phenyl-3-cyano-6-nitropyrazolo[1,5-*a*]pyrimidin-7-one pyridinium (3l)

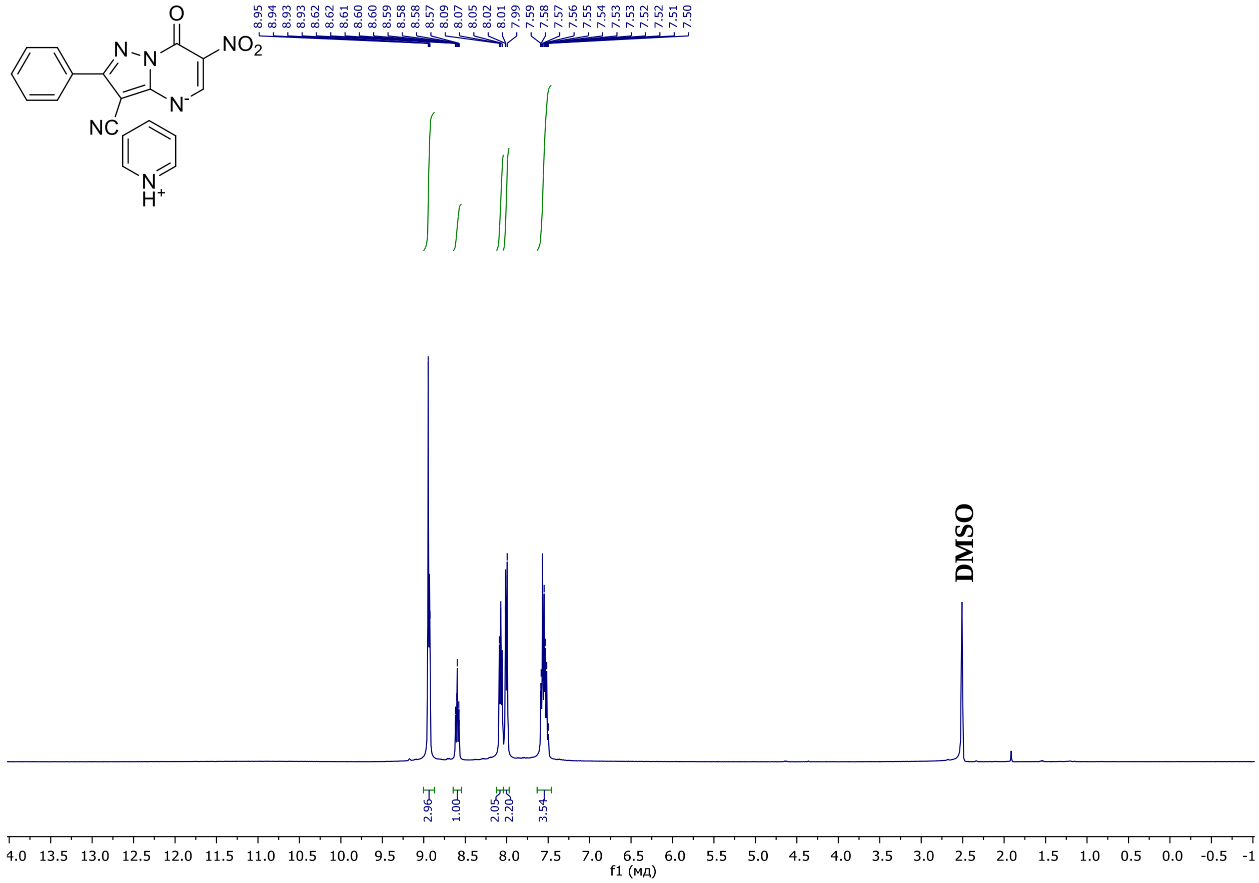

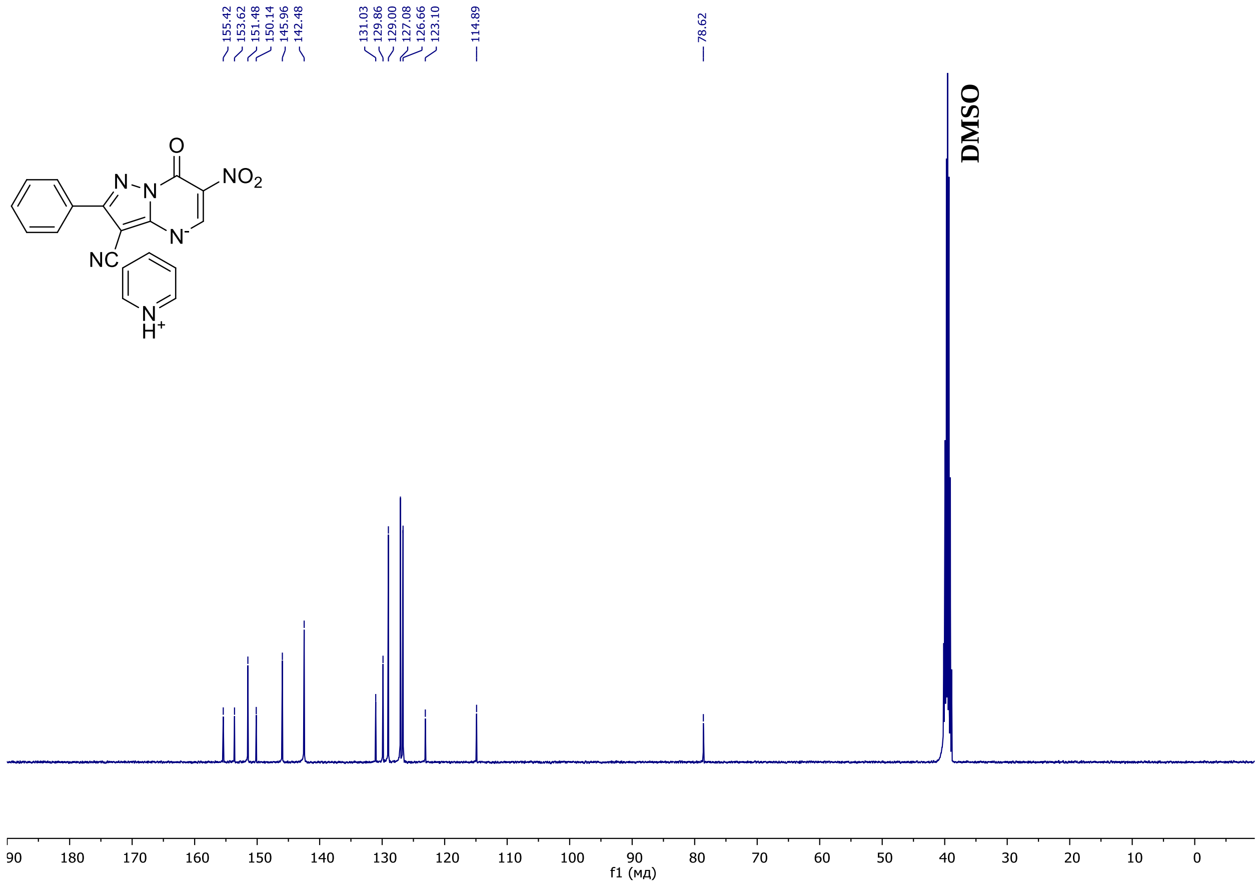

Figure S15. ^{1}H NMR (400 MHz, DMSO-d_6) and ^{13}C NMR (100 MHz, DMSO-d_6) spectra of **3l**

2-phenyl-3-cyano-6-nitropyrazolo[1,5-*a*]pyrimidin-7-one pyridinium (3l)

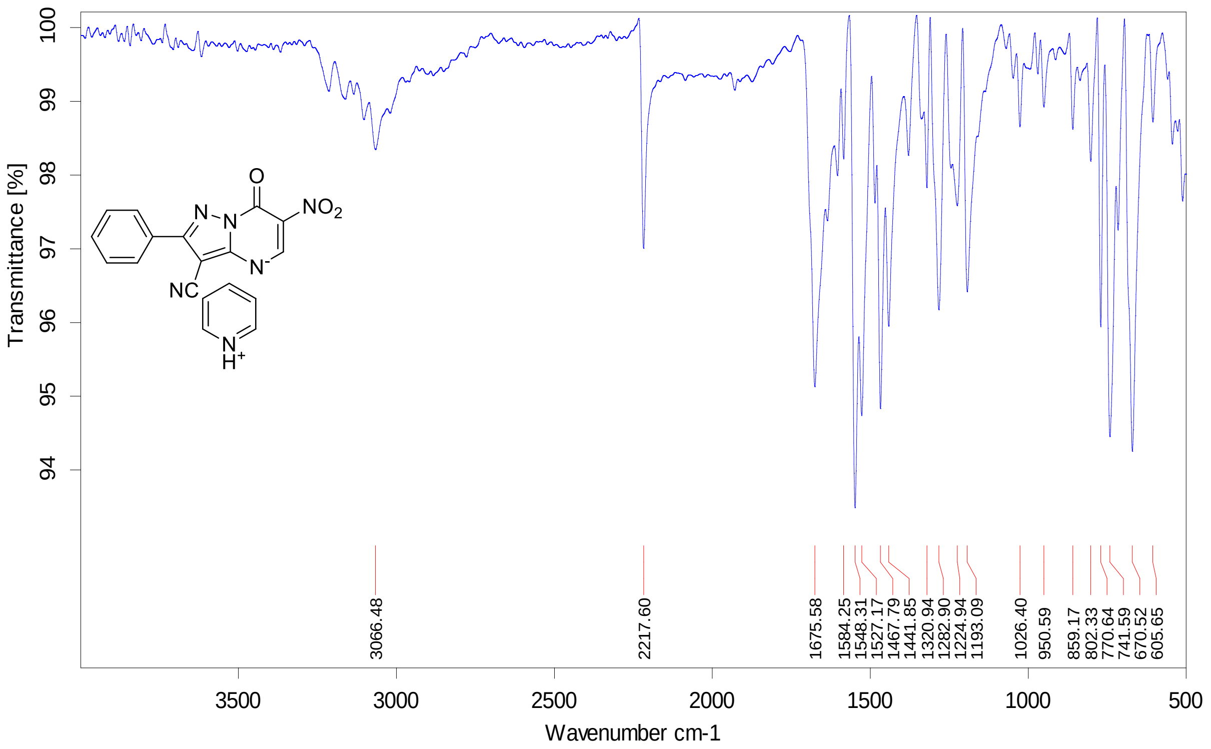

Figure S16. IR spectra of **3l**

2-(5-nitrofur-2-yl)-5-methyl-6-nitro-1,2,4-triazolo[1,5-*a*]pyrimidin-7-one (3m)

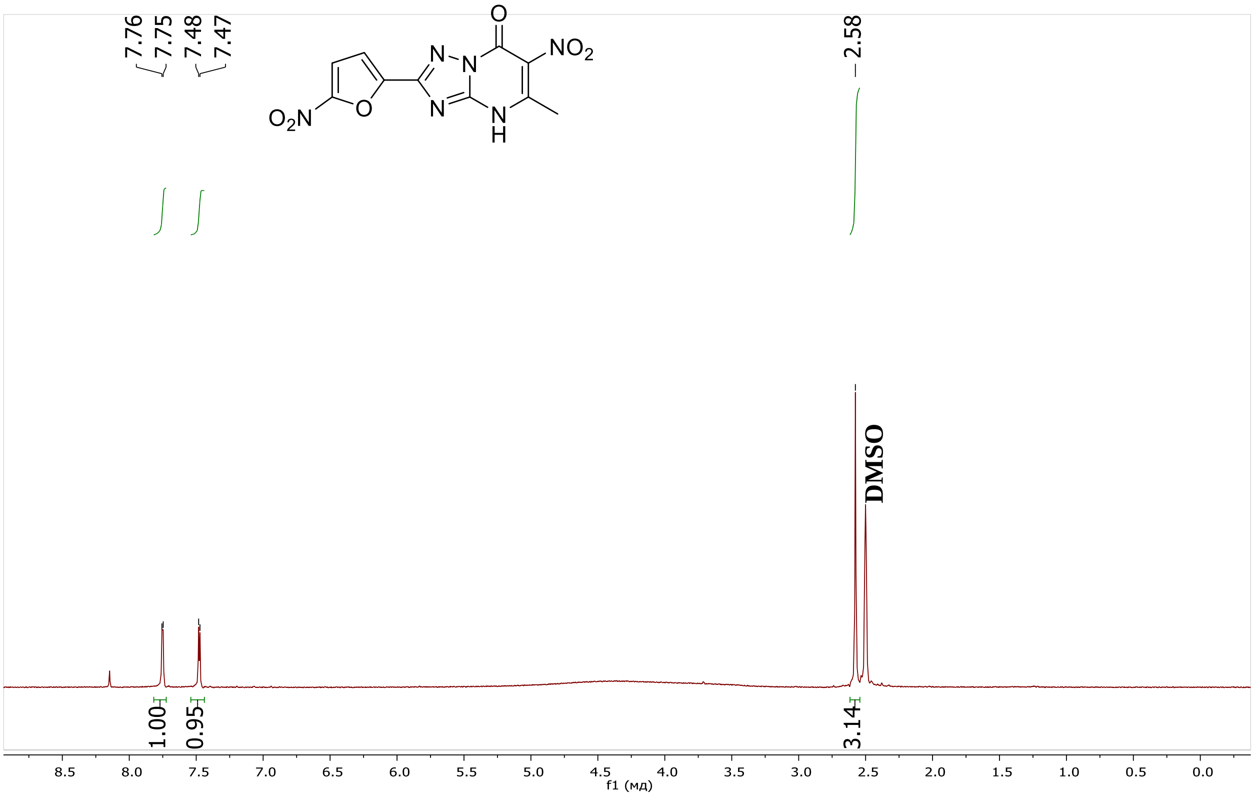

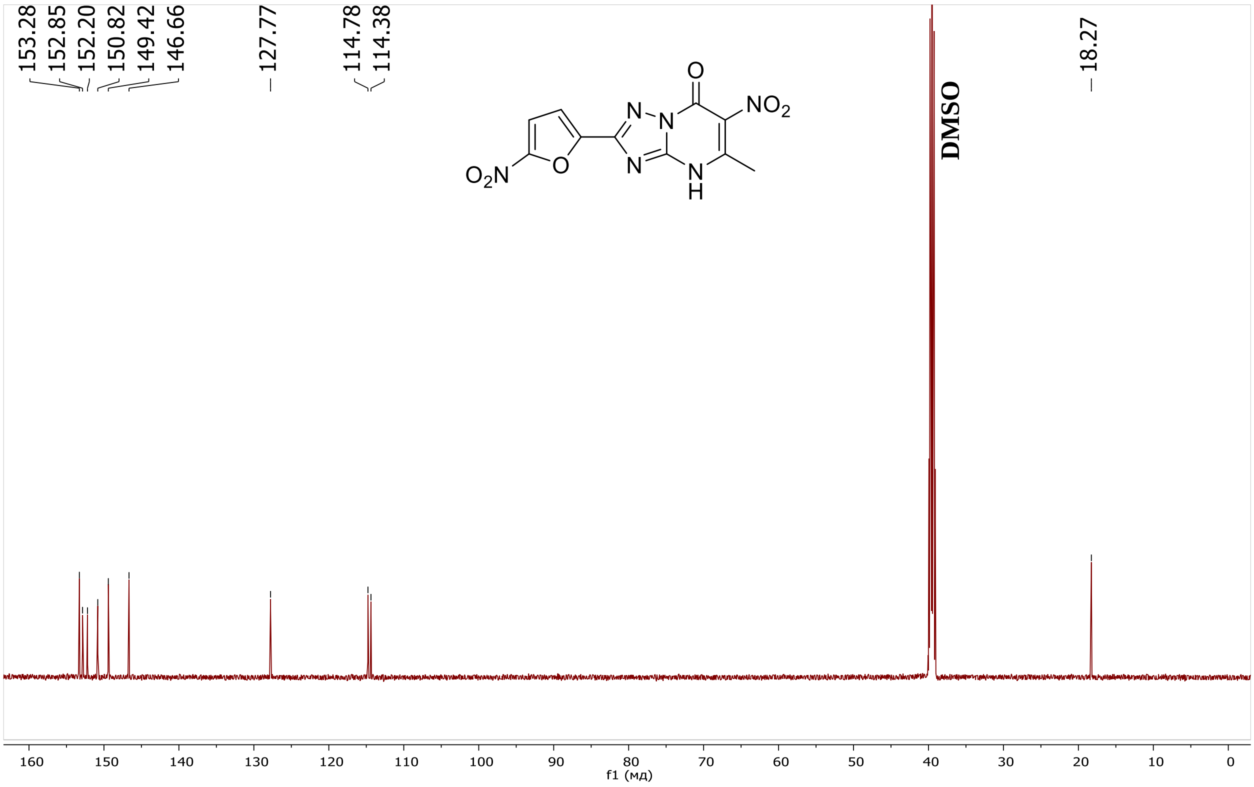

Figure S17. ^{1}H NMR (400 MHz, DMSO-*d*$_6$) and ^{13}C NMR (100 MHz, DMSO-*d*$_6$) spectra of **3m**

2-(5-nitrofur-2-yl)-5-methyl-6-nitro-1,2,4-triazolo[1,5-*a*]pyrimidin-7-one (3m)

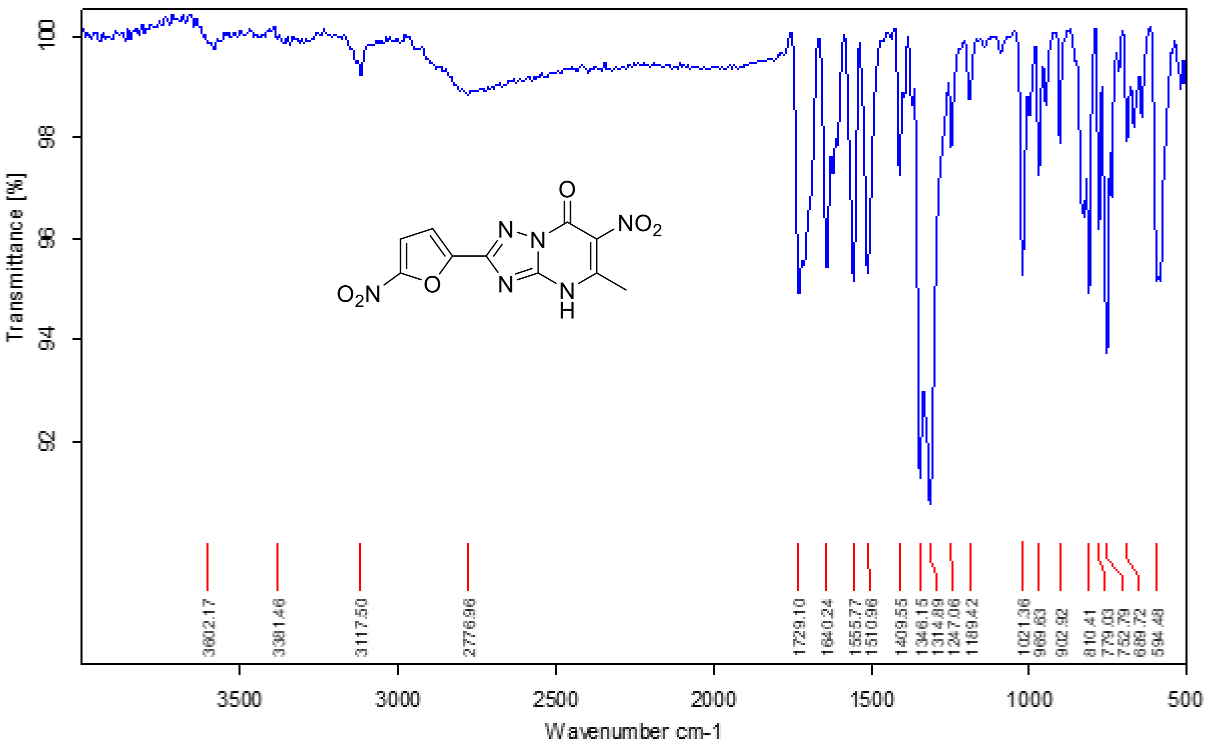

Figure S18. IR spectra of **3m**

2-(fur-2-yl)-6-nitro-1,2,4-triazolo[1,5-*a*]pyrimidin-7-one sodium salt (3n)

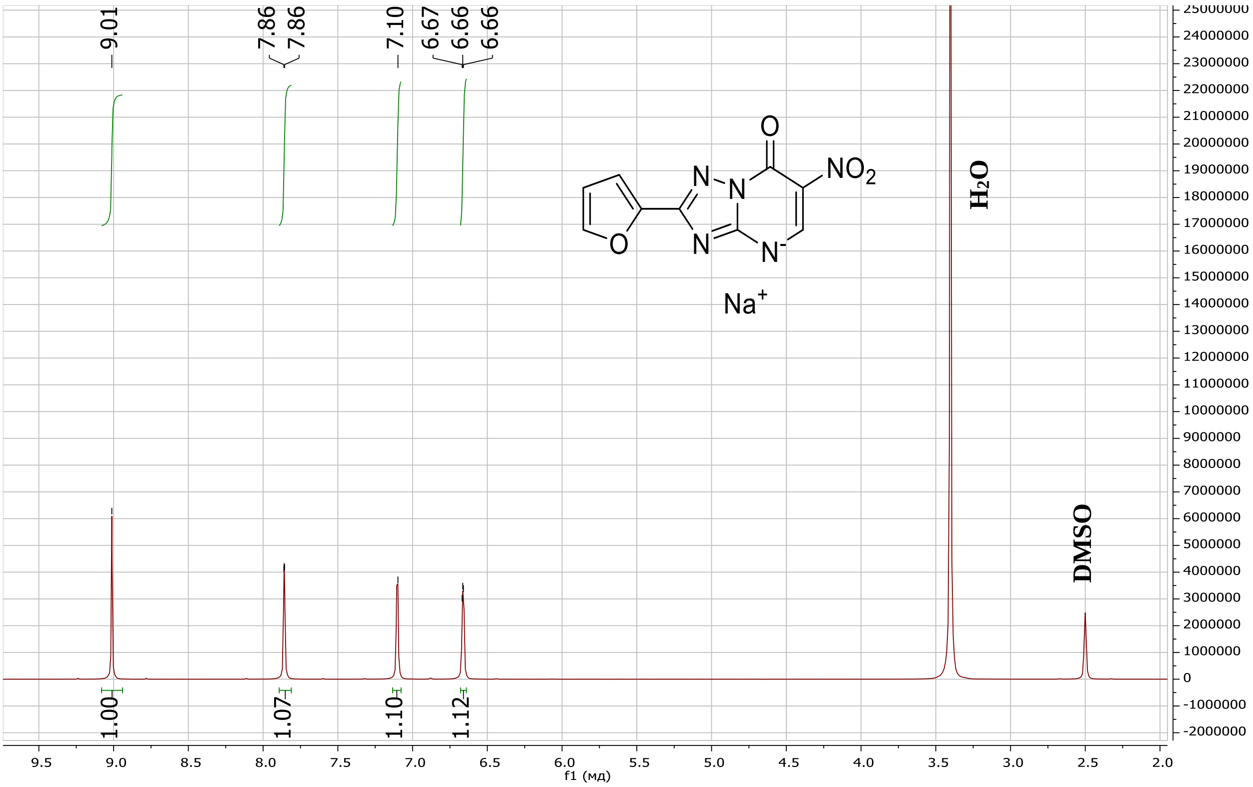

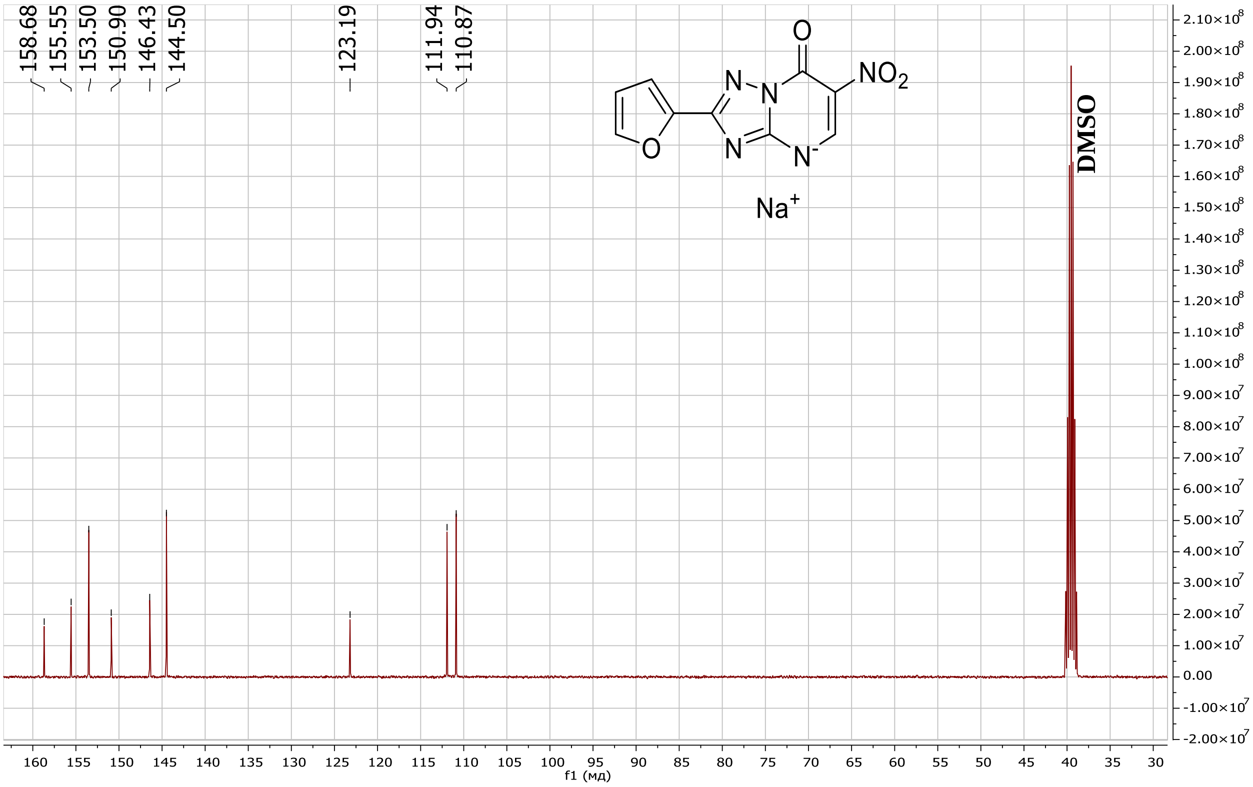

Figure S19. ^{1}H NMR (400 MHz, DMSO-d_6) and ^{13}C NMR (100 MHz, DMSO-d_6) spectra of **3n**

2-(fur-2-yl)-6-nitro-1,2,4-triazolo[1,5-*a*]pyrimidin-7-one sodium salt (3n)

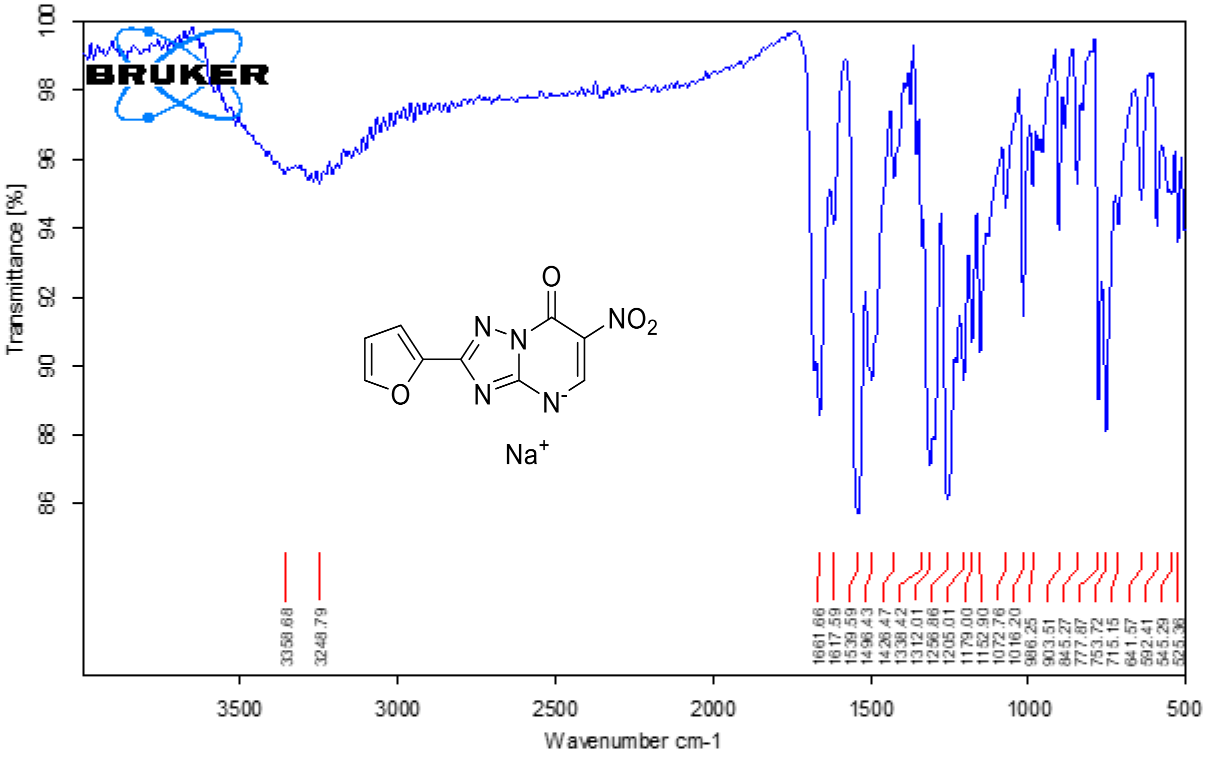

Figure S20. IR spectra of **3n**

2-(fur-2-yl)-6-nitro-1,2,4-triazolo[1,5-*a*]pyrimidin-7-one ammonium salt (3o)

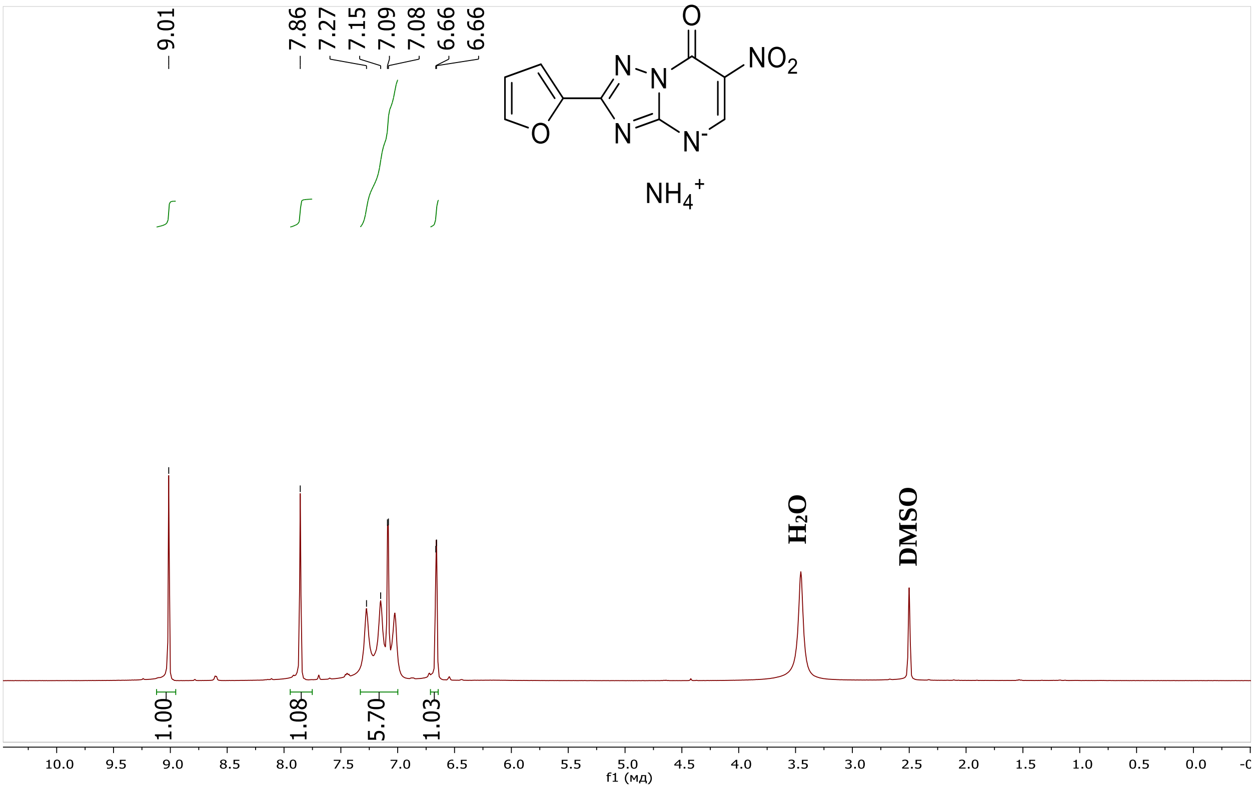

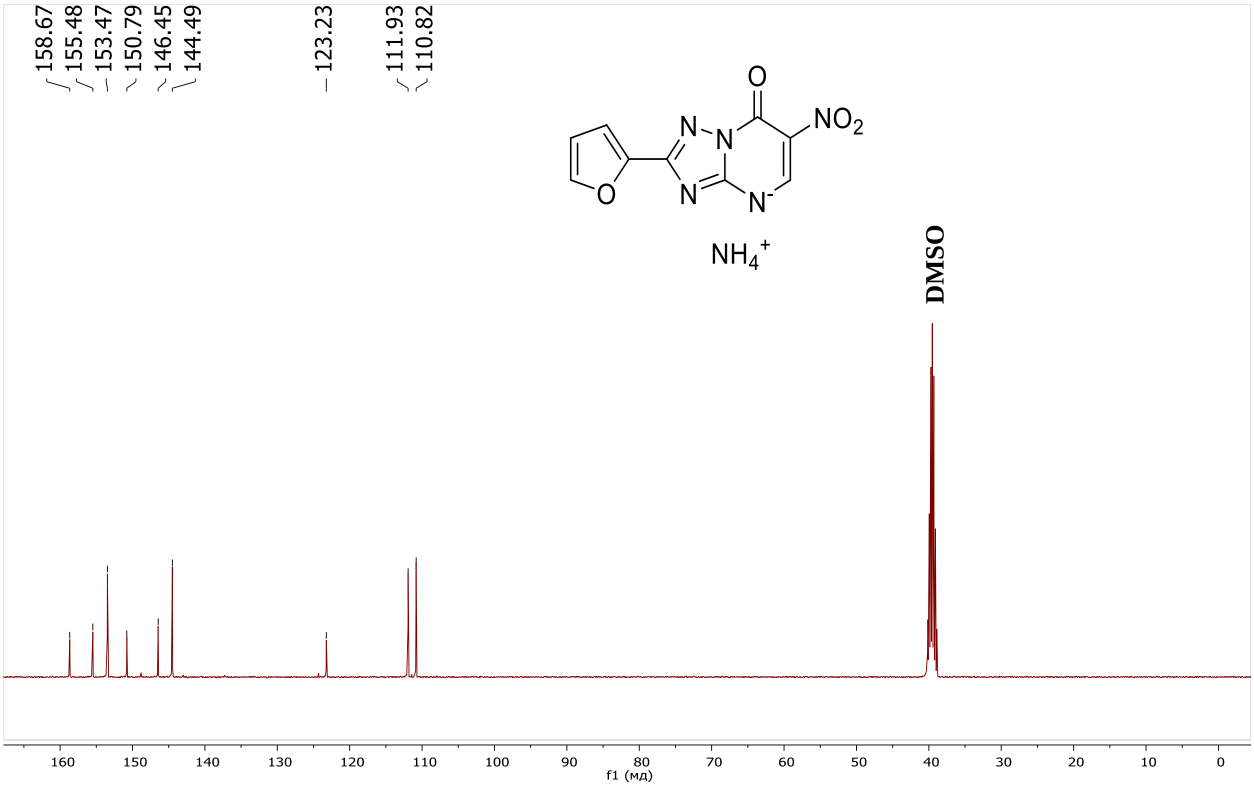

Figure S21. ^{1}H NMR (400 MHz, DMSO-d_6) and ^{13}C NMR (100 MHz, DMSO-d_6) spectra of **3o**

2-(fur-2-yl)-6-nitro-1,2,4-triazolo[1,5-*a*]pyrimidin-7-one ammonium salt (3o)

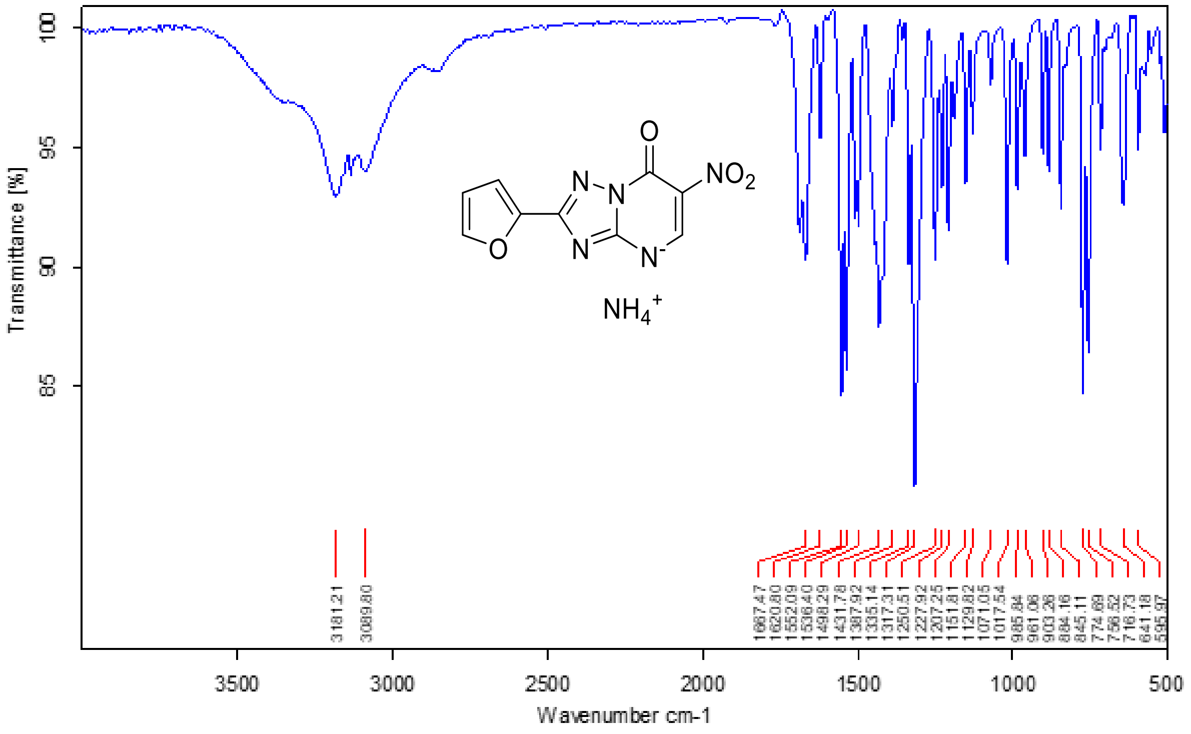

Figure S22. IR spectra of **3o**

2-(fur-2-yl)-6-nitro-1,2,4-triazolo[1,5-*a*]pyrimidin-7-one morpholinic salt (3p)

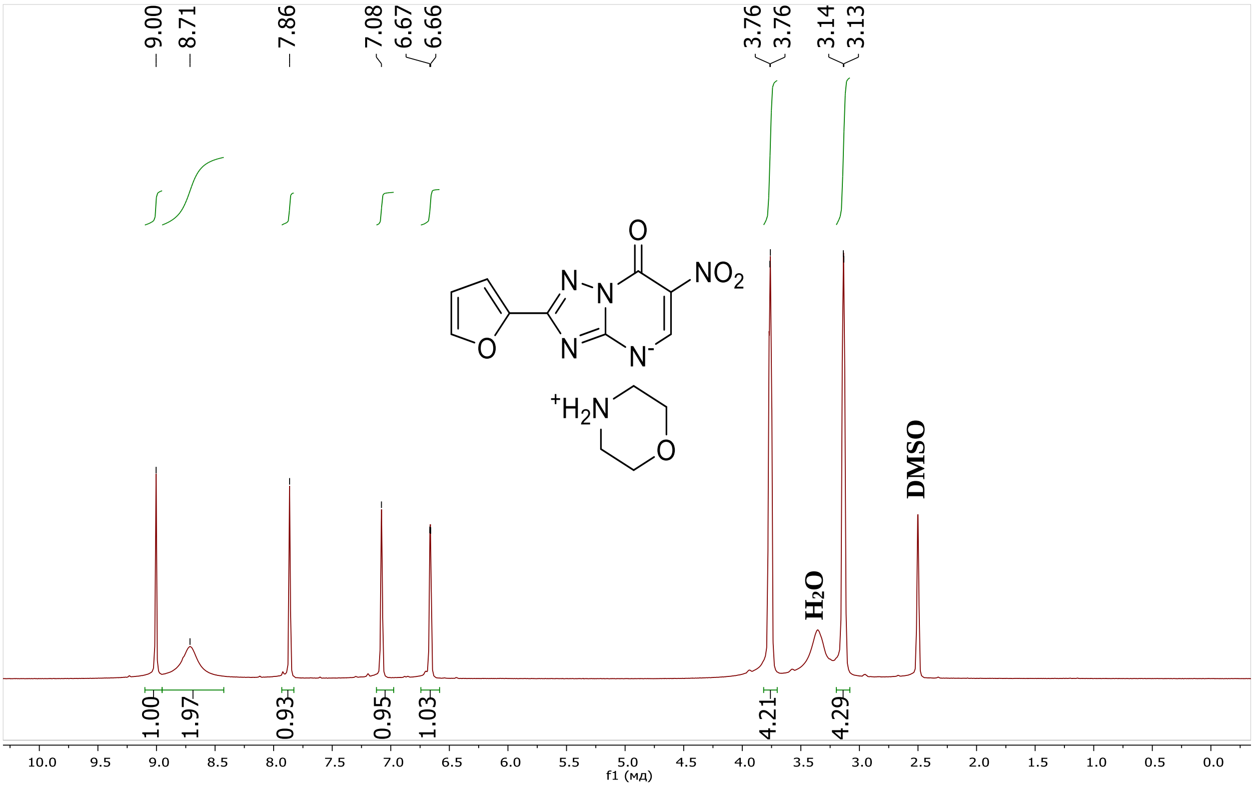

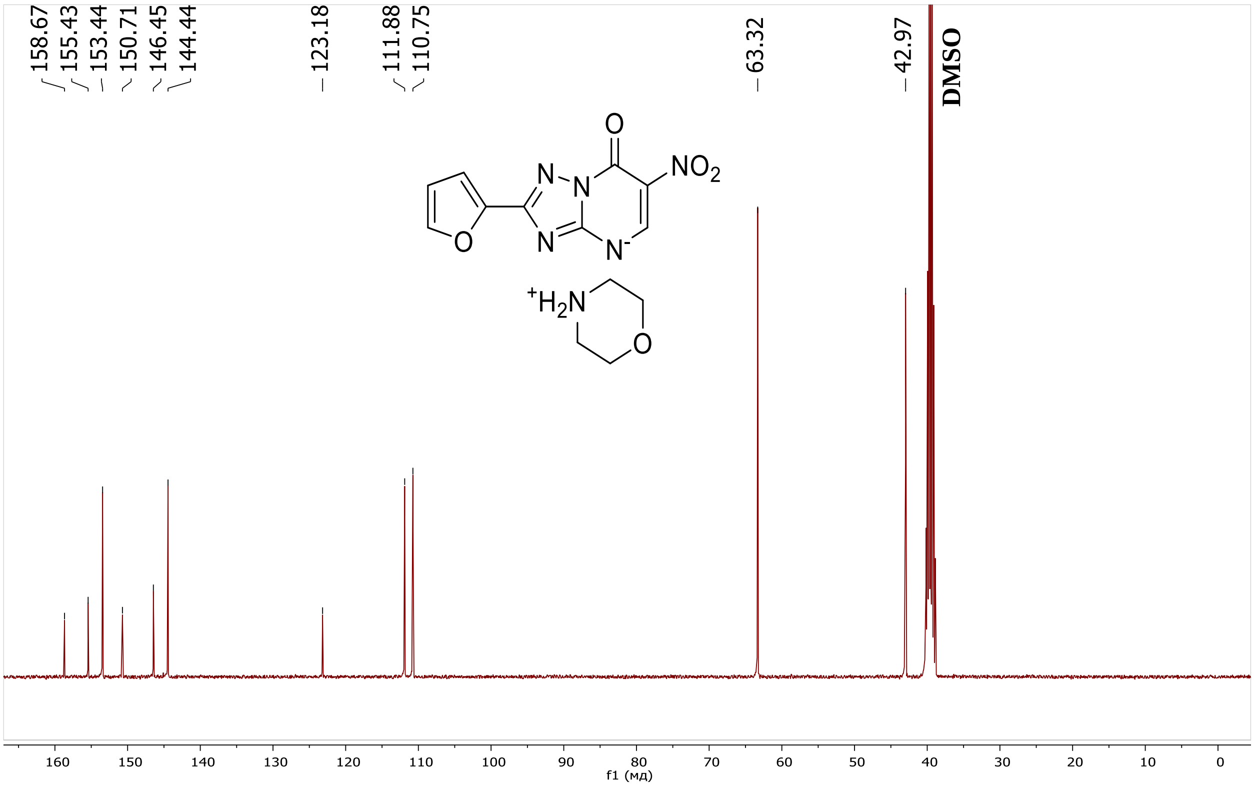

Figure S23. ^{1}H NMR (400 MHz, DMSO-*d*6) and ^{13}C NMR (100 MHz, DMSO-*d*6) spectra of **3p**

2-(fur-2-yl)-6-nitro-1,2,4-triazolo[1,5-*a*]pyrimidin-7-one morpholinic salt (3p)

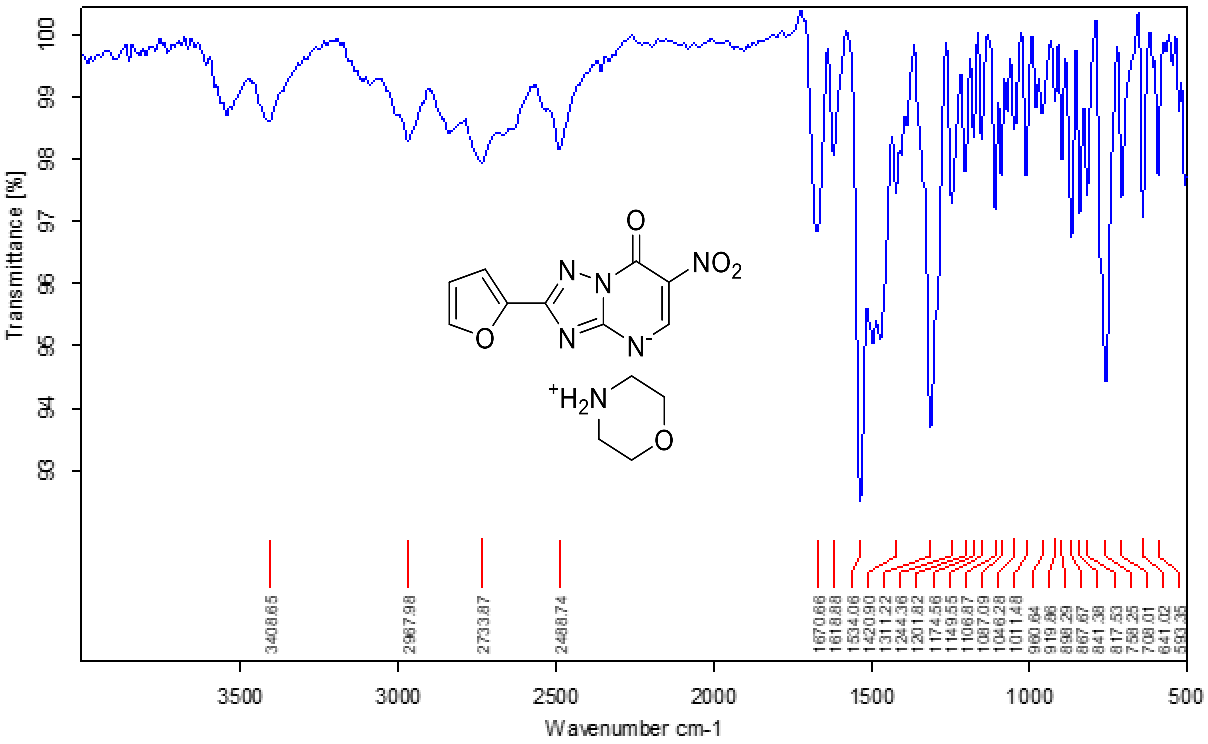

Figure S24. IR spectra of **3p**

2-(fur-2-yl)-6-nitro-1,2,4-triazolo[1,5-*a*]pyrimidin-7-one *L*-argininium salt (3q)

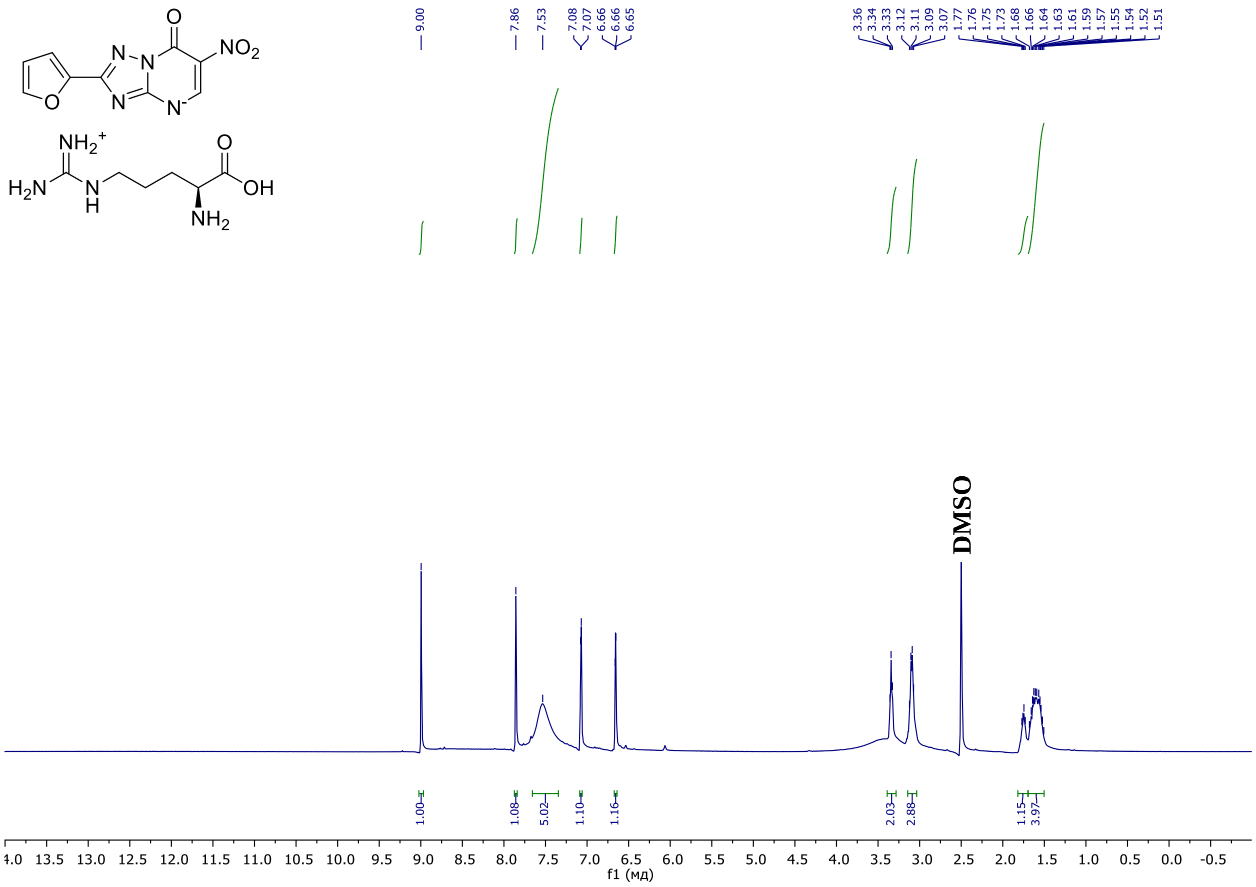

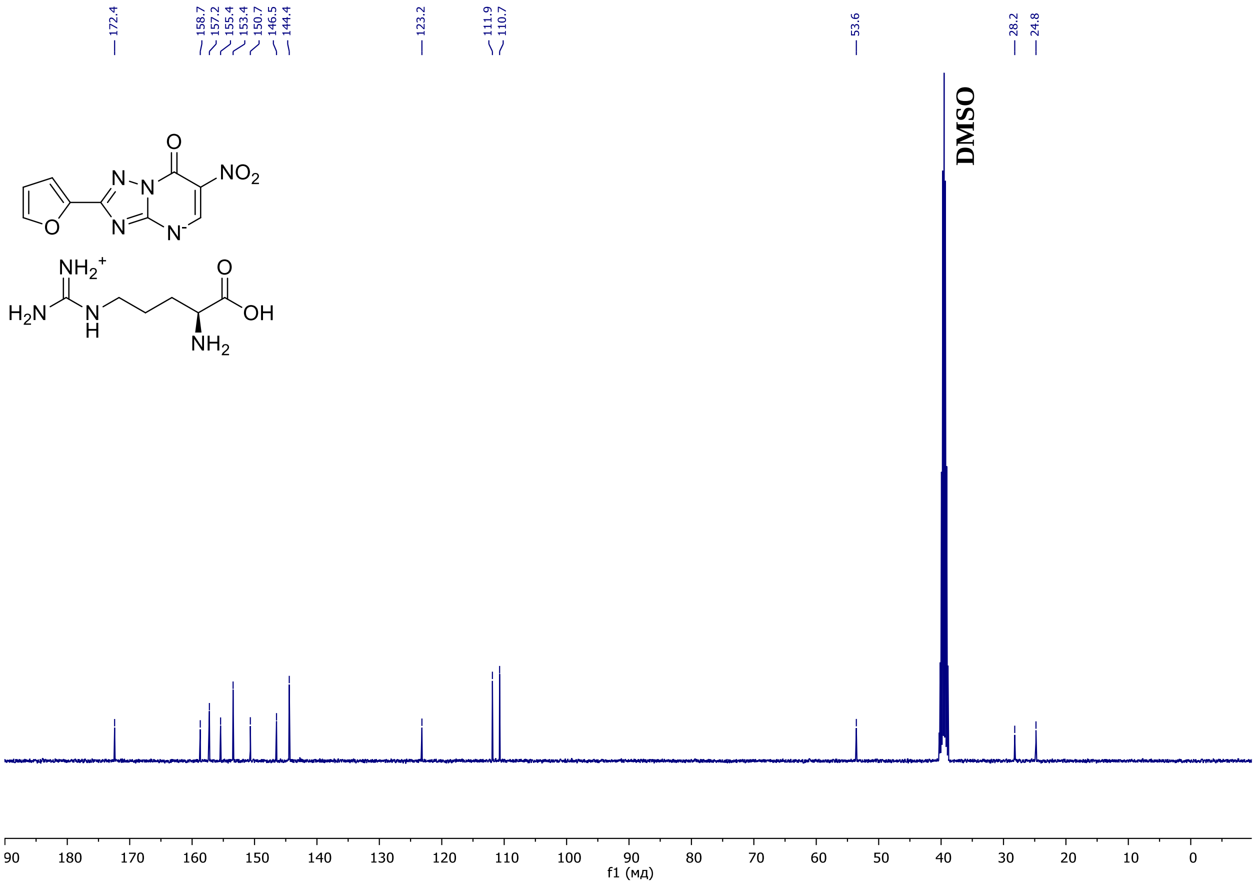

Figure S25. ^{1}H NMR (400 MHz, DMSO-d_6) and ^{13}C NMR (100 MHz, DMSO-d_6) spectra of **3q**

2-(fur-2-yl)-6-nitro-1,2,4-triazolo[1,5-*a*]pyrimidin-7-one *L*-argininium salt (3q)

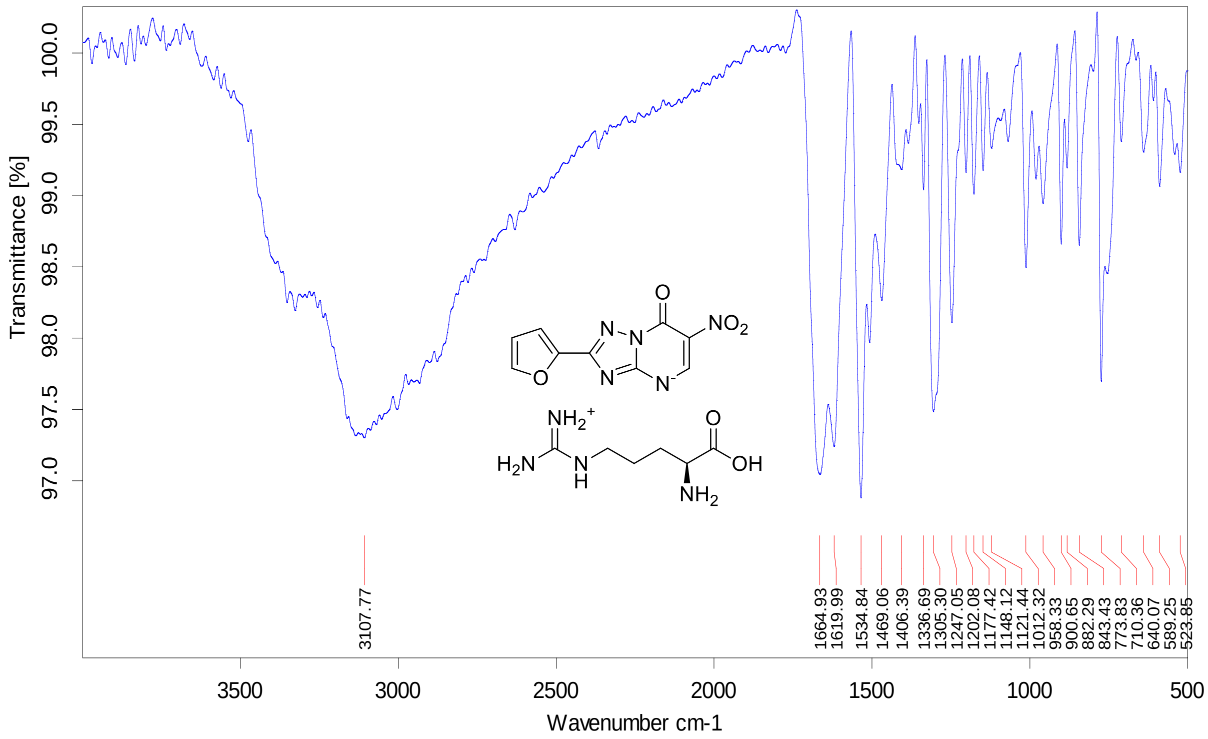

Figure S26. IR spectra of **3q**

3-(4-Hydroxyphenylethyl)-[1,3,4]thiadiazolo[3,2-*a*]purin-8-one (6a)

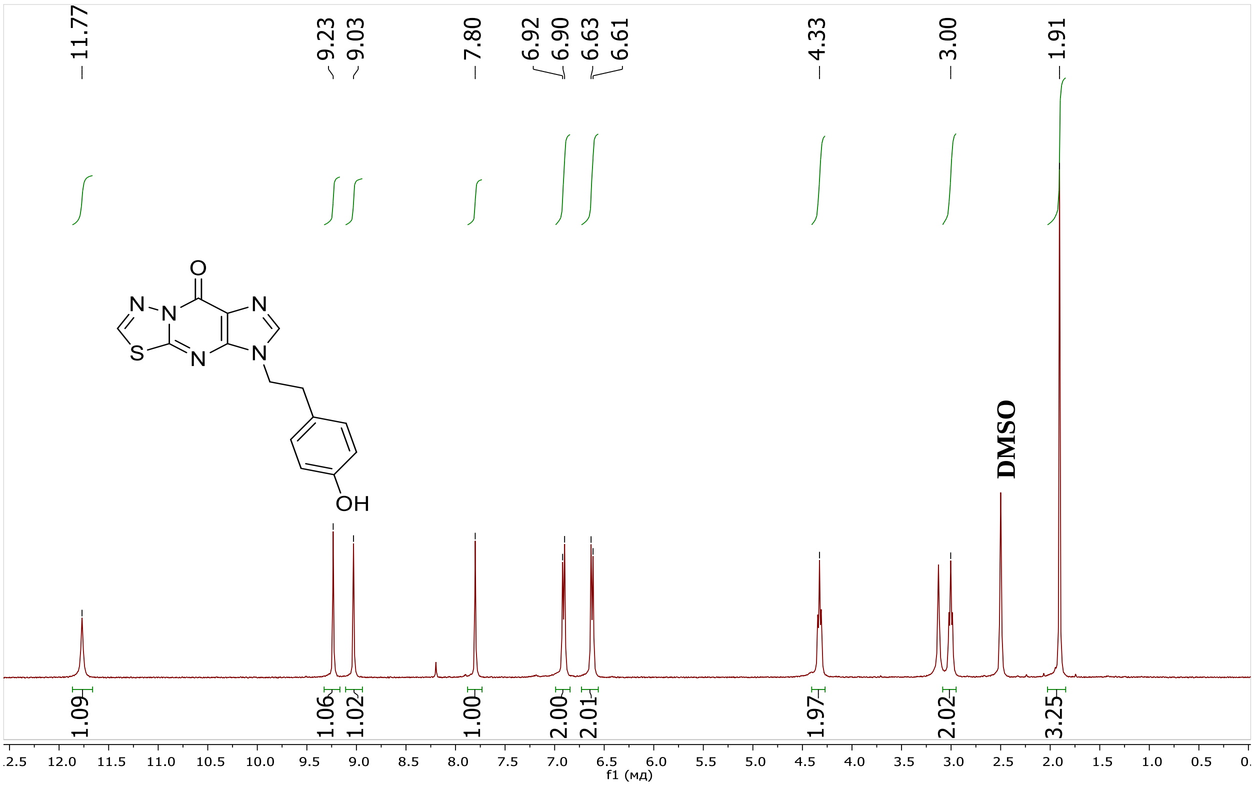

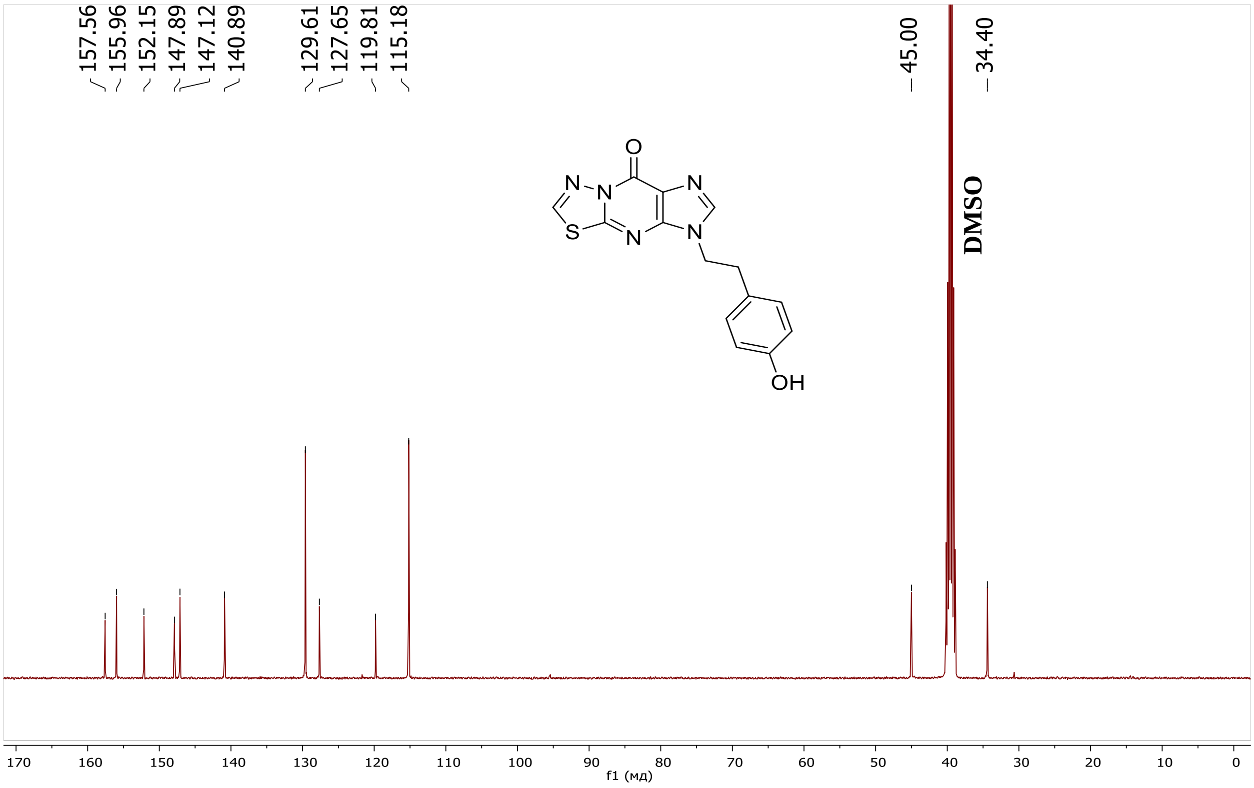

Figure S27. ^{1}H NMR (400 MHz, DMSO-d_6) and ^{13}C NMR (100 MHz, DMSO-d_6) spectra of **6b**

3-(4-Hydroxyphenylethyl)-[1,3,4]thiadiazolo[3,2-*a*]purin-8-one (6a)

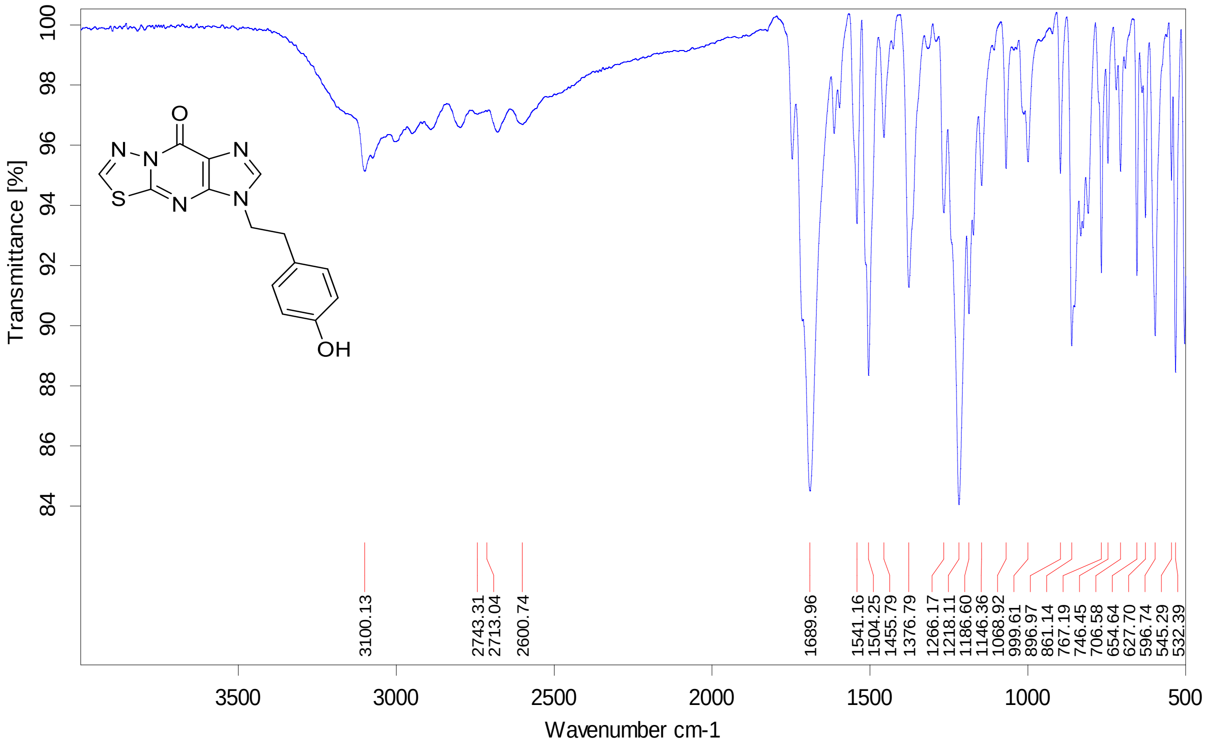

Figure S28. IR spectra of **6a**

3-(Cyclopropyl)-[1,3,4]thiadiazolo[3,2-*a*]purin-8-one (6b)

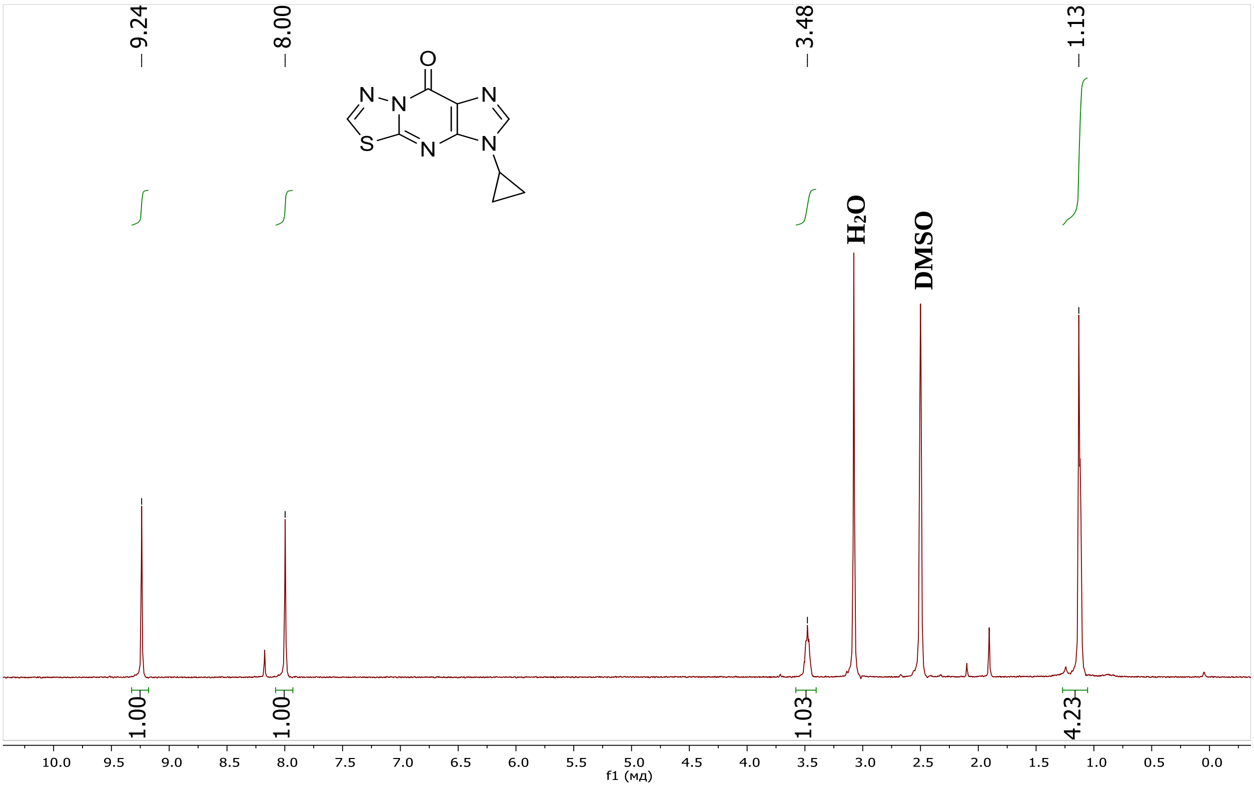

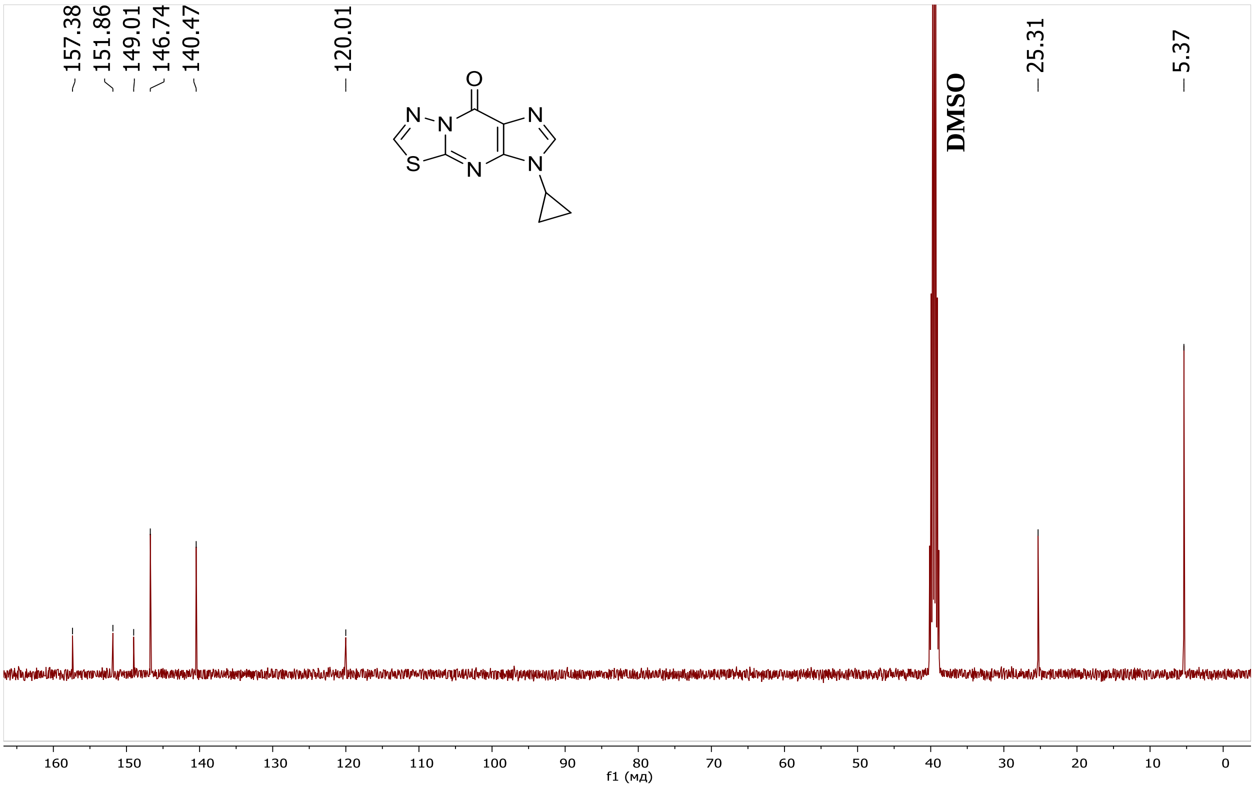

Figure S29. ^{1}H NMR (400 MHz, DMSO-d_6) and ^{13}C NMR (100 MHz, DMSO-d_6) spectra of **6b**

3-(Cyclopropyl)-[1,3,4]thiadiazolo[3,2-*a*]purin-8-one (6b)

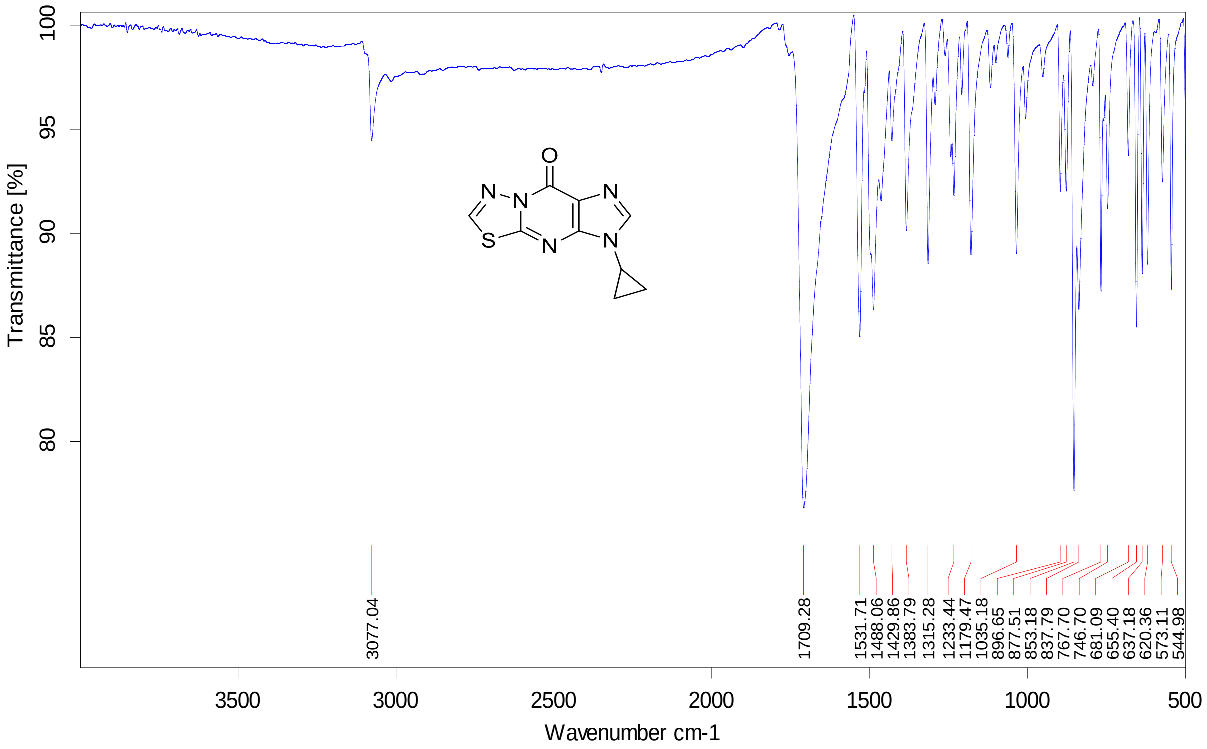

Figure S30. IR spectra of **6b**

5-[4-(diethoxymethyloxy)butyl]thiadiazolo[3,2-*a*]purin-8-one (6c)

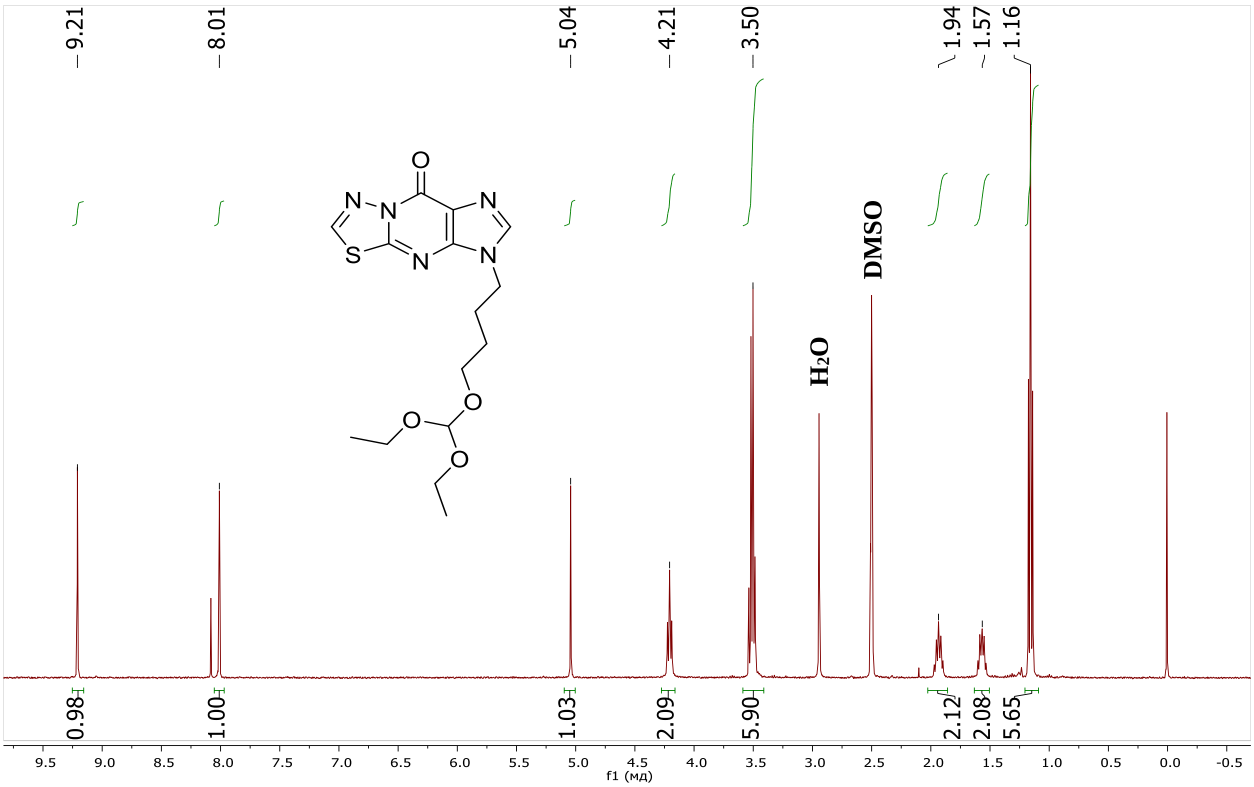

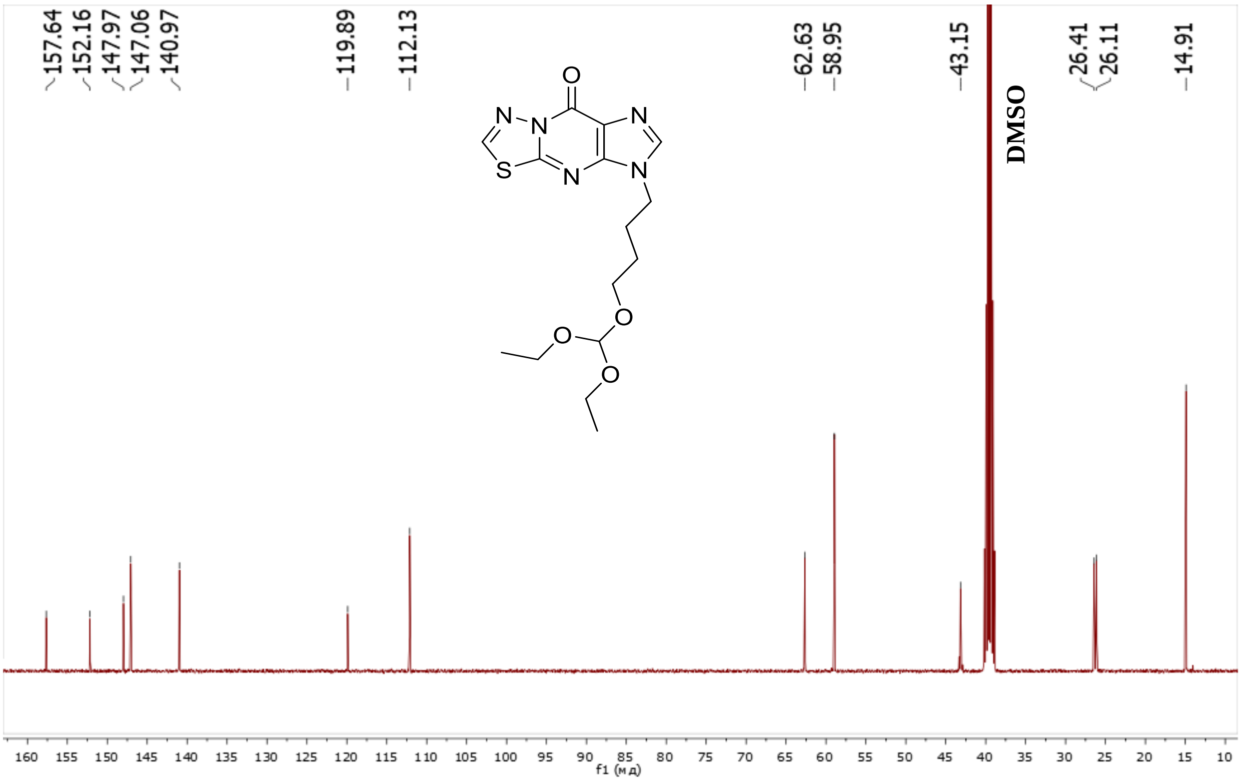

Figure S31. ^{1}H NMR (400 MHz, DMSO-d_6) and ^{13}C NMR (100 MHz, DMSO-d_6) spectra of **6c**

5-[4-(diethoxymethyloxy)butyl]thiadiazolo[3,2-*a*]purin-8-one (6c)

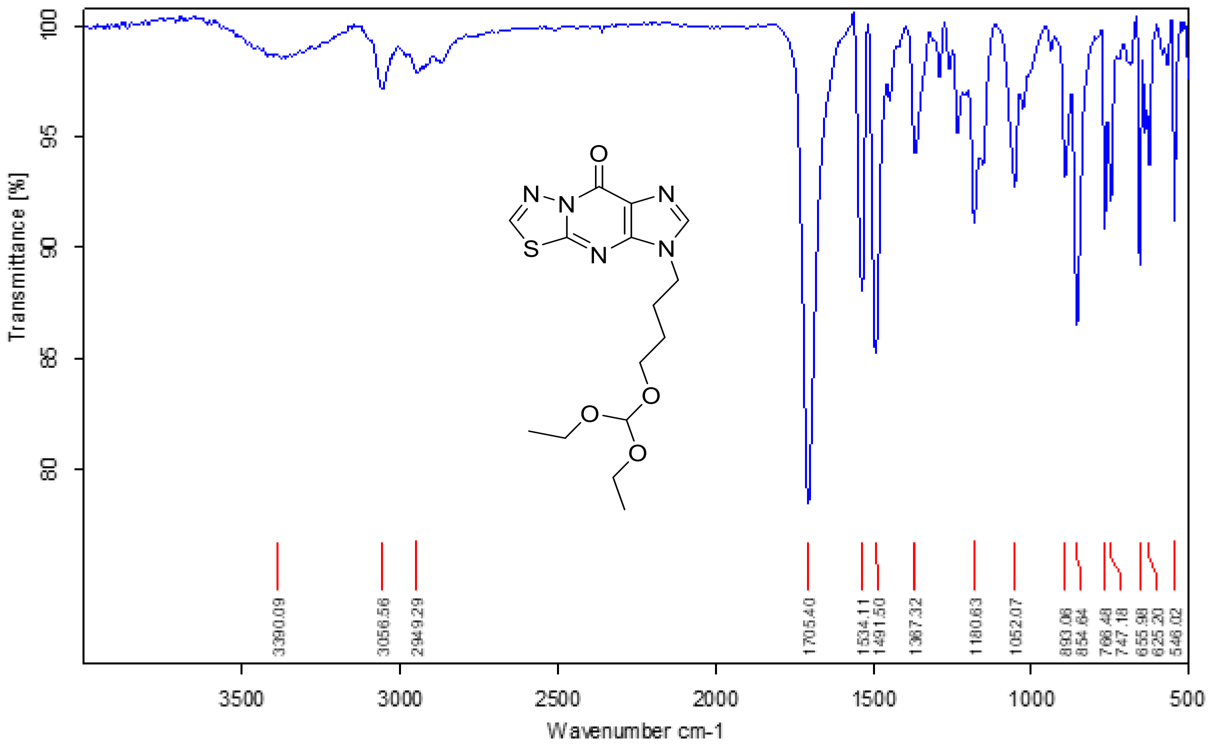

Figure S32. IR spectra of **6c**

3-nitrobenz[4,5]imidazo[1,2-*a*]pyrimidin-4(1*H*)-one sodium salt (9)

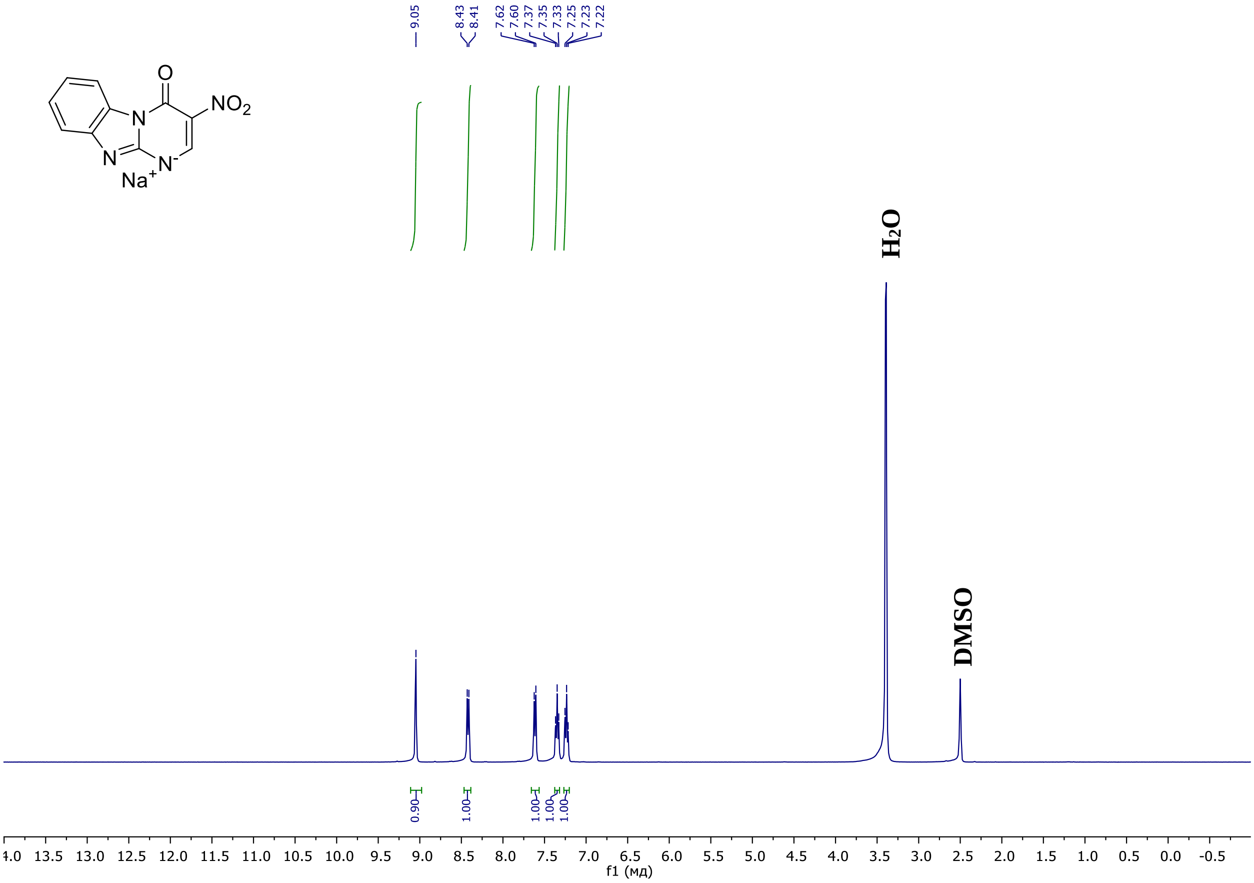

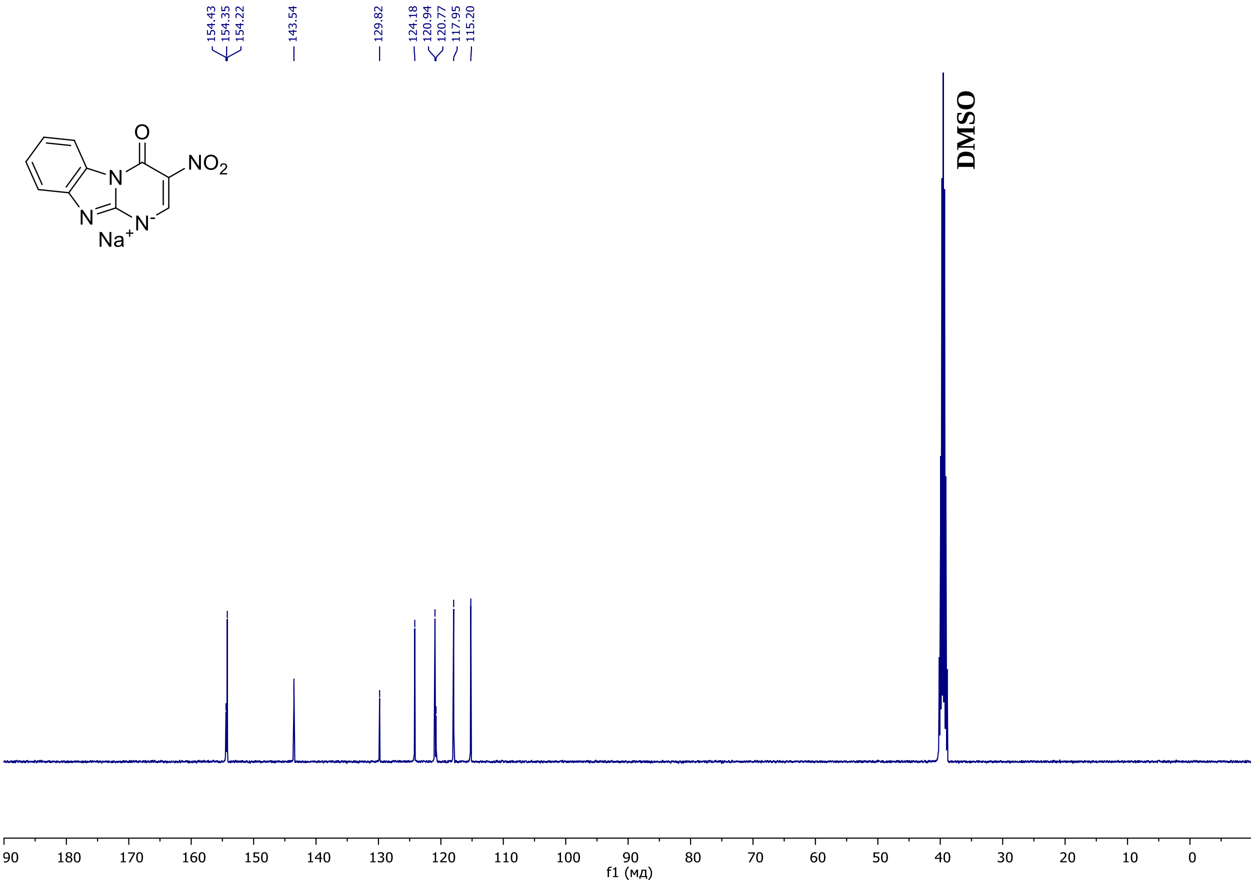

Figure S33. ^{1}H NMR (400 MHz, DMSO-d_6) and ^{13}C NMR (100 MHz, DMSO-d_6) spectra of **9**

3-nitrobenz[4,5]imidazo[1,2-*a*]pyrimidin-4(1*H*)-one sodium salt (9)

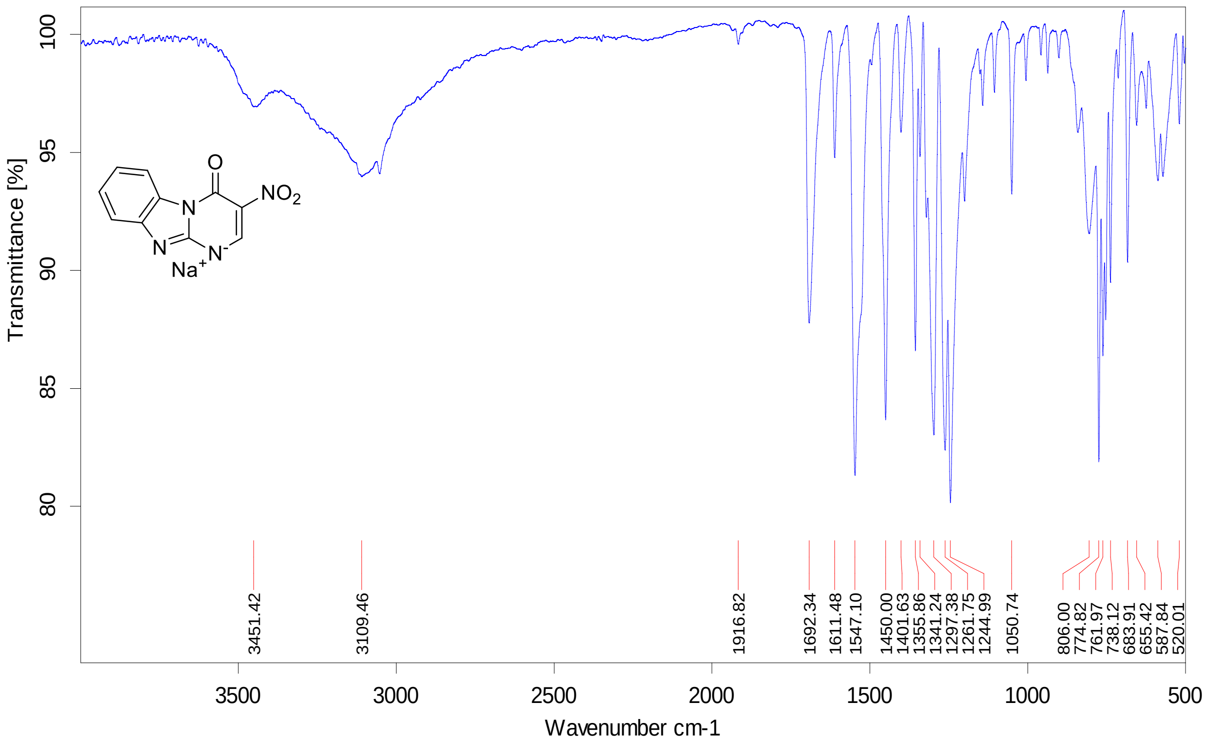

Figure S34. IR spectra of **9**

2-(*p*-Tolyl)-4-(2,3,4-trimethoxyphenyl)-2*H*-benz[4,5]imidazo[1,2-*a*][1,2,3]triazolo[4,5-*e*]pyrimidine (13a)

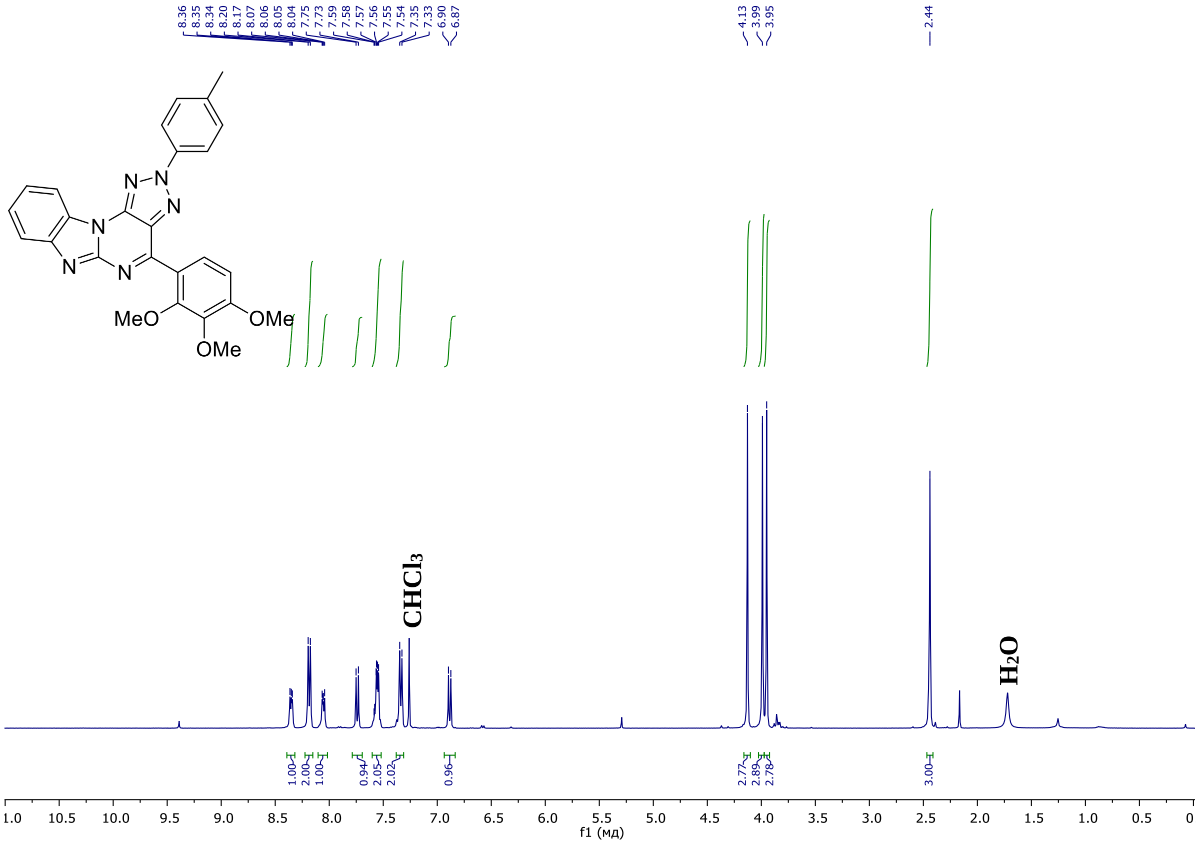

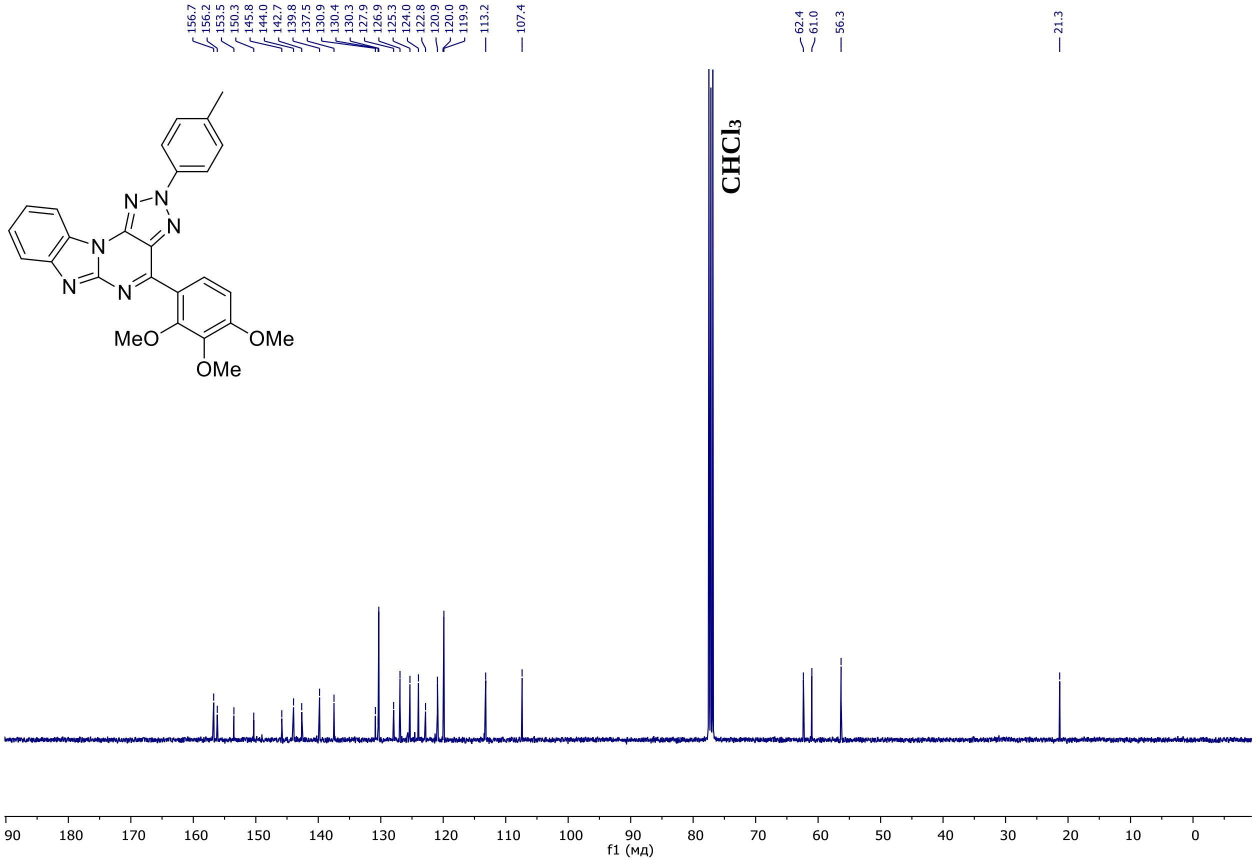

Figure S35. ^{1}H NMR (400 MHz, $CDCl_3$) and ^{13}C NMR (100 MHz, $CDCl_3$) spectra of **13a**

2-(*p*-Tolyl)-4-(2,3,4-trimethoxyphenyl)-2*H*-benz[4,5]imidazo[1,2-*a*][1,2,3]triazolo[4,5-*e*]pyrimidine (13a)

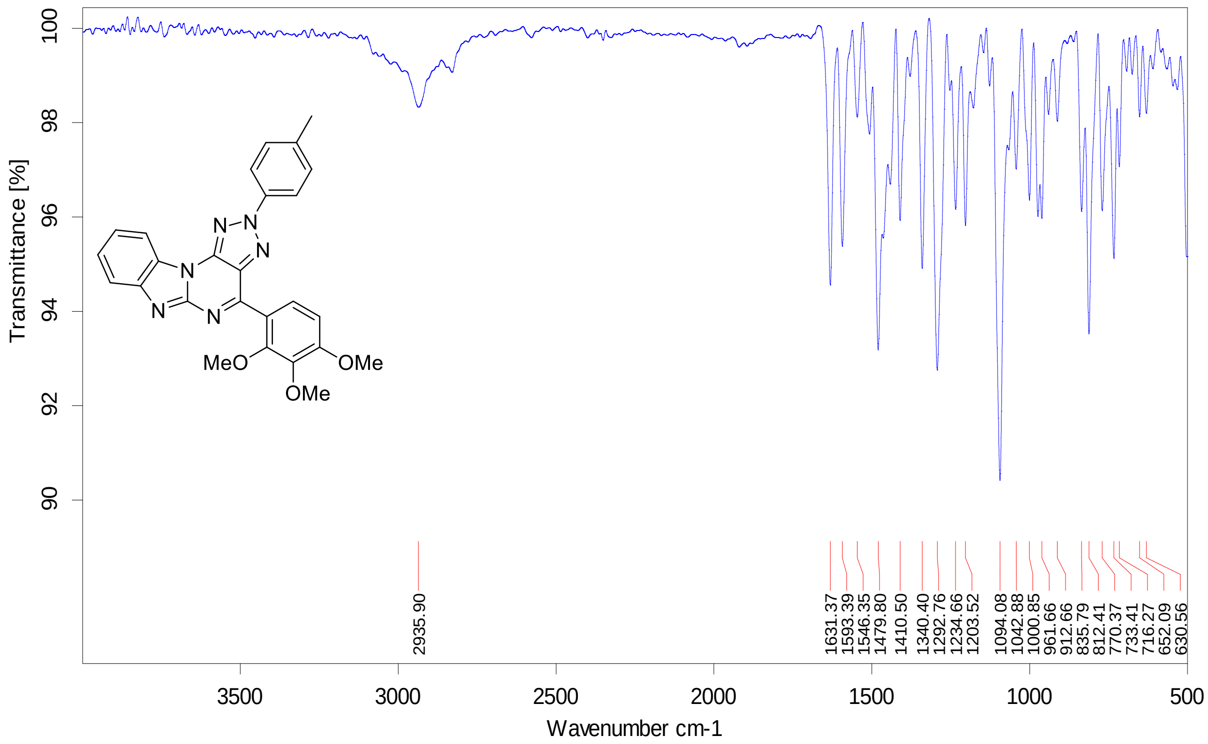

Figure S36. IR spectra of **13a**

2-(*p*-Tolyl)-4-(3,4-trimethoxyphenyl)-2*H*-benz[4,5]imidazo[1,2-*a*][1,2,3]triazolo[4,5-*e*]pyrimidine (13b)

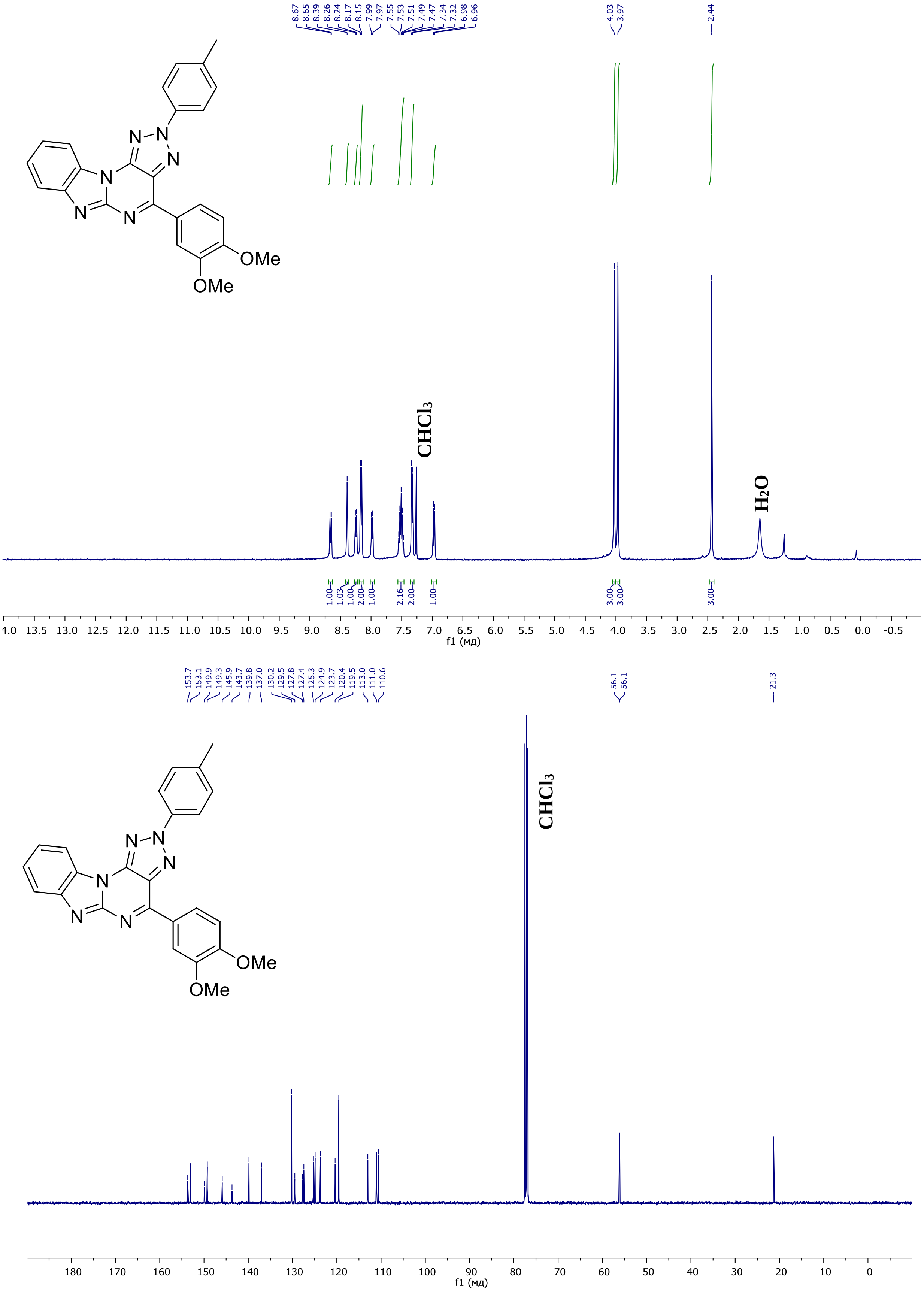

Figure S37. ^{1}H NMR (400 MHz, $CDCl_3$) and ^{13}C NMR (100 MHz, $CDCl_3$) spectra of **13b**

2-(*p*-Tolyl)-4-(3,4-trimethoxyphenyl)-2*H*-benz[4,5]imidazo[1,2-*a*][1,2,3]triazolo[4,5-*e*]pyrimidine (13b)

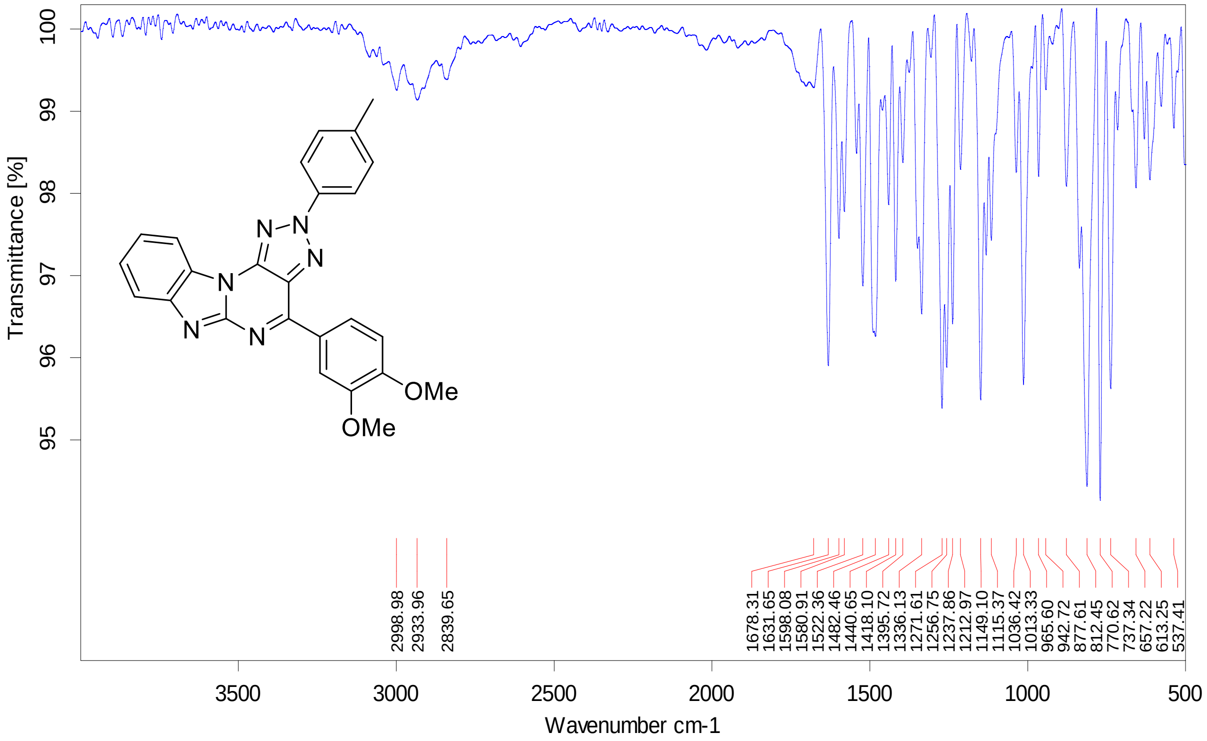

Figure S38. IR spectra of **13b**

2-(*p*-Tolyl)-4-(thien-2-yl)-2*H*-benz[4,5]imidazo[1,2-*a*][1,2,3]triazolo[4,5-*e*]pyrimidine (13c)

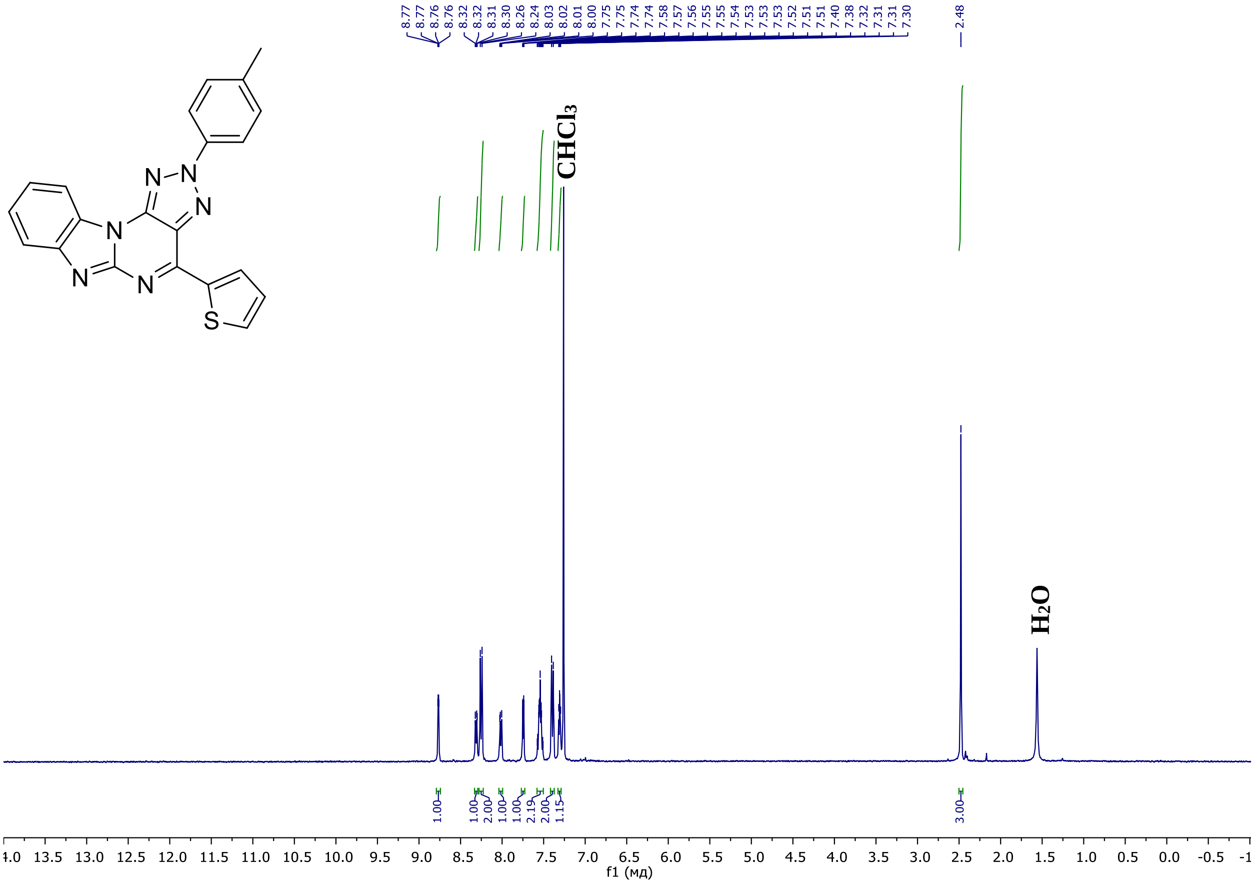

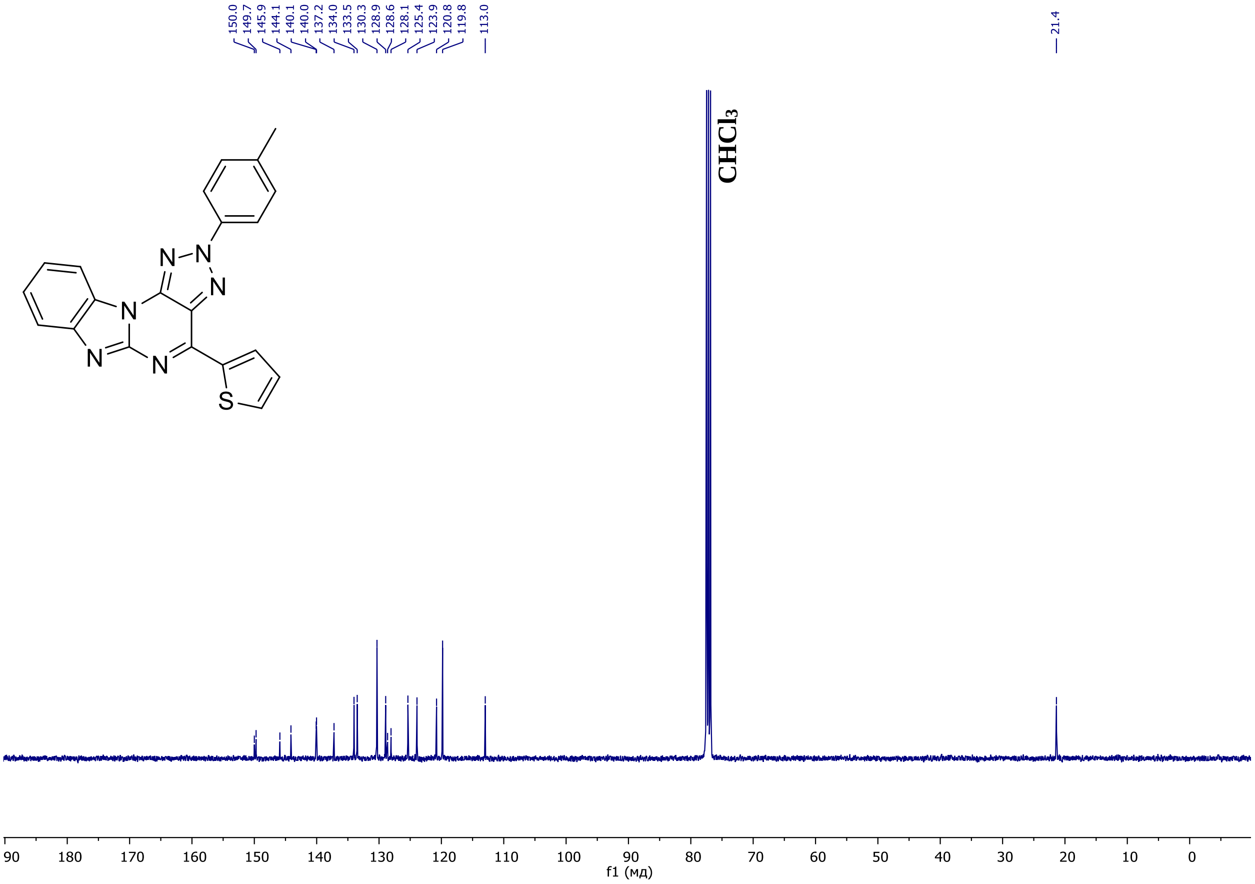

Figure S39. ^{1}H NMR (400 MHz, $CDCl_3$) and ^{13}C NMR (100 MHz, $CDCl_3$) spectra of **13c**

2-(*p*-Tolyl)-4-(thien-2-yl)-2*H*-benz[4,5]imidazo[1,2-*a*][1,2,3]triazolo[4,5-*e*]pyrimidine (13c)

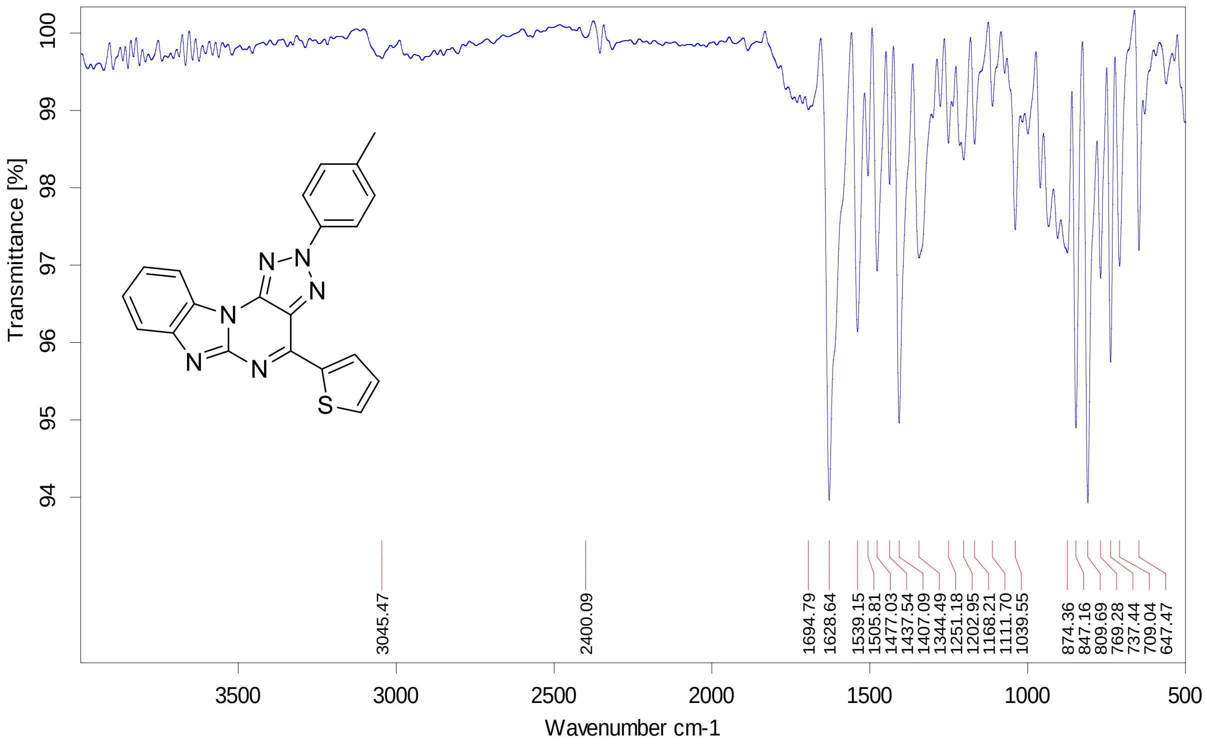

Figure S40. IR spectra of **13c**

2-(*p*-Tolyl)-4-(1-Methyl-1*H*-pyrrol-2-yl)-2*H*-benz[4,5]imidazo[1,2-*a*][1,2,3]triazolo[4,5-*e*]pyrimidine (13d)

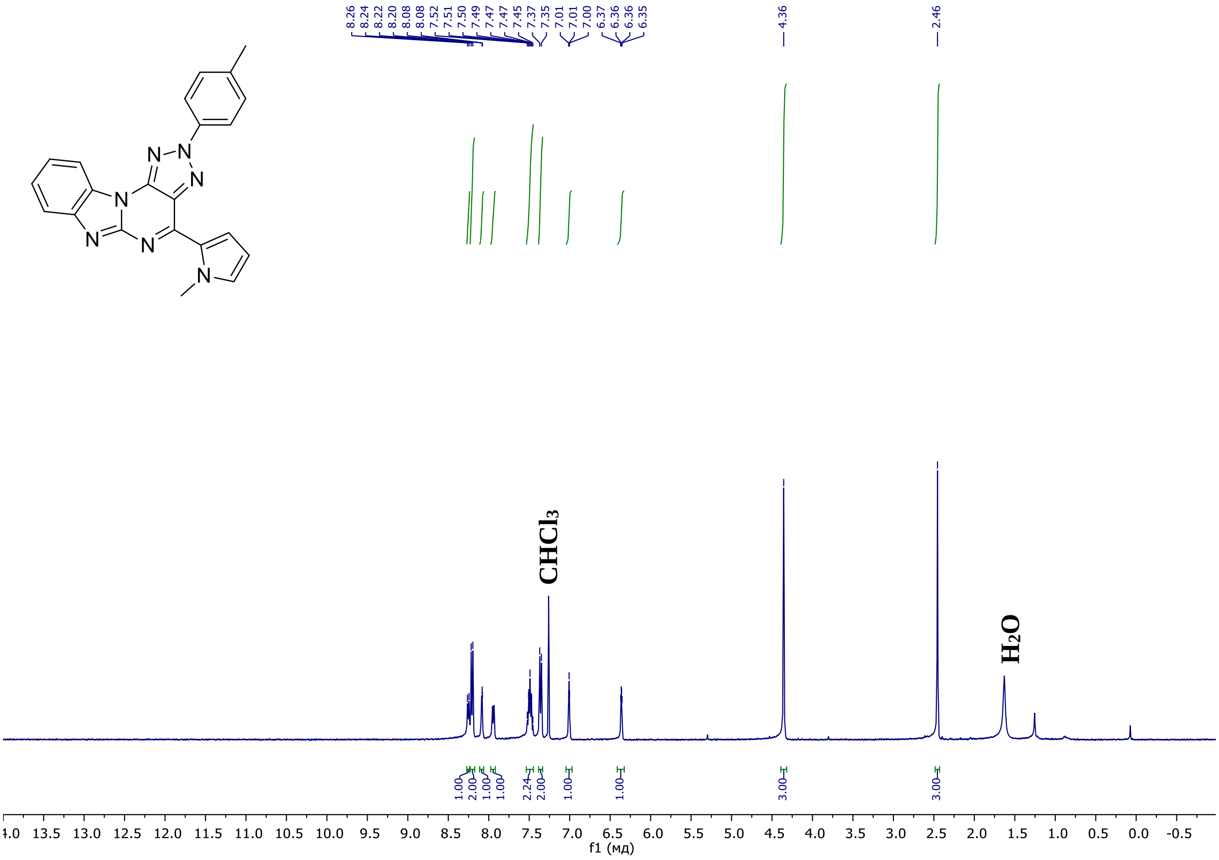

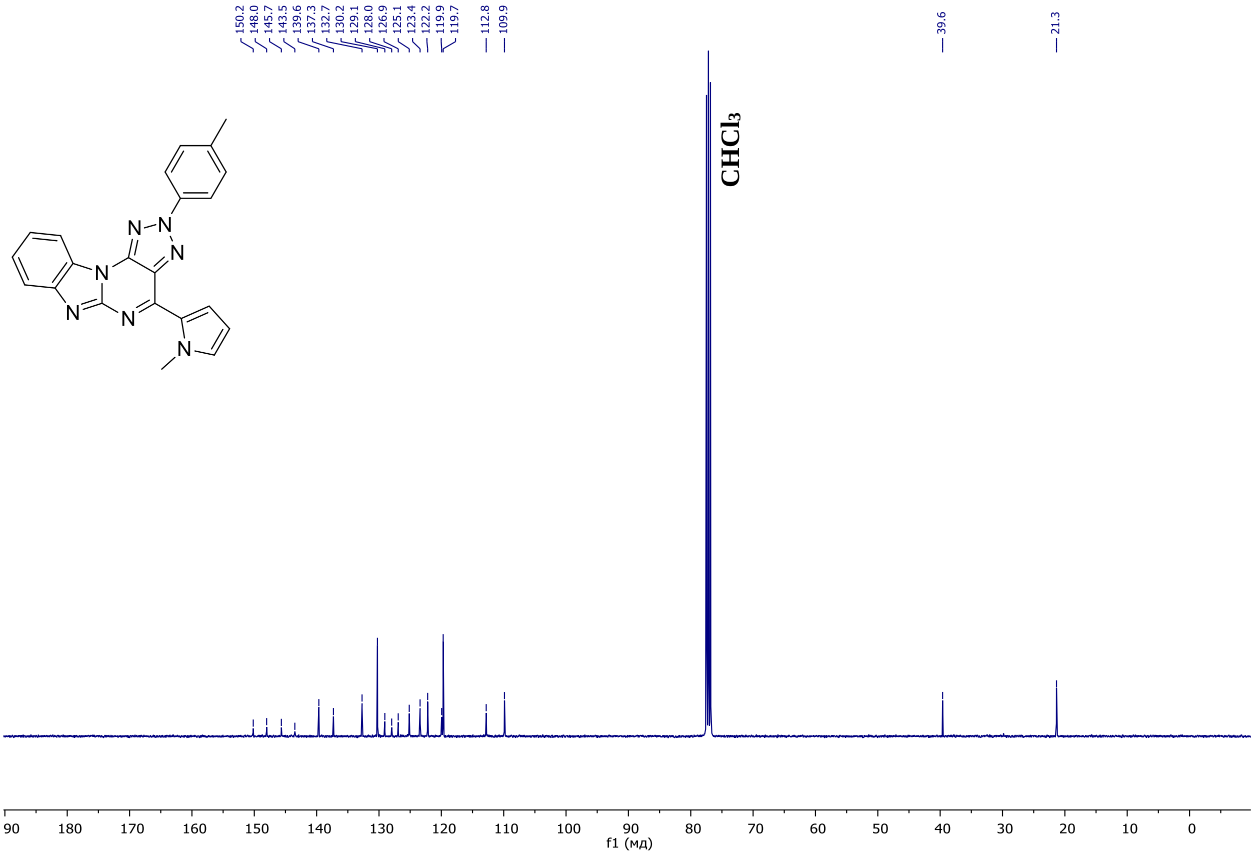

Figure S41. ^{1}H NMR (400 MHz, $CDCl_3$) and ^{13}C NMR (100 MHz, $CDCl_3$) spectra of **13d**

2-(*p*-Tolyl)-4-(1-Methyl-1*H*-pyrrol-2-yl)-2*H*-benz[4,5]imidazo[1,2-*a*][1,2,3]triazolo[4,5-*e*]pyrimidine (13d)

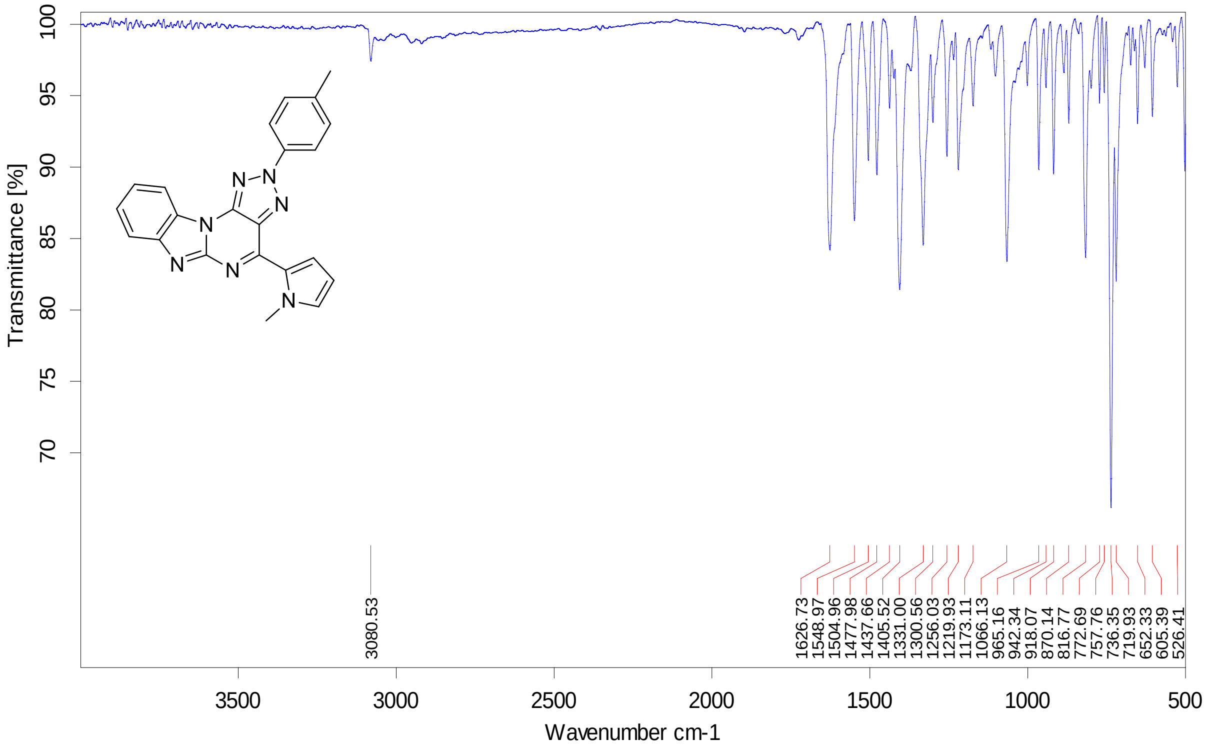

Figure S42. IR spectra of **13d**

2-(*p*-Tolyl)-4-(1-naphthalene-2-ol)-2*H*-benz[4,5]imidazo[1,2-*a*][1,2,3]triazolo[4,5-*e*]pyrimidine (13e)

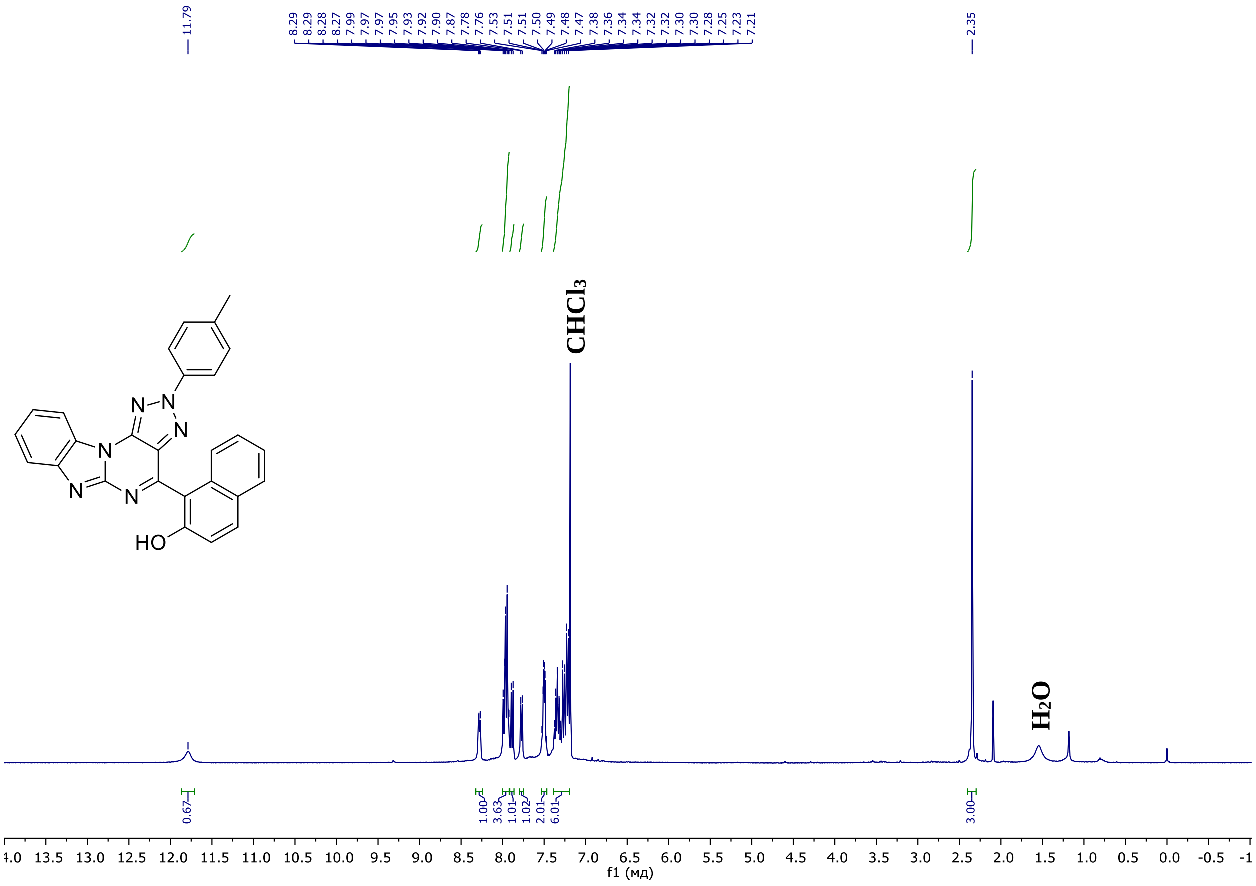

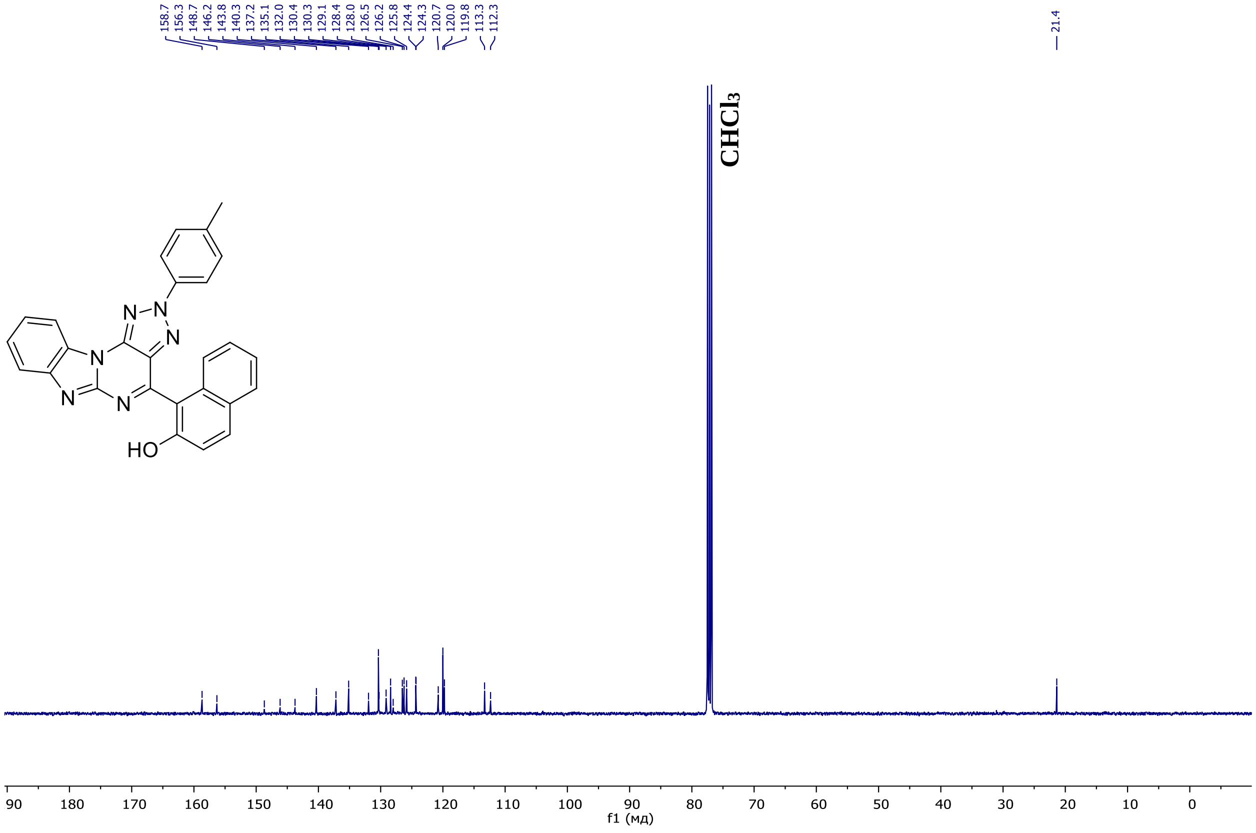

Figure S43. ^{1}H NMR (400 MHz, $CDCl_3$) and ^{13}C NMR (100 MHz, $CDCl_3$) spectra of **13e**

2-(*p*-Tolyl)-4-(1-naphthalene-2-ol)-2*H*-benz[4,5]imidazo[1,2-*a*][1,2,3]triazolo[4,5-*e*]pyrimidine (13e)

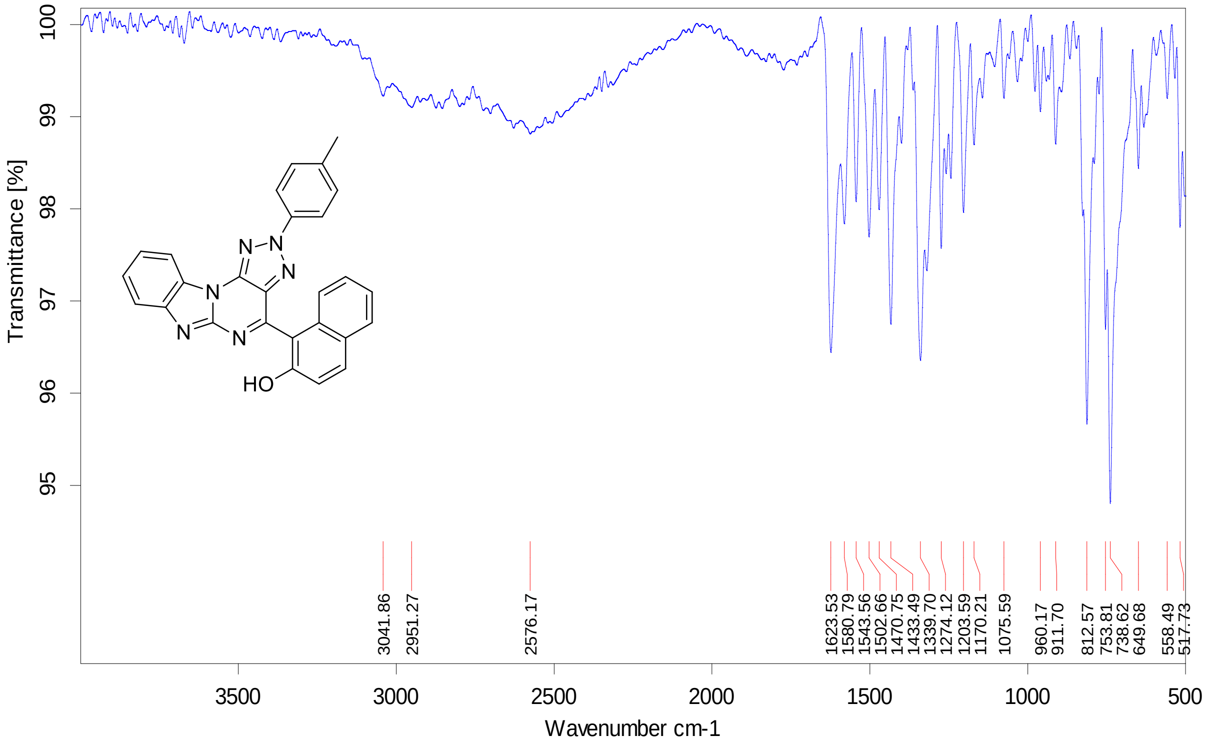

Figure S44. IR spectra of **13e**

8,9-Difluor-2-(*p*-Tolyl)-4-(2,3,4-trimethoxyphenyl)-2*H*-benz[4,5]imidazo[1,2-*a*][1,2,3]triazolo[4,5-*e*]pyrimidine (14a)

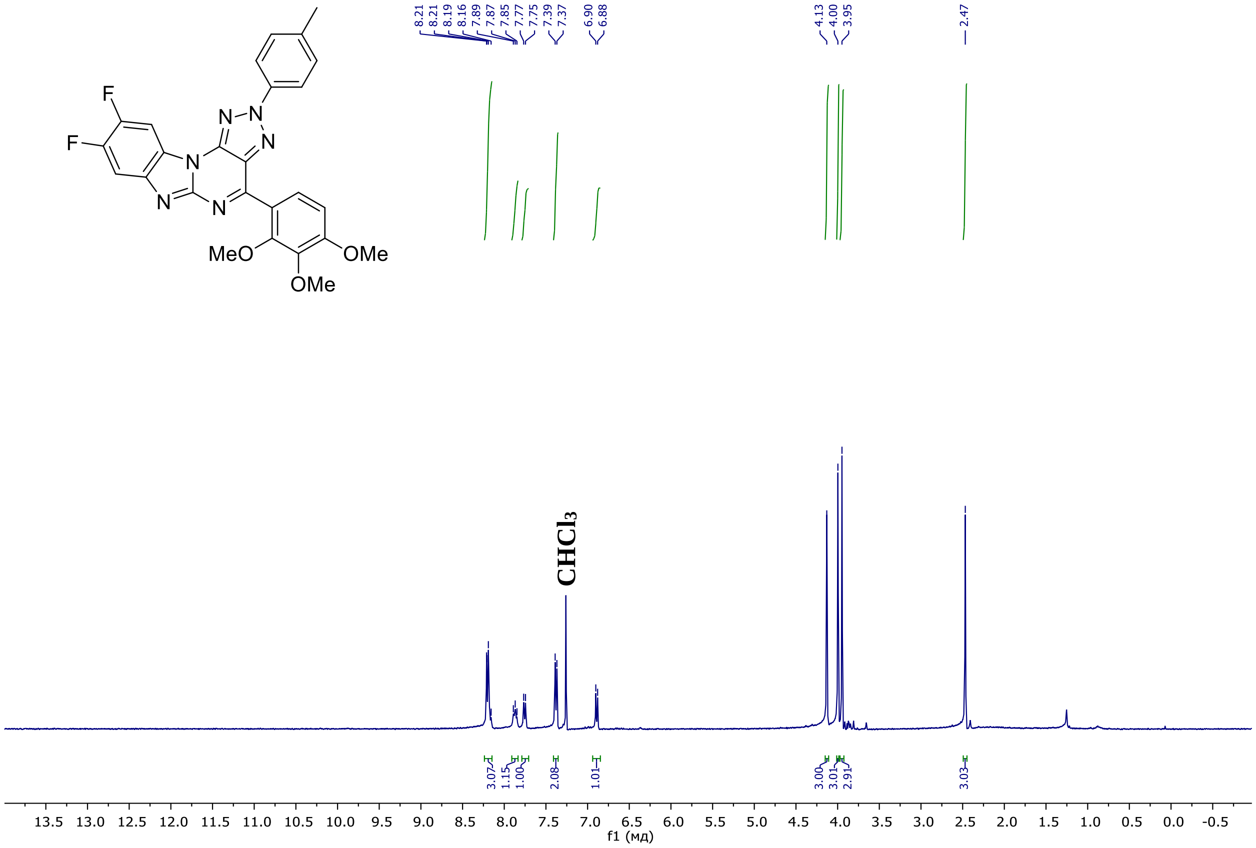

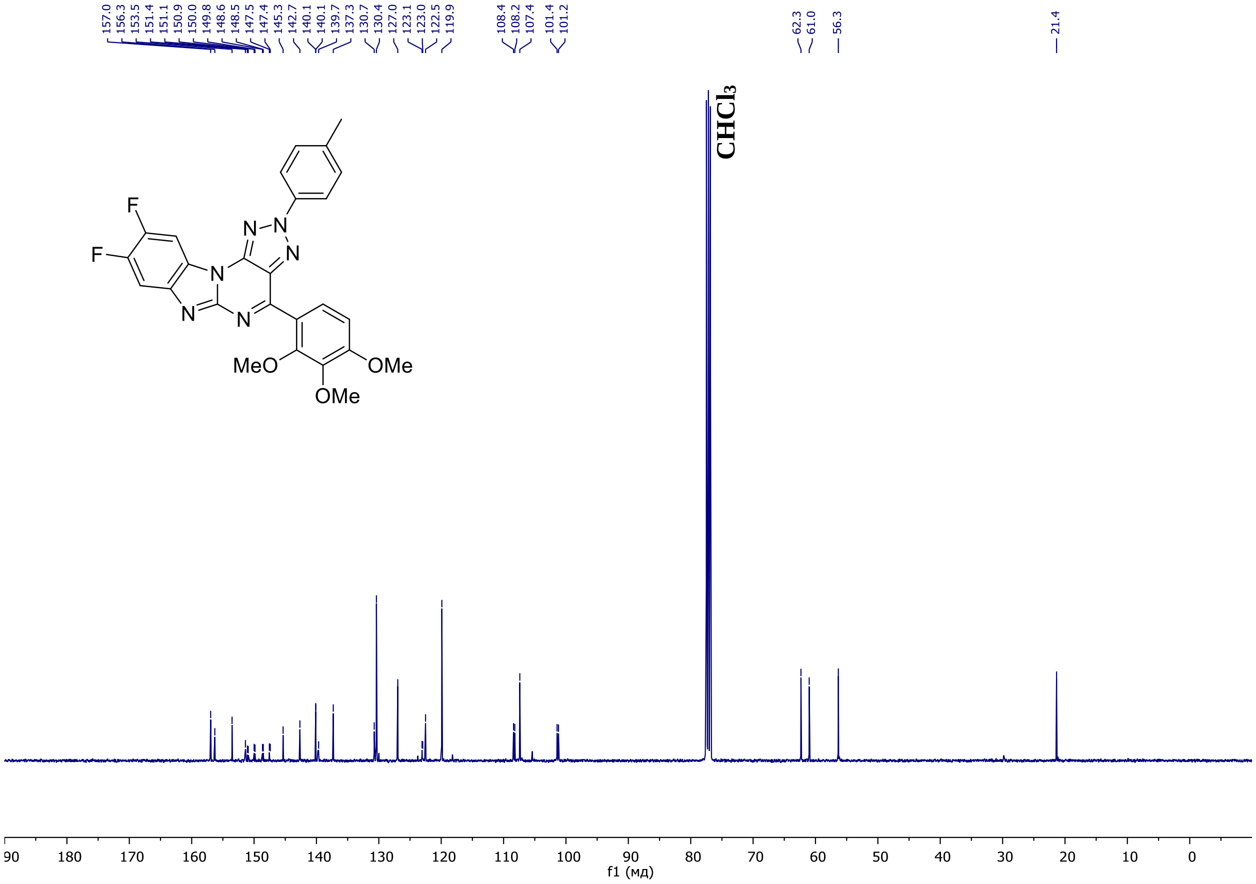

Figure S45. ^{1}H NMR (400 MHz, $CDCl_3$) and ^{13}C NMR (100 MHz, $CDCl_3$) spectra of **14a**

8,9-Difluor-2-(*p*-Tolyl)-4-(2,3,4-trimethoxyphenyl)-2*H*-benz[4,5]imidazo[1,2-*a*][1,2,3]triazolo[4,5-*e*]pyrimidine (14a)

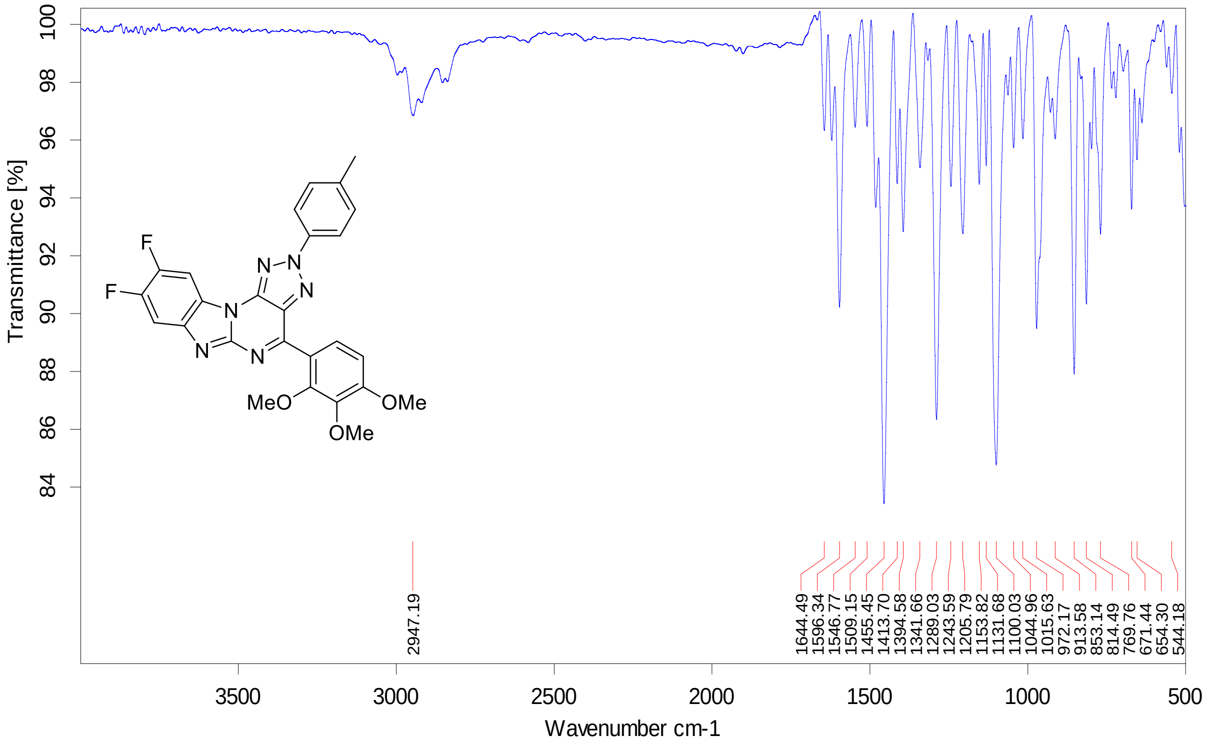

Figure S46. IR spectra of **14a**
